# The efficacy and safety of exercise and physical activity on psychosis: A systematic review and meta-analysis

**DOI:** 10.3389/fpsyt.2022.807140

**Published:** 2022-08-16

**Authors:** Christina Ziebart, Pavlos Bobos, Joy C. MacDermid, Rochelle Furtado, Daniel J. Sobczak, Michele Doering

**Affiliations:** ^1^Department of Health and Rehabilitation Sciences, Faculty of Health Sciences, Western University, London, ON, Canada; ^2^Faculty of Health Sciences, School of Physical Therapy, Western University, London, ON, Canada; ^3^Applied Health Research Centre, Li Ka Shing Knowledge Institute of St. Michael’s Hospital, Toronto, ON, Canada; ^4^Dalla Lana School of Public Health, Institute of Health Policy, Management and Evaluation, University of Toronto, Toronto, ON, Canada; ^5^Roth McFarlane Hand and Upper Limb Centre, St. Joseph’s Hospital, London, ON, Canada; ^6^Parkwood Mental Health, London, ON, Canada

**Keywords:** exercise, psychosis, physical activity, review—systematic, hospital

## Abstract

**Background:**

Treatment of psychosis typically focuses on medication, but some of these medications can have unintended side effects, exercise has global health benefits, with minimal side effects. The purpose of this systematic review and meta-analysis is to investigate the effectiveness and safety of exercise and physical activity on psychotic symptoms, in people with psychosis when compared to usual care, in a hospital setting.

**Methods:**

A systematic electronic search of the literature was performed in June 2022, in PubMed, Scopus, and PsychINFO with no date restrictions. We included randomized trials (RCTs) with patients with psychosis that received an exercise intervention within a hospital setting. The primary outcome of interest was Positive and Negative Symptom Severity Scale (PANSS) overall score. Secondary outcomes were adverse or serious adverse events.

**Results:**

A total of 24 trials were included in this systematic review, with 9 included in the meta-analysis, including 1,426 participants. Aerobic had more pronounced effects when compared to usual care in PANSS positive (−0.23, 95% CI −0.53 to 0.07), negative (−0.38, 95% CI −0.65 to −0.10), general (−0.42, 95% CI −0.71 to −0.13) and overall scores (−0.25, 95% CI −0.52 to 0.03). Yoga when compared to usual care had no difference in PANSS subscale and overall scores. We found no difference on relapsing of psychiatric symptoms or somatic hospitalization when we compared aerobic or yoga to usual care (Risk Ratio, 1.12 95% CI 0.44–2.81).

**Conclusion:**

Aerobic activity as an exercise modality in a hospital setting can be effective in decreasing negative and general psychosis symptom severity scores compared to usual care, however, it was uncertain if the effects were clinically important. More trials are needed to confirm the clinically benefit of aerobic exercise.

**Systematic Review Registration:**

[https://www.crd.york.ac.uk/prospero/], identifier [CRD42021224997].

## Introduction

Psychosis can be part of a variety of conditions including, but not limited to, schizophrenia, schizoaffective disorder, bipolar disorder and major depressive disorder (1). Schizophrenia affects approximately 24 million people worldwide and is characterized by positive symptoms such as hallucinations and delusions, negative symptoms including alogia and avolition, and neurocognitive deficits including perception, memory, and attention (2, 3). Worse negative symptoms and cognitive decline are associated with poor functional outcomes (2, 3). Although antipsychotic medication can help to manage some of the symptoms, they may not be able to address all the symptoms, and the patient still may relapse, or the medication may not have a lasting effect (4).

Typically, medication can help to manage the positive symptoms (hallucinations, delusions, confused thoughts or disorganized speech, trouble concentrating, or movement disorders), but not the negative symptoms (having a lack of pleasure, not talking much or showing much emotion, withdrawal or struggling to keep up with daily activities like bathing) (5, 6). Antipsychotic medications can also have unintended side effects, for example, weight gain and metabolic syndrome (which is a cluster of health conditions that can increase the risk of heart attack, stroke, and diabetes) (5, 6). As part of the clinical practice guidelines for the treatment of patients with schizophrenia, an initial assessment should include a physical health assessment, among assessing substance use, trauma history, risk of suicide and aggressive behavior and patient’s goals and preferences for treatment (7).

The clinical practice guidelines recommend both pharmacotherapy and psychotherapy as intervention strategies to manage schizophrenia (7). The guidelines emphasize that the first episode of psychosis be treated in a coordinated specialty care program (7). The guidelines do not specify exercise, but other literature indicates a value to integrating exercise early in the treatment of psychosis.

Physical activity and exercise are one treatment strategy used to manage side effects such as weight gain and metabolic syndrome as well as the primary cognitive, negative, and positive symptoms associated with psychosis (5, 6). In a study by Damme et al. (8) the group with high risk for psychosis had significantly lower VO2max than the healthy controls (8), however, this relationship may be confounded by body mass index (BMI) (9). Recent systematic reviews have looked at the effect of exercise and physical activity as a treatment strategy for people with psychosis (10–13). Exercise was found to be superior to usual care when it comes to improving total symptom severity as assessed by the Positive And Negative Symptom Severity scale (PANSS), cardiorespiratory function, depression, aerobic capacity, quality of life, and psychosocial stressors (10–12).

Exercise and physical activity may be one strategy to improve function and address psychotic symptoms in individuals in hospitals; however, the American Psychiatric Association highlighted a need for more research on patient-centered outcomes which includes physical health, determine the optimal setting of care and the optimal approaches to recovery (14). Therefore, the purpose of this systematic review and meta-analysis was to investigate the effectiveness of exercise and physical activity on psychotic symptoms in people with psychosis in a hospital setting when compared to usual care.

## Methods

We used the guidelines from the Preferred Reporting Items for Systematic Reviews and Meta-Analyses (PRISMA) and Cochrane collaboration guidelines for this systematic review and meta-analysis (15).

### Eligibility criteria

Studies were included in this review if the following criteria were met:

• Design: randomized controlled trial (RCT)
• Participants: Adults with psychosis in a hospital setting
• Intervention: Any form of exercise or physical activity
• Comparison: Usual care, other forms of exercise, self-directed care, treatment with another healthcare professional
• Outcomes: Psychosis symptom severity scores

Studies that were not written in English, were in a community setting or had no full text available were excluded from this systematic review explanation of excluded studies can be found in the Supplementary material. This review has been registered on PROSPERO: CRD42021224997.

### Information sources

A systematic electronic search of the literature was performed in November 2020, and updated in June 2022, in PubMed, Scopus, and PsychINFO with no date restrictions. The following key words were used to identify potentially relevant studies: “schizophrenia,” “bipolar,” “psychosis,” “exercise,” “exercise therapy,” “physical activity,” “randomized controlled trials,” “RCT.” In addition, we conducted a manual search of the reference lists of the included studies to identify any potential studies missed in the electronic search. The complete search strategy is summarized in Supplementary Figures 1–3.

### Study selection

The selection of individual studies involved three independent reviewers (CZ, RF, and PB) who performed the systematic electronic search of the databases. Two reviewers (CZ and PB) identified potentially relevant articles, removed duplicates, and then screened titles and abstracts. The full text of any study marked include or uncertain was obtained and the eligibility criteria were applied.

### Data collection process

Three independent researchers (CZ, RF, and PB) extracted the data from the eligible included studies. Data extraction included: study, author, year, sample characteristics, intervention, comparison groups, PANSS score (overall as well as subscales) and adverse or serious adverse events.

### Assessment of risk of bias in individual studies

Two independent review authors (CZ and PB) assessed the included RCTs for risk of bias (ROB). The ROB assessment was performed using the Cochrane Risk of Bias tool (16). The Cochrane ROB tool is based on 7 items, random sequence generation, allocation concealment, blinding of participants and personnel, blinding of outcome assessment, incomplete outcome data, selective reporting and other bias (16). The adequacy of each of the seven ROB domains was rated as “low,” “unclear,” or “high” risk according to criteria provided in the Cochrane Handbook for Systematic Reviews of Interventions (16). We summarized the assessment of ROB per study as Low ROB (if low ROB was judged for all the seven domains); as Unclear ROB (if unclear ROB was judged for one or more of the seven domains); and as High ROB (if high ROB was judged for one or more of the seven domains) (16).

### Quality of outcomes

The GRADE guidelines for systematic reviews were used to evaluate the quality of the psychosis symptom severity scores. The GRADE approach includes assessing ROB for study limitations, inconsistency, publication bias, imprecision, and indirectness (17–22) for the body of pooled trials. The rating of the quality of outcome across trials was carried out to summarize the extent of our confidence (high, moderate, low, or very low) that the estimates of the effect represent the truth (17–22).

### Synthesis of the results

We performed a random effects meta-analysis (Sidik-Jokman) of RCTs comparing exercise as a whole intervention or by different type of exercise (aerobic or yoga) to usual care in patients with psychosis on PANSS scores. We used STATA (Stata Statistical Software: Release 16, StataCorp LLC). We used the standardized mean difference (SMD) and reported 95% confidence intervals. We assessed small study effects with Harbord test (23) and we investigated the presence of publication bias with funnel plots (24). In the presence of publication bias we imputed the missing studies to account for publication bias in the meta-analysis. We compared the observed and the imputed studies by using the non-parametric “trim and fill” method. We used contour-enhanced funnel plot to assess if imputed studies fall in the region of statistical significance (25).

### Subgroup analysis and exploring heterogeneity

Our secondary outcome was to assess adverse events, safety outcomes were expressed as risk ratio. We quantified heterogeneity estimates of the between-study variance using the τ^2^ statistic (26). In the presence of substantial heterogeneity in the meta-analysis, we planned to perform separate univariate meta-regressions to quantify factors that may contribute to substantial heterogeneity. We prespecified the following factors: allocation concealment, year that the study was conducted and the time of follow-up.

## Results

### Study selection

**FIGURE 1:**
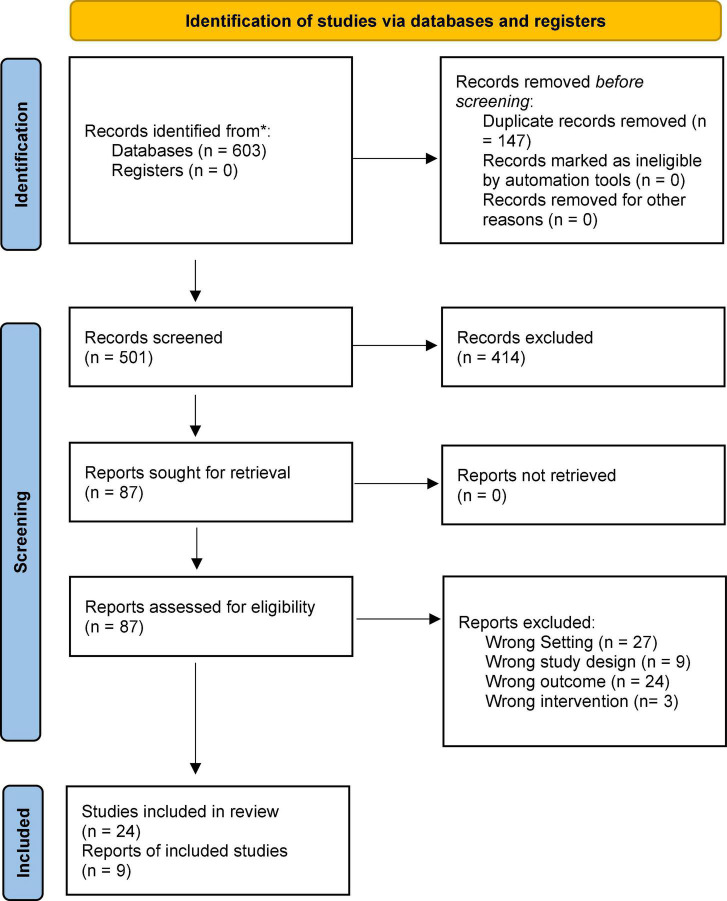
Flow chart of article screening and articles included in the review.

The initial search generated 603 articles. After removal of duplicates, and title and abstract screening 87 articles remained for full-text review. Of these, 15 met the eligibility criteria. Screening of reference lists resulted in identifying an additional 8 articles to be included. Our updated search yielded an additional 45 articles to review, one of which met the eligibility criteria. A total of 24 studies included in this systematic review. The study selection flow chart is presented in Figure 1.

### Study characteristics

The 24 eligible RCTs were conducted between 2006 and 2022 and included 1,426 participants. The study ranged from 10 to 160 participants. All trials were conducted on adults over the age of 18. A summary of the study characteristics is presented in Table 1.

**TABLE 1:** Summary of study characteristics.

Author	Country	Mean age intervention (SD)	Mean age control (SD)	N (males)	Primary outcome	Program length	Follow up	Drop out (n)	Adherence	Main inclusion criteria	Main exclusion criteria	Intervention details	Comparator details
Attux et al. (43)	Brazil	36.2 (9.9)	38.3 (10.7)	160 (96)	Weight loss	12 weeks	3 and 6 months	75	72.1%	Participants using antipsychotic medication, having schizophrenia, aged 18–65, and being clinically stable	Not clinically stable, in the presence of diabetes, history of an eating disorder, or drug or alcohol abuse	1 h weekly sessions to discuss dietary choices, lifestyle, physical activity and self-esteem	Usual care including antipsychotic medication and regular visits with a psychiatrist
Bang-Kittilsen et al. (28)	Norway	36.6 (14.3)	37.5 (13.8)	82 (50)	Neurocognition	12 weeks	4 months	25	NR	Participants with schizophrenia, aged 18–67 and able to speak and understand Scandinavian language	Pregnancy, chest pain, unstable angina, recent MI, uncontrollable cardiac arrhythmia, severe hypertension, comorbid diagnosis of mild mental retardation	8 min of warm-up, 4 min of treadmill running or walking at 85–95% max HR alternating with 3 min of 70% max HR	45 min of computerized interactive sport simulation
Beebe et al. (33)	United States	40–63 years	40–63 years	10 (8)	6-min walk distance	16 weeks	1 year	0	NR	Diagnosis of schizophrenia, medical clearance for moderate exercise	Significant cardiovascular, neuromuscular, endocrine or other disorders that would make it unsafe to exercise	10 min of warm-up, 30 min of walking, 10 min cool down	Usual care, offered exercise at the end of the study
Brobakken et al. (44)	Norway and United States	34 (10)	36 (12)	48 (28)	VO2 peak	12 weeks	12 weeks	14	83%	Diagnosis of schizophrenia, aged 18–65	Admitted to a psychiatric hospital, or any contraindications to exercise	Walk/run 4 × 4 min at 85–95% HR peak, 3 min of active rest at 70% HR peak, max strength training	Perform the intervention but independently
Curcic et al. (29)	Serbia	39.95 (9.51)	41.75 (9.45)	80 (42)	VO2 max	12 weeks	12 weeks	0	NR	Hospitalized for schizophrenia	No physical problems, no primary diagnosis of alcohol or substance abuse	Treadmill running for 45 min at 65–75% of max HR followed by 10 min of stretching	Continued with standard pharmacological therapy
Duraiswamy et al. (35)	India	32.53 (7.9)	31.30 (7.9)	61 (42)	PANSS	3 weeks	4 months	20	NR	Diagnosis of schizophrenia, aged 18–55	Severe physical ailments	Yoga, breathing and relaxation techniques	Brisk walking, jogging and exercises in standing and sitting
Heggelung et al. (41)	Norway	30.5 (8.7)	38.9 (11.4)	19 (13)	VO2 peak	8 weeks	8 weeks	6	85%	ICD-10 schizophrenia, schizotypal and delusional disorders. Stable on antipsychotic medication	Coronary artery disease, chronic obstructive pulmonary disease, unstable pharmacological treatment, not able to perform treadmill testing and exercise	The HIT group trained 4 × 4-min interval training on a treadmill at 85–95% HR peak interspersed with 3 min of active resting periods at a work load corresponding to 70% HR peak between each interval. Walking or running at 5% incline 3 × per week for 8 weeks	The CG group spent the same amount of time, 36 min three times per week, training to improve their ability in the computer game, Tetris
Ikai et al. (36)	Japan and Canada	54.8 (9.0)	51.5 (15.1)	49 (32)	Postural Sway	8 weeks	16 weeks	5	87%	Diagnosis of schizophrenia, 18 years and older	Incapable of giving consent, current substance or alcohol abuse	Yoga treatment including a warm-up, asana yoga, deep relaxation and breathing	Regular day-care. Provided yoga treatment at the end of the study
Ikai et al. (37)	Japan and Canada	53.5 (9.9)	48.2 (12.3)	50 (33)	Resilience	8 weeks	16 weeks	7	NR	Diagnosed with schizophrenia ore related psychotic disorder, 18 years or older, stable on medications	Patients incapable of providing consent, suffered from alcohol abuse or other psychiatric comorbidities	Yoga group received a weekly 1-h session of Hatha yoga therapy. Each session consisted of gentle yoga stretches and simple movements in coordination with breathing	Daycare sessions providing social skills and walking
Ikai et al. (38)	Japan and Canada	55.5 (11.4)	55.0 (15.8)	56 (36)	Postural Sway	12 weeks	18 weeks	7	NR	Inpatients with a diagnosis of psychiatric treatment, 20 years or older and capable of providing voluntary consent	Active alcohol abuse, and other psychiatric comorbidities	Chair yoga group received a 20-min chair yoga session, based on Hatha yoga, twice a week for 12 weeks.	Treatment as usual. Encouraged to spend time freely reading, walking, chatting
Kaltsatou et al. (27)	Greece	59.5 (19.6)	60.4 (8.6)	31 (15)	Functional Capacity	8 months	8 months	20	87%	Patients with schizophrenia, mini mental state score > 22, stable on medications	Significant cardiovascular, neuromuscular, physician deemed them too unwell to participate in exercise, or unable to provide informed consent	Traditional Greek dancing	Psychotherapy and was asked to continue their usual sedentary lifestyle
Kurebayashi et al. (47)	Japan	50.3 (14.0)	59.7 (13.0)	18	PANSS	8 weeks	8 weeks	4	NR	Chronic schizophrenia in the hospital, aged 18–50 with a PANSS negative score of at least 20 and 2 years since disease onset	Prominent neurological disease, cardiovascular clinical problem, IQ < 70	6 mg daily risperidone treatment and 30 min of aerobic training, 3 days per week, with supervision.	6 mg daily risperidone
Kwon et al. (42)	Korea	32 (9.2)	29.80 (6.07)	48 (15)	Weight and BMI	12 weeks	12 weeks	12	36.4%	Diagnosed with schizophrenia, aged 19–64	No history of hypomanic or psychotic states within 4 weeks, PANSS score > 70, severe medical disease, pregnant, hyperthyroidism, or hypothyroidism and any history of seizure or substance abuse	Cognitive behavioral therapy managing diet and exercise. Keeping a food diary, and talking with a dietitian. Exercise management through diary and education	Routine care with verbal recommendations about physical activity and diet
Li et al. (45)	China	51 (6.86)	50.97 (8.54)	61 (47)	Neurocognition	24 weeks	24 weeks	NR	NR	Diagnosis of schizophrenia, inpatient for 1 year, receiving antipsychotic medication	Having a serious disease such as cardiovascular, pulmonary disease, having uncorrected vision, having a condition that precludes exercising and exercising regularly within 6 months of the study	Baduanjin training 2 sessions 5 days per week for 40 min per day. 5 min warm-up, 30 min Baduanjin training and 5 min cool down	Brisk walking 5 days per week for 40 min per day
Loh et al. (56)	Malaysia	46 (14)	53 (11)	104 (74)	Quality of Life	3 months	3 months	4	NR	Adults with schizophrenia, age 18–65, receiving inpatient treatment	Bedridden patients, medical illness, and patient diagnosed with dementia	Group walking program and lifestyle. 3 times per week, 40 min of walking with a 5 min warm-up and cool down	Continuing usual care, doing their usual daily activities
Manjunath et al. (39)	India	31.7 (8.8)	31.1 (7.8)	88 (49)	CGI Illness Severity	2 weeks	6 weeks	28		Adults with psychosis	Any contraindications to exercise	Each yoga session lasted 1 h. After 2 weeks, patients were advised to practice the same for the next 4 weeks	Exercise training twice a week for 4 weeks
Methapatara et al. (34)	Thailand	43.16 (9.27)	37.59 (10.83)	64 (41)	Body Weight	12 weeks	12 weeks	NR	NR	Diagnosis of schizophrenia, aged 18–65, BMI of 23 kg/m2 or more, mild degree of illness, no plan for pregnancy in the next 6 months	Unstable medical condition, contraindications for exercise, cognitive impairment, participating in another clinical trial, pregnancy or breast feeding	Five 1-h sessions of group education on nutrition, exercise and using a pedometer to track steps	Usual care including antipsychotic medication
Oertel-Knöchel et al. (40)	Germany	44.6 (13.8)	38.3 (4.5)	17 (12)	Cognitive Performance	4 weeks	4 weeks	24	NR	Minimum of 5 years with psychotic disease, stable on medication	No comorbid psychotic diagnosis	Three weekly sessions each lasting 75 min over 4 weeks, of cognitive training and exercise	Three weekly sessions each lasting 75 min over 4 weeks of cognitive training and relaxation
Sailer et al. (30)	Germany	30.89 (11.41)	30.89 (11.41)	36 (25)	Attendance and Persistence	4 weeks	4 weeks	0	72.9%	Received inpatient or outpatient treatment for schizophrenia, and received treatment for at least 1 week	Severe psychiatric symptoms, and medical contraindications for exercise	30 min jogging sessions including warm-up and cool down	Education about physical activity
Scheewe et al. (5)	Netherlands	29.2 (7.2)	30.1 (7.7)	63 (46)	PANSS	6 months	6 months	24	NR	Diagnosed with schizophrenia, stable on antipsychotic medication	No significant medical problems, no primary diagnosis of alcohol or substance abuse	Muscle strength exercises, 6 exercises, 3 times per week, 10–15 repetitions with gradual increasing intensity	Occupational therapy for 1 h per week for 6 months
Shimada et al. (31)	Japan	50.14 (7.73)	49.75 (7.00)	41 (18)	Neurocognition	12 weeks	12 months	1	100%	Diagnosis of schizophrenia, aged 20–65	Diagnosis of mental retardation, alcohol/substance dependence, a known neurological disorder, possibility of difficulty participating in aerobic exercise	12 weeks, 2 sessions per week, 60-min sessions of aerobic exercise, at an intensity of 60–80% hear rate max	Usual treatment for schizophrenia and rehabilitation programs
Su et al. (32)	Taiwan	37.64 (8.23)	36.68 (8.33)	57 (20)	PANSS	3 months	3 months	13	76.6%	Diagnosed with schizophrenia, aged 20–60, had an IQ of greater than 70, and were on antipsychotic medication	Illness duration less than 1 year, significant neurological, metabolic or psychiatric condition, substance abuse issue, and current participation in other clinical trials	3 times per week for three months, aerobic exercise 55-69% HRmax, warm-up for 5 min, 30 min of aerobic exercise, 5-min cool down	Stretching program for 40 min at the same frequency as the intervention group
Varambally et al. (57)	India	32.8 (10)	30.6 (7.3)	151 (53)	PANSS	1 month	4 months	24	75%	Diagnosed with schizophrenia	Receiving antipsychotic medication without change in dose in 3 months, moderately symptomatic with a score of 3 of clinical global impression, and not receiving ECT	Yoga, breathing and relaxation techniques	Brisk walking, jogging and exercises in standing and sitting
Wang et al. (58)	Taiwan	38.3 (8.3)	38.7 (8.6)	32 (30)	PANSS	12 weeks	3 months	12	60%	Diagnosed with schizophrenia and were physically able to exercise	Schizophrenia for longer than 1 year, any neurological conditions, pregnant or breast feeding, substance abuse	Each AE session included 5 min of walking for a warm-up, followed by 30min of AE, then finally a 5-min cooldown period, i.e., 40 min in total.	The stretching and toning control program consisted of a 30-min recorded program of 14 exercise routines, including a 3-min warm-up, 25-min flexibility, toning and balance exercises designed to use all major muscle groups of the upper and lower extremities, and a 2-min cool down exercise performed to music

### Intervention and comparators

The studies included in this review were exercise and physical activity interventions, compared to usual care. There were a mix of exercise interventions including dancing (27), running (28–32), walking (33, 34), yoga (35–39), strength training (5), high intensity interval training (28, 40, 41), and lifestyle modifications, by increasing daily walking and mobility (42, 43). Some interventions included a combination of both strength and aerobic exercise training (44, 45).

The comparator groups were usual care or an active intervention. The usual care most commonly included antipsychotic medications and being followed by a psychiatrist. We found lack of reporting from majority of the included studies on which antipsychotic medication was prescribed. The information about the medication use that was available is described in Table 1. Active interventions in the comparator group included walking or stretching (28, 32, 35, 39, 45). Some of the comparator groups were related to education about physical activity (30) or spending time with an occupational therapist (5, 42). More information about the comparator characteristics is presented in Table 1.

### Excluded studies

Of the 90 articles that were deemed relevant for full text review, 24 were included in this systematic review and meta-analysis. The remaining articles were excluding for the following reasons:

- Wrong setting, was not in a hospital setting (*n* = 27)
- Wrong outcome, did not included PANSS as part of their outcome assessments (*n* = 24)
- Wrong study design, was not an RCT (*n* = 9)
- Wrong intervention, not exercise or physical activity (*n* = 3)

The reasons of the excluded studies are presented in Supplementary Web Appendix.

### Risk of bias

Blinding of the participants (performance bias) was rated as high in 18 out of 24 trials. Random sequence generation was rated as low risk of bias in 15 out of 24 trials. We found no indication of publication bias see funnel plot in Supplementary Web Appendix Figure 4, and Supplementary Tables 3, 4.

### Quality assessment per outcome

We found very low to moderate quality certainty for the pooled psychosis symptom severity scores (Table 2). Most of the studies were downgraded due to high risk of bias, from lack of blinding and incomplete outcome data.

**TABLE 2:** Certainty of evidence per outcome.

Outcomes	Comparison	No of participants (Studies)	Quality of evidence (GRADE)	Standardized mean difference (95% CI)
PANSS total	Any exercise vs. usual care	376 (9)	⊕⊕⊕○ MODERATE (1)	−0.29, (−0.52 to −0.07)
	Yoga vs. usual care	155 (3)	⊕⊕○○ LOW (1, 2)	−0.06, (−0.38 to 0.26)
	Aerobic exercise vs. usual care	221 (6)	⊕⊕⊕○ MODERATE (1)	−0.25, (−0.52 to 0.03)
PANSS positive	Any exercise vs. usual care	376 (9)	⊕⊕⊕○ MODERATE (1)	−0.17, (−0.38 to 0.04)
	Yoga vs. usual care	155 (3)	⊕⊕○○ LOW (1, 2)	−0.09, (−0.42 to 0.23)
	Aerobic exercise vs. usual care	170 (4)	⊕⊕○○ LOW (1, 2)	−0.23, (−0.53 to 0.07)
PANSS negative	Any exercise vs. usual care	357 (8)	⊕⊕⊕○ MODERATE (1)	−0.30, (−0.52 to −0.01)
	Yoga vs. usual care	155 (3)	⊕⊕○○ LOW (1, 2)	−0.20, (−0.53 to 0.13)
	Aerobic exercise vs. usual care	202 (5)	⊕⊕○○ LOW (1, 2)	−0.38, (−0.65 to −0.10)
PANSS general	Any exercise vs. usual care	325 (7)	⊕⊕⊕○ MODERATE (1)	−0.16, (−0.39 to 0.06)
	Yoga vs. usual care	155 (3)	⊕⊕○○ LOW (1, 2)	−0.10, (−0.41 to 0.22)
	Aerobic exercise vs. usual care	221 (6)	⊕⊕○○ LOW (1, 2)	−0.42, (−0.71 to −0.13)

GRADE Working group grades of Evidence.

High quality: Further research is very unlikely to change our confidence in the estimate effect.

Moderate quality: Further research is likely to have an important impact on our confidence in the estimate of effect and may change the estimate.

Low quality: Further research is very likely to have an important impact on our confidence in the estimate of effect and is likely to change the estimate.

Very low quality: We are very uncertain about the estimate.

^1^downgraded due to risk of bias.

^2^downgraded due to imprecision.

### Participants

A total of 1,426 participants were included in this review. The average age across all the studies was 39.5 years. There were 825 males included in the study, which was 58% of the participants. More information about the study demographics is included in Table 1.

### Outcomes

Five of studies used the overall PANSS as the primary outcome (5, 32, 35, 46, 47), and two used the PANSS subscale as the primary outcome. Three of the studies assessed neurocognition as the primary outcome (28, 31, 45). Three of the studies assessed weight loss as a primary outcome (34, 42, 43). Four studies assessed performance through either the 6-min walk distance (33) or VO2 max/peak (29, 41, 44). A summary of the included articles’ primary outcomes is presented in Table 1.

### Timeframe

The timeframe for most of the studies (9/24 studies) for the exercise intervention was 12 weeks [range (min-max) 3–34.4 weeks]. A summary of the program length and follow up time frames are presented in Table 1.

### Drop out and adherence

Drop out was not reported in two of the studies. Three of the studies had no participants drop out (29, 30, 46). One trial reported high attrition and had the highest number of participants to drop out (*n* = 75) across all trials which was 47% of the participants that were randomized (43).

Adherence was not well reported in the studies. Some studies required participants to maintain a certain adherence (e.g., 75 or 80% of sessions) or the participants were excluded from the study. A summary of all the studies that reported the rate of drop out and adherence is presented in Table 1.

#### Meta-analysis of the exercise as a whole intervention

**FIGURE 2:**
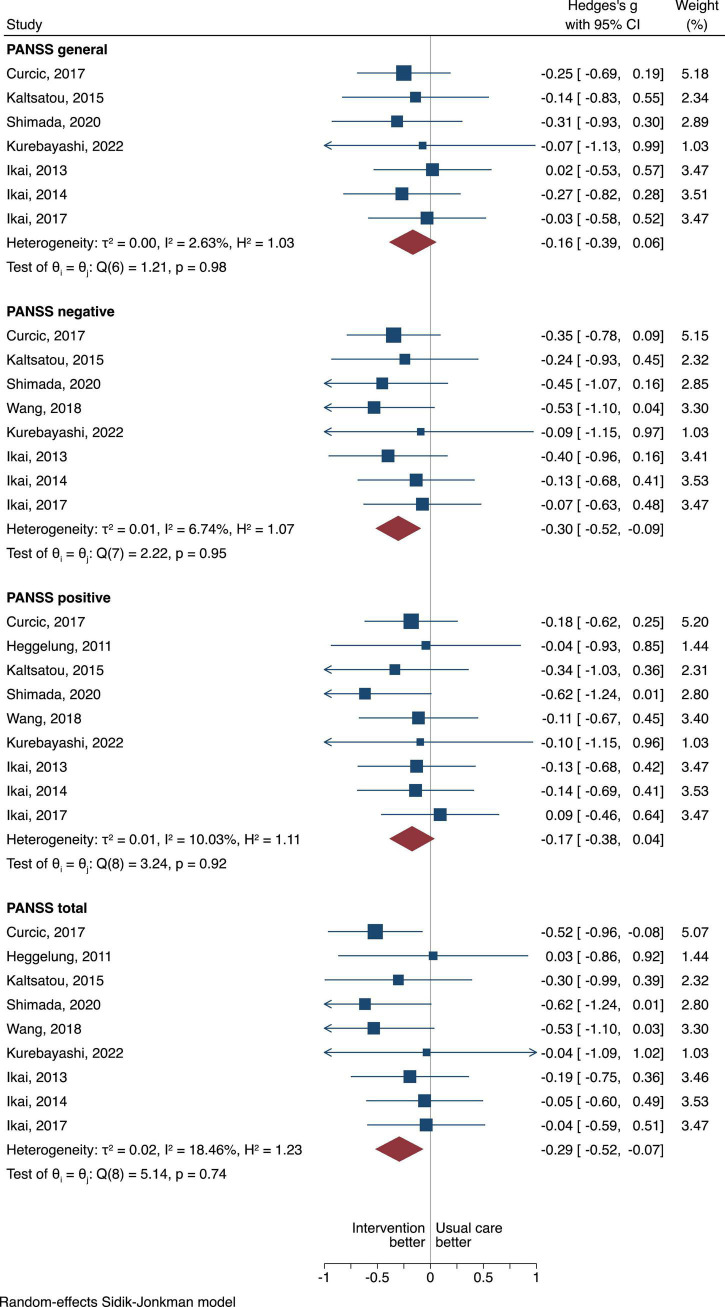
The between group difference of the exercise group compared to usual care is expressed as standardized mean difference (SMD). A negative difference means that the treatment resulted in a lower PANSS scores compared to the usual care, which is favorable.

Nine studies were included in the meta-analysis of the PANSS outcome. Figure 2 shows the between group differences of all the exercise modalities as whole intervention compared to usual care.

#### Overall positive and negative symptom severity scale scores (primary outcome of interest)

Exercise as an intervention in hospital setting had more pronounced effects when compared to usual care in PANSS overall scores with a pooled SMD of −0.29, 95% CI = −0.52 to −0.07, τ^2^ = 0.02, *n* = 9, moderate certainty of evidence (Figure 2).

#### Negative symptom severity scale scores

When looking at the negative PANSS scores, exercise compared to usual care displayed statistical superiority with a pooled SMD of −0.30, 95% CI = −0.52 to −0.09, τ^2^ = 0.01, *n* = 8, moderate certainty of evidence (Figure 2).

#### Positive symptom severity scale scores

For the positive PANSS scores, although exercise was favored when compared to usual care the difference was not statistically different (SMD of −0.17, 95% CI = −0.38 to 0.04, τ^2^ = 0.01, *n* = 9), moderate certainty of evidence (Figure 2).

#### General positive and negative symptom severity scale scores

For the general PANSS scores, although exercise was favored when compared to usual care the difference was not statistically different (SMD of −0.16, 95% CI = −0.39 to 0.06, τ^2^ = 0, *n* = 7), moderate certainty of evidence (Figure 2).

#### Meta-analysis by different type of exercise modality

**FIGURE 3:**
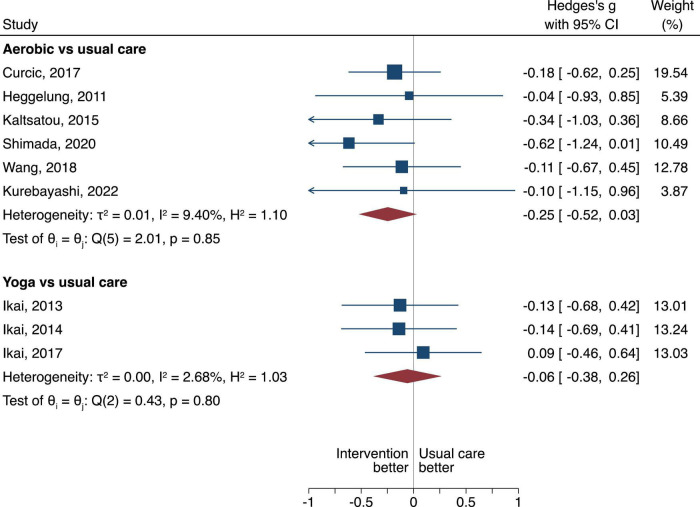
The between group difference of the aerobic or yoga compared to usual care is expressed as standardized mean difference (SMD). A negative difference means that the treatment resulted in a lower PANSS overall score compared to the usual care, which is favorable.

**FIGURE 4:**
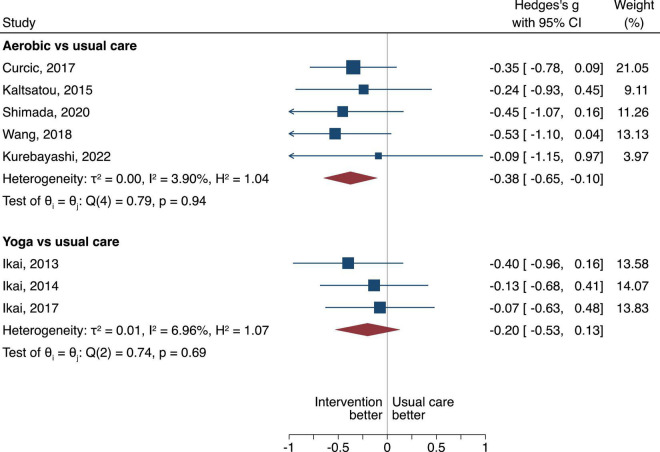
Negative PANSS scores in random-effects Sidik-Jonkman model. The between group difference of the aerobic or yoga compared to usual care is expressed as standardized mean difference (SMD). A negative difference means that the treatment resulted in a lower PANSS negative score compared to the usual care, which is favorable.

**FIGURE 5:**
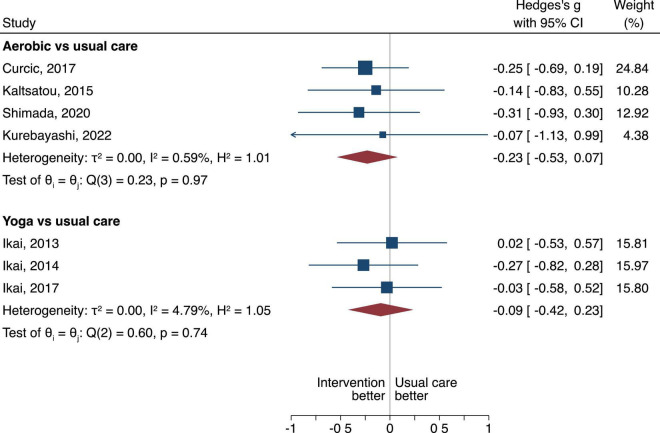
Positive PANSS scores in random-effects Sidik-Jonkman model. The between group difference of the aerobic or yoga compared to usual care is expressed as standardized mean difference (SMD). A negative difference means that the treatment resulted in a lower PANSS positive score compared to the usual care, which is favorable.

**FIGURE 6:**
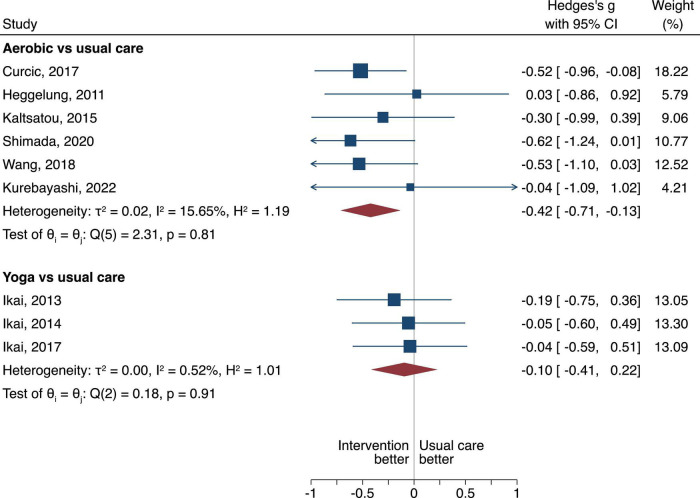
General PANSS scores in random-effects Sidik-Jonkman model. The between group difference of the aerobic or yoga compared to usual care is expressed as standardized mean difference (SMD). A negative difference means that the treatment resulted in a lower PANSS general score compared to the usual care, which is favorable.

To get a better understanding of which type of exercise affects psychotic symptoms, a comparison for the PANSS scores was made between aerobic and usual care, and yoga vs. usual care (Figures 3–6).

#### Overall positive and negative symptom severity scale scores

Aerobic had more pronounced effects when compared to usual care, however, the difference was not statistically significant (SMD of −0.25, 95% CI = −0.52 to 0.03, τ^2^ = 0.01, *n* = 6, low certainty of evidence, Figure 3). Yoga had no difference when compared to usual care (SMD of −0.06, 95% CI = −0.38 to 0.26, τ^2^ = 0, *n* = 3, very low certainty of evidence, Figure 3).

#### Negative symptom severity scale scores

When looking at the negative PANSS Score, aerobic when compared to usual care displayed statistical superiority with a pooled SMD of −0.38, 95% CI −0.65 to −0.10, τ^2^ = 0, *n* = 5, low certainty of evidence (Figure 4). Yoga when compared to usual care had no difference (SMD of −0.20, 95% CI = −0.53 to 0.13), τ^2^ = 0.01, very low certainty of evidence (Figure 4).

#### Positive symptom severity scale scores

For the positive PANSS scores, we found no difference either for aerobic or yoga when compared to usual care (SMD of −0.23, 95% CI = −0.53 to 0.07, τ^2^ = 0, *n* = 4), very low certainty of evidence for the aerobic; and SMD of −0.09, 95% CI = −0.42 to 0.23, τ^2^ = 0, *n* = 3, very low certainty of evidence for the yoga intervention (Figure 5).

#### General positive and negative symptom severity scale scores

When looking at the general PANSS scores, aerobic when compared to usual care displayed statistical superiority with a pooled SMD of −0.42, 95% CI −0.71 to −0.13, τ^2^ = 0.02, *n* = 6, very low certainty of evidence (Figure 6). We found no difference when yoga compared to usual care (SMD of −0.10, 95% CI = −0.41 to 0.22), τ^2^ = 0, low certainty of evidence (Figure 6).

### Adverse events

**FIGURE 7:**
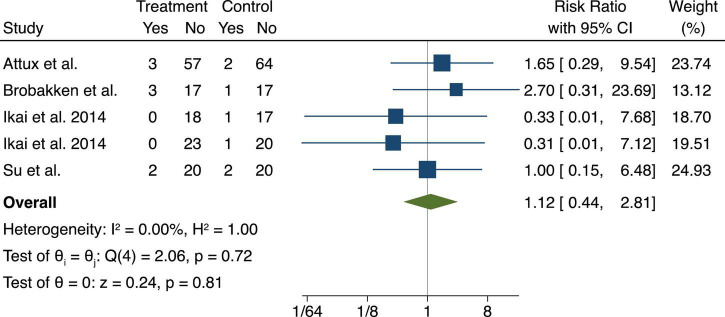
Serious adverse events (relapsing or hospitalization) displayed in a fixed-effects Mantel-Haenszel model. The between group difference of exercise modalities compared to usual care is expressed as Risk Ratio (RR). A risk ratio below 1 means that the exercise intervention had lower risk while above 1 indicates higher risk when compared to the usual care.

Adverse events were reported in 11 of the included studies (28, 30–32, 35, 37–39, 42–44). Most commonly the adverse event was a relapse of psychiatric symptoms, however, one study reported death, but the investigators reported that it was unrelated to the study protocols (44). One study reported on a somatic hospitalization (44). In our meta-analysis of serious adverse events, we found no difference on relapsing of psychiatric symptoms or somatic hospitalizations when we compared exercise to usual care with a pooled Risk Ratio (RR), 1.12 95% CI 0.44–2.81 (Figure 7).

## Discussion

### Main findings

This systematic review and meta-analysis found a benefit to exercise training compared to usual care in schizophrenic adults in a hospital setting. Our meta-analysis showed more pronounced treatment effects in the PANSS overall score in the exercise intervention groups compared to the usual care. We further analyzed whether a certain type of exercise resulted in improvements in psychiatric symptoms. The ROB in the studies was mostly high, due to lack of blinding. ROB assessments for any study of exercise programs would have to be downgraded since it is impossible to blind the instructors or participants in exercise programs. The quality of the outcomes was low to moderate due to a high ROB, and imprecision. Larger sample sizes and more studies evaluating the effect of exercise in a hospital setting would need to confirm the effect of exercise for managing psychotic symptoms.

### Previous evidence

Several other systematic reviews and meta-analyses have been conducted on the effects of exercise for people with psychosis. A Cochrane review published in 2013 of 39 trials and 1,356 participants compared exercise with no treatment or control intervention for the primary outcome of depression (48). They found that there was a moderate clinical effect, and the results were statistically significant (48). In another Cochrane review looking at exercise therapy for schizophrenia, only three studies were included in this review but also found exercise significantly improved negative symptoms related to schizophrenia (49). A more recent meta-analysis of 17 RCT’s also found a benefit of exercise on negative symptoms for people with schizophrenia (50). Our study was unique in pooling all exercise as well as analyzing the effects of two different types of exercise (aerobic and yoga) compared to usual care on the effects of psychotic symptoms. We found improvements in psychotic symptoms in the intervention group compared to usual control, which is in agreement with the Cochrane reviews. We also found that aerobic exercise improved negative and general symptoms when compared to usual care, but yoga had no effect. One Cochrane review published in 2015 included 8 studies found that outcomes related to mental state, social functioning, and quality of life favored yoga compared to usual care (51). Our study could not confirm these findings because we did not evaluate these outcomes.

In another meta-analysis looking at mindful exercise compared to usual care, 7 studies were included and there was found to be a significant improvement in psychiatric symptoms and memory for the group that participated in the mindful exercise (52). Mindful exercise emphasizes deep, slow and relaxed breathing, and internal concentration to reduce stress by minimizing external stressors and improving mental focus (53). Although mindful exercise was not part of the interventions included in the current study, there could be an indirect effect of aerobic exercise and yoga on mindfulness, further supporting the benefits of exercise for people with psychosis.

### Clinical and research implications

This meta-analysis provided unique insight into the benefits of exercise in people with schizophrenia in a hospital setting. The study by Curcic et al. (29), showed the greatest effect in the meta-analysis (29). The intervention was a treadmill running program for 45 min at 65–75% of max HR, and the control group continued with standard care. In Curcic et al. (29), standard of care was pharmacotherapy (29). It is known that exercise at a higher intensity can help with symptoms like depression and anxiety, which may also contribute to the positive and negative symptoms experienced in people with schizophrenia (29). However, not all the running exercise studies included in this systematic review showed statistically or clinically significant improvements in the PANSS score compared to the control. This is likely because in some of the studies the control group was active control, and in one case the control group received the same intervention just without supervision (44).

One challenge to implementing an exercise program in a hospital setting is having access to personnel and resources. A study intervention allows for better implementation of an exercise program, because study personnel can run the program. Within the hospital setting, and outside of a research intervention, the program will rely on utilizing staff that are currently available. It is most feasible to rely on occupational and physical therapists to deliver exercise interventions. In the studies assessed in this review, one study intervention used occupational therapists to help with delivery (5), while none of the studies discussed the use of a physical therapist. Physical therapists are trained to provide exercise therapy safely and effectively. Recognizing the benefits that exercise has on psychiatric symptoms, exercise should be considered an essential part of treatment, and physical and occupational therapists should be used to administer the programs.

It is unclear how well received these programs were by the participants, as many studies did not report on adherence. However, in the studies that did report adherence the adherence ranged quite a bit with the lowest reported adherence as 36% adherence (42) and the highest reported adherence as 87% (36). In the study by Kwon et al. (42) with 36% adherence, participants had to attended 80% of the exercise sessions (42). The study by Ikai et al. (36) attribute their higher adherence to a flexible exercise schedule, and not requiring the participants to engage in exercise on their own (36). Based on these numbers it is not completely clear how well an exercise intervention is implemented into a hospital setting, but there seems to be some promising results, that even with a low adherence there may be benefit to psychiatric symptoms.

There were some adverse events reported, with 5 studies reporting serious adverse events, but suggested it was unrelated to the study protocol. For many of the studies contraindications to exercise was an exclusion criterion. That being said, due to increased movement, there is an increased risk of falling (54). It would be beneficial for all patients to undergo an exercise readiness screening, to assess balance, strength, and endurance prior to beginning the exercise program. As well, the exercise program should be modified for the individual, to provide maximal benefits and reduce the risk of exercise-related injuries. Most participants had to be stable on their medications to be eligible to participate in the study. However, it’s feasible to think there may be a risk to other participants or the instructor. It would be advised to follow hospital protocols to reduce the risk of behavior related adverse events. As well, relapse was another adverse event reported by five of the studies. It is not uncommon for patients with psychiatric symptoms to relapse, and it’s possible that with more exercise symptoms can be better managed and there may be less relapse events.

Finally, there were some notable gaps in the literature reducing the generalizability of these results. All the participants were between the ages of 18 and 65. Unfortunately this restricts the generalizability to older adults. Future studies should evaluate whether exercise interventions are feasible in older adults within a hospital setting. It may be that the mental illness is further progressed, and their mobility is further declined requiring a different approach than adults. There is also likely a fear of older adults with psychotic symptoms and likely other comorbidities like dementia participating in exercise interventions. However, there is evidence to suggest that exercise can help manage the behavioral and psychological symptoms of dementia (55). Trialing an exercise program in a hospital setting may be safer with extra personnel available.

### Strengths and limitations

Overall, this study demonstrated both strengths and limitations. The strength of this study was that we were able to conduct a meta-analysis in 9 trials, with the exercise intervention as whole and as two different exercise forms including yoga, and aerobic with no or very low statistical heterogeneity. The study effects and the overall effects of the meta-analysis demonstrated that the exercise intervention had a more pronounced effect, but the clinical benefit was uncertain. We also assessed the risk of bias to provide insight into the internal validity of the included studies. Although we believe we conducted a thorough literature search, where three databases, gray literature and reference lists were used, it’s possible some trials may have been missed. Also, the results in this study demonstrate a low to moderate certainty, and need to be interpreted with caution, until more studies can corroborate our findings. Pharmacotherapy was used as part of the treatment which can have a strong effect on mood, psychotic symptoms as well as physiological side effects which could make the clinical benefits and effectiveness of the intervention less pronounced, suggesting a potentially greater effect of exercise than is seen in our meta-analysis.

## Conclusion

Aerobic intervention seems to be effective in reducing negative and general symptoms in patients with schizophrenia. However, we are uncertain if the effects of aerobic interventions were clinically important. We found no benefit of yoga when compared to standard of care. Additionally, the safety of exercise interventions needs to be monitored as less than half of the included trials reported adverse events. We found no increased risk on symptom relapsing or rehospitalizations for exercise interventions. Additional studies need to be conducted to corroborate these findings.

## Data availability statement

The original contributions presented in this study are included in this article/Supplementary material, further inquiries can be directed to the corresponding author.

## Author contributions

CZ, DS, and MD contributed to the idea of the study. CZ, PB, and RF identified the articles and extracted the data. CZ, JM, and PB contributed to the analysis of the study. All authors contributed to the writing and editing of the manuscript.
