# Supplementary material for: The efficacy and safety of exercise and physical activity on psychosis: A systematic review and meta-analysis
Source: Front Psychiatry. 2022 Aug 16;13:807140. doi: 10.3389/fpsyt.2022.807140 (PMC9425642; doi:10.3389/fpsyt.2022.807140)
Supplement: Supplementary file 1 [file Data_Sheet_1.docx]

**The efficacy of exercise and physical activity on psychosis: A systematic review and meta-analysis**

Christina Ziebart^a,b^, Pavlos Bobos^f,g^, Joy C. MacDermid^a,b^, Daniel J. Sobczak^e^, and Michele Doering^e^

**WEB APPENDIX**

Contents

[I. Excluded Studies 1](#_Toc108615277)

[Reasons for exclusion 1](#_Toc108615278)

[II. Supplementary Tables 7](#_Toc108615279)

[Supplementary Table 1. Scopus search strategy 7](#_Toc108615280)

[Supplementary Table 2. Database findings 7](#_Toc108615281)

[Supplementary Table 3. Summary of review's authors judgments on risk of bias 9](#_Toc108615282)

[Supplementary Table 4: Summary of Risk of bias assessment 98](#_Toc108615283)

[III. Supplementary Figures 99](#_Toc108615284)

[FIGURE S1. Medline Search Strategy 99](#_Toc108615285)

[FIGURE S2. Scopus search strategy 99](#_Toc108615286)

[FIGURE S3. PsychInfo (Ovid) search strategy 99](#_Toc108615287)

[FIGURE S4: Funnel plot showing publication bias 100](#_Toc108615288)

# I. Excluded Studies

## Reasons for exclusion

Of the 85 articles that were deemed relevant for full text review, 62 were excluded in this systematic review. The articles were excluding for the following reasons:

1. Wrong setting, was not in an inpatient or outpatient setting (n= 27, Ho et al., 2016; Lin et al., 2015; Marzolini et al., 2009; Andrade e Silva et al., 2015; Andrews et al., 2016; Daumit et al., 2013; Horan et al., 2018; Holt et al., 2018; Holt et al., 2019; Stiekema et al., 2018; Jakobsen et al., 2017; Winstock et al., 2016; Ho et al., 2014; Lovell et al., 2014; Forsberg et al., 2008; Yoarborough et al., 2013; Priebe et al., 2016; Bonfioli et al., 2018; Van Citers et al., 2010; Looijmans et al., 2017; Kang et al., 2016; Williams et al., 2019; Krinar et al., 2005; Gaughran et al., 2017; Ryu et al., 2020; Romain et al., 2019; Beebe et al., 2005)
2. Wrong outcome, did not included PANSS as part of their outcome assessments (n= 24, Beebe et al., 2010; Beebe et al., 2011; Bhatia et al., 2017; Brobakken et al., 2020; Cheng et al., 2020; Daumit et al., 2011; Duncan et al., 2016; Kilbourne et al., 2017; Kimhy et al., 2014; Kimhy et al., 2016; Leutwyler et al., 2014; Mazyarkin et al., 2019; Ng et al., 2007; Scheewe et al., 2013; Vancampfort et al., 2011; Attux et al., 2011; Kaltsatou et al., 2015; Woodward et al., 2020; Armstrong et al., 2016; Falkai et al., 2013; Shimada et al., 2019; Visceglia et al., 2011; Woodward et al., 2018; Andersen et al., 2020)
3. Wrong study design, was not an RCT (n= 9, Acil et al., 2008; Paikkatt et al., 2012; Paikkatt et al., 2015; Behere et al., 2011; Wolff et al., 2011; Lin et al., 2017; Beebe et al., 2011; Bhatia et al., 2014;Khonsari, 2022.)
4. Wrong intervention, not exercise or physical activity (n= 3; Gholipour et al., 2012; Looijmans et al., 2014; Wu et al., 2007)

References:

1. Acil AA, Dogan S, Dogan O. The effects of physical exercises to mental state and quality of life in patients with schizophrenia. Journal of psychiatric and mental health nursing. 2008 Dec;15(10):808-15.
2. Andersen E, Bang-Kittilsen G, Bigseth TT, Egeland J, Holmen TL, Martinsen EW, Stensrud T, Engh JA. Effect of high-intensity interval training on cardiorespiratory fitness, physical activity and body composition in people with schizophrenia: a randomized controlled trial. BMC psychiatry. 2020 Dec;20(1):1-2.
3. Andrade e Silva B, Cassilhas RC, Attux C, Cordeiro Q, Gadelha AL, Telles BA, Bressan RA, Ferreira FN, Rodstein PH, Daltio CS, Tufik S. A 20-week program of resistance or concurrent exercise improves symptoms of schizophrenia: results of a blind, randomized controlled trial. Brazilian Journal of Psychiatry. 2015 Dec;37(4):271-9.
4. Andrews M, Baker AL, Halpin SA, Lewin TJ, Richmond R, Kay-Lambkin FJ, Filia SL, Castle D, Williams JM, Clark V, Callister R. Early therapeutic alliance, treatment retention, and 12-month outcomes in a healthy lifestyles intervention for people with psychotic disorders. The Journal of Nervous and Mental Disease. 2016 Dec 1;204(12):894-902.
5. Armstrong HF, Bartels MN, Paslavski O, Cain D, Shoval HA, Ballon JS, Khan S, Sloan RP, Kimhy D. The impact of aerobic exercise training on cardiopulmonary functioning in individuals with schizophrenia. Schizophrenia research. 2016 May;173(1-2):116.
6. Attux C, Martini LC, Araújo CM, Roma AM, Reis AF, Bressan RA. The effectiveness of a non-pharmacological intervention for weight gain management in severe mental disorders: results from a national multicentric study. Brazilian Journal of Psychiatry. 2011 Jun;33(2):117-21.
7. Beebe LH, Smith K, Burk R, Dessieux O, Velligan D, Tavakoli A, Tennison C. Effect of a motivational group intervention on exercise self-efficacy and outcome expectations for exercise in schizophrenia spectrum disorders. Journal of the American Psychiatric Nurses Association. 2010 Apr.
8. Beebe LH, Smith K, Burk R, McIntyre K, Dessieux O, Tavakoli A, Tennison C, Velligan D. Effect of a motivational intervention on exercise behavior in persons with schizophrenia spectrum disorders. Community mental health journal. 2011 Dec 1;47(6):628-36.
9. Beebe LH, Smith K, Burk R, McIntyre K, Dessieux O, Tavakoli A, Velligan D. Motivational intervention increases exercise in schizophrenia and co-occurring substance use disorders. Schizophr. Res. (2011), doi:10.1016/j.schres.2011.12.008
10. Beebe LH, Tian L, Morris N, Goodwin A, Allen SS, Kuldau J. Effects of exercise on mental and physical health parameters of persons with schizophrenia. Issues in mental health nursing. 2005 Jan 1;26(6):661-76.
11. Behere RV, Arasappa R, Jagannathan A, Varambally S, Venkatasubramanian G, Thirthalli J, Subbakrishna DK, Nagendra HR, Gangadhar BN. Effect of yoga therapy on facial emotion recognition deficits, symptoms and functioning in patients with schizophrenia. Acta Psychiatrica Scandinavica. 2011 Feb;123(2):147-53.
12. Bhatia T, Mazumdar S, Mishra NN, Gur RE, Gur RC, Nimgaonkar VL, Deshpande SN. Protocol to evaluate the impact of yoga supplementation on cognitive function in schizophrenia: a randomised controlled trial. Acta neuropsychiatrica. 2014 Oct;26(5):280.
13. Bhatia T, Mazumdar S, Wood J, He F, Gur RE, Gur RC, Nimgaonkar VL, Deshpande SN. A randomised controlled trial of adjunctive yoga and adjunctive physical exercise training for cognitive dysfunction in schizophrenia. Acta neuropsychiatrica. 2017 Apr;29(2):102-14.
14. Bonfioli E, Mazzi MA, Berti L, Burti L. Physical health promotion in patients with functional psychoses receiving community psychiatric services: Results of the PHYSICO-DSM-VR study. Schizophrenia research. 2018 Mar 1;193:406-11.
15. Brobakken MF, Nygård M, Güzey IC, Morken G, Reitan SK, Heggelund J, Vedul‐Kjelsaas E, Wang E. One‐year aerobic interval training in outpatients with schizophrenia: A randomized controlled trial. Scandinavian Journal of Medicine & Science in Sports. 2020 Dec;30(12):2420-36.
16. Cheng SL, Sun HF, Yeh ML. Effects of an 8-week aerobic dance program on health-related fitness in patients with schizophrenia. journal of nursing research. 2017 Dec 1;25(6):429-35.
17. Daumit GL, Dalcin AT, Jerome GJ, Young DR, Charleston J, Crum RM, Anthony C, Hayes JH, McCarron PB, Khaykin E, Appel LJ. A behavioral weight-loss intervention for persons with serious mental illness in psychiatric rehabilitation centers. International journal of obesity. 2011 Aug;35(8):1114-23.
18. Daumit GL, Dickerson FB, Wang NY, Dalcin A, Jerome GJ, Anderson CA, Young DR, Frick KD, Yu A, Gennusa III JV, Oefinger M. A behavioral weight-loss intervention in persons with serious mental illness. New England Journal of Medicine. 2013 Apr 25;368(17):1594-602.
19. Duncan MJ, Faulkner G, Remington G, Arbour-Nicitopoulos K. Characterizing the affective responses to an acute bout of moderate-intensity exercise among outpatients with schizophrenia. Psychiatry Research. 2016 Mar 30;237:264-70.
20. Falkai P, Malchow B, Wobrock T, Gruber O, Schmitt A, Honer WG, Pajonk FG, Sun F, Cannon TD. The effect of aerobic exercise on cortical architecture in patients with chronic schizophrenia: a randomized controlled MRI study. European archives of psychiatry and clinical neuroscience. 2013 Sep 1;263(6):469-73.
21. Forsberg KA, Björkman T, Sandman PO, Sandlund M. Physical health—a cluster randomized controlled lifestyle intervention among persons with a psychiatric disability and their staff. Nordic Journal of Psychiatry. 2008 Jan 1;62(6):486-95.
22. Gaughran F, Stahl D, Ismail K, Greenwood K, Atakan Z, Gardner-Sood P, Stubbs B, Hopkins D, Patel A, Lally J, Lowe P. Randomised control trial of the effectiveness of an integrated psychosocial health promotion intervention aimed at improving health and reducing substance use in established psychosis (IMPaCT). BMC psychiatry. 2017 Dec 1;17(1):413.
23. Gholipour A, Abolghasemi SH, Gholinia K, Taheri S. Token reinforcement therapeutic approach is more effective than exercise for controlling negative symptoms of schizophrenic patients: a randomized controlled trial. International Journal of Preventive Medicine. 2012 Jul;3(7):466.
24. Ho RT, Fong TC, Wan AH, Au-Yeung FS, Wong CP, Ng WY, Cheung IK, Lo PH, Ng SM, Chan CL, Chen EY. A randomized controlled trial on the psychophysiological effects of physical exercise and Tai-chi in patients with chronic schizophrenia. Schizophrenia research. 2016 Mar 1;171(1-3):42-9.
25. Ho RT, Wan AH, Au-Yeung FS, Lo PH, Siu PJ, Wong CP, Ng WY, Cheung IK, Ng SM, Chan CL, Chen EY. The psychophysiological effects of Tai-chi and exercise in residential Schizophrenic patients: a 3-arm randomized controlled trial. BMC complementary and alternative medicine. 2014 Dec 1;14(1):364.
26. Holt RI, Gossage-Worrall R, Hind D, Bradburn MJ, McCrone P, Morris T, Edwardson C, Barnard K, Carey ME, Davies MJ, Dickens CM. Structured lifestyle education for people with schizophrenia, schizoaffective disorder and first-episode psychosis (STEPWISE): randomised controlled trial. The British Journal of Psychiatry. 2019 Feb;214(2):63-73.
27. Holt RI, Hind D, Gossage-Worrall R, Bradburn MJ, Saxon D, McCrone P, Morris TA, Etherington A, Shiers D, Barnard K, Swaby L. Structured lifestyle education to support weight loss for people with schizophrenia, schizoaffective disorder and first episode psychosis: the STEPWISE RCT. Health technology assessment (Winchester, England). 2018 Nov;22(65):1.
28. Horan WP, Dolinsky M, Lee J, Kern RS, Hellemann G, Sugar CA, Glynn SM, Green MF. Social cognitive skills training for psychosis with community-based training exercises: a randomized controlled trial. Schizophrenia bulletin. 2018 Oct 17;44(6):1254-66.
29. Jakobsen AS, Speyer H, Nørgaard HC, Karlsen M, Birk M, Hjorthøj C, Mors O, Krogh J, Gluud C, Pisinger C, Nordentoft M. Effect of lifestyle coaching versus care coordination versus treatment as usual in people with severe mental illness and overweight: Two-years follow-up of the randomized CHANGE trial. PLoS One. 2017 Oct 6;12(10):e0185881.
30. Kaltsatou A, Kouidi E, Fountoulakis K, Sipka C, Theochari V, Kandylis D, Deligiannis A. Effects of exercise training with traditional dancing on functional capacity and quality of life in patients with schizophrenia: a randomized controlled study. Clinical rehabilitation. 2015 Sep;29(9):882-91.
31. Kang R, Wu Y, Li Z, Jiang J, Gao Q, Yu Y, Gao K, Yan Y, He Y. Effect of community-based social skills training and tai-chi exercise on outcomes in patients with chronic schizophrenia: a randomized, one-year study. Psychopathology. 2016;49(5):345-55.
32. Khonsari NM, Badrfam R, Mohammdi MR, Rastad H, Etemadi F, Vafaei Z, Zandifar A. Effect of Aerobic Exercise as Adjunct Therapy on the Improvement of Negative Symptoms and Cognitive Impairment in Patients With Schizophrenia: A Randomized, Case-Control Clinical Trial. Journal of psychosocial nursing and mental health services. 2022 May 1;60(5):38-43.
33. Kilbourne AM, Barbaresso MM, Lai Z, Nord KM, Bramlet M, Goodrich DE, Post EP, Almirall D, Bauer MS. Improving physical health in patients with chronic mental disorders: 12-month results from a randomized controlled collaborative care trial. The Journal of clinical psychiatry. 2017 Jan;78(1):129.
34. Kimhy D, Khan S, Ayanrouh L, Chang RW, Hansen MC, Lister A, Ballon JS, Vakhrusheva J, Armstrong HF, Bartels MN, Sloan RP. Use of active-play video games to enhance aerobic fitness in schizophrenia: feasibility, safety, and adherence. Psychiatric Services. 2016 Feb 1;67(2):240-3.
35. Kimhy D, Vakhrusheva J, Bartels MN, Armstrong HF, Ballon JS, Khan S, Chang RW, Hansen MC, Ayanruoh L, Lister A, Castrén E. The impact of aerobic exercise on brain-derived neurotrophic factor and neurocognition in individuals with schizophrenia: a single-blind, randomized clinical trial. Schizophrenia bulletin. 2015 Jul 1;41(4):859-68.
36. Leutwyler H, Hubbard EM, Jeste DV, Miller B, Vinogradov S. Associations of schizophrenia symptoms and neurocognition with physical activity in older adults with schizophrenia. Biological research for nursing. 2014 Jan;16(1):23-30.
37. Lin J, Chan SK, Lee EH, Chang WC, Tse M, Su WW, Sham P, Hui CL, Joe G, Chan CL, Khong PL. Aerobic exercise and yoga improve neurocognitive function in women with early psychosis. NPJ schizophrenia. 2015 Dec 2;1(1):1-7.
38. Lin J, Geng X, Lee EH, Chan SK, Chang WC, Hui CL, Tse M, Chan CL, Khong PL, Honer WG, Chen EY. Yoga reduces the brain's amplitude of low-frequency fluctuations in patients with early psychosis results of a randomized controlled trial. Schizophrenia research. 2017 Jun;184:141-2.
39. Looijmans A, Jörg F, Bruggeman R, Schoevers R, Corpeleijn E. Design of the Lifestyle Interventions for severe mentally ill Outpatients in the Netherlands (LION) trial; a cluster randomised controlled study of a multidimensional web tool intervention to improve cardiometabolic health in patients with severe mental illness. BMC psychiatry. 2017 Dec 1;17(1):107.
40. Looijmans A, Jörg F, Schoevers RA, Bruggeman R, Stolk RP, Corpeleijn E. Changing the obesogenic environment of severe mentally ill residential patients: ELIPS, a cluster randomised study design. BMC psychiatry. 2014 Dec 1;14(1):293.
41. Lovell K, Wearden A, Bradshaw T, Tomenson B, Pedley R, Davies LM, Husain N, Woodham A, Escott D, Swarbrick CM. An exploratory randomized controlled study of a healthy living intervention in early intervention services for psychosis: the INTERvention to encourage ACTivity, improve diet, and reduce weight gain (INTERACT) study. The Journal of clinical psychiatry. 2014 May 15;75(5):498-505.
42. Marzolini S, Jensen B, Melville P. Feasibility and effects of a group-based resistance and aerobic exercise program for individuals with severe schizophrenia: a multidisciplinary approach. Mental Health and Physical Activity. 2009 Jun 1;2(1):29-36.
43. Mazyarkin Z, Peleg T, Golani I, Sharony L, Kremer I, Shamir A. Health benefits of a physical exercise program for inpatients with mental health; a pilot study. Journal of psychiatric research. 2019 Jun 1;113:10-6.
44. Ng F, Dodd S, Berk M. The effects of physical activity in the acute treatment of bipolar disorder: a pilot study. Journal of affective disorders. 2007 Aug 1;101(1-3):259-62.
45. Paikkatt B, Singh AR, Singh PK, Jahan M, Ranjan JK. Efficacy of Yoga therapy for the management of psychopathology of patients having chronic schizophrenia. Indian journal of psychiatry. 2015 Oct;57(4):355.
46. Paikkatt B, Singh AR, Singh PK, Jahan M. Efficacy of yoga therapy on subjective well-being and basic living skills of patients having chronic schizophrenia. Industrial psychiatry journal. 2012 Jul;21(2):109.
47. Priebe S, Savill M, Wykes T, Bentall RP, Reininghaus U, Lauber C, Bremner S, Eldridge S, Röhricht F. Effectiveness of group body psychotherapy for negative symptoms of schizophrenia: multicentre randomised controlled trial. The British Journal of Psychiatry. 2016 Jul;209(1):54-61.
48. Romain AJ, Fankam C, Karelis AD, Letendre E, Mikolajczak G, Stip E, Abdel-Baki A. Effects of high intensity interval training among overweight individuals with psychotic disorders: A randomized controlled trial. Schizophrenia research. 2019 Aug 1;210:278-86.
49. Ryu J, Jung JH, Kim J, Kim CH, Lee HB, Kim DH, Lee SK, Shin JH, Roh D. Outdoor cycling improves clinical symptoms, cognition and objectively measured physical activity in patients with schizophrenia: A randomized controlled trial. Journal of psychiatric research. 2020 Jan 1;120:144-53.
50. Scheewe TW, van Haren NE, Sarkisyan G, Schnack HG, Brouwer RM, de Glint M, Pol HE, Backx FJ, Kahn RS, Cahn W. Exercise therapy, cardiorespiratory fitness and their effect on brain volumes: a randomised controlled trial in patients with schizophrenia and healthy controls. European Neuropsychopharmacology. 2013 Jul 1;23(7):675-85.
51. Shimada T, Ito S, Makabe A, Yamanushi A, Takenaka A, Kobayashi M. Aerobic exercise and cognitive functioning in schizophrenia: A pilot randomized controlled trial. Psychiatry Research. 2019 Dec 1;282:112638.
52. Skrinar GS, Huxley NA, Hutchinson DS, Menninger E, Glew P. The role of a fitness intervention on people with serious psychiatric disabilities. Psychiatric rehabilitation journal. 2005;29(2):122.
53. Stiekema AP, Looijmans A, van der Meer L, Bruggeman R, Schoevers RA, Corpeleijn E, Jörg F. Effects of a lifestyle intervention on psychosocial well-being of severe mentally ill residential patients: ELIPS, a cluster randomized controlled pragmatic trial. Schizophrenia research. 2018 Sep 1;199:407-13.
54. Van Citters AD, Pratt SI, Jue K, Williams G, Miller PT, Xie H, Bartels SJ. A pilot evaluation of the In SHAPE individualized health promotion intervention for adults with mental illness. Community mental health journal. 2010 Dec 1;46(6):540-52.
55. Vancampfort D, De Hert M, Knapen J, Wampers M, Demunter H, Deckx S, Maurissen K, Probst M. State anxiety, psychological stress and positive well-being responses to yoga and aerobic exercise in people with schizophrenia: a pilot study. Disability and rehabilitation. 2011 Jan 1;33(8):684-9.
56. Visceglia E, Lewis S. Yoga therapy as an adjunctive treatment for schizophrenia: a randomized, controlled pilot study. The Journal of Alternative and Complementary Medicine. 2011 Jul 1;17(7):601-7.
57. Weinstock LM, Broughton MK, Tezanos KM, Tremont G, Gillette T, Uebelacker LA. Adjunctive yoga versus bibliotherapy for bipolar depression: a pilot randomized controlled trial. Mental Health and Physical Activity. 2016 Oct 1;11:67-73.
58. Williams J, Stubbs B, Richardson S, Flower C, Barr-Hamilton L, Grey B, Hubbard K, Spaducci G, Gaughran F, Craig T. ‘Walk this way’: results from a pilot randomised controlled trial of a health coaching intervention to reduce sedentary behaviour and increase physical activity in people with serious mental illness. BMC psychiatry. 2019 Dec 1;19(1):287.
59. Wolff E, Gaudlitz K, von Lindenberger BL, Plag J, Heinz A, Ströhle A. Exercise and physical activity in mental disorders. European archives of psychiatry and clinical neuroscience. 2011 Nov 1;261(2):186.
60. Woodward ML, Gicas KM, Warburton DE, White RF, Rauscher A, Leonova O, Su W, Smith GN, Thornton AE, Vertinsky AT, Phillips AA. Hippocampal volume and vasculature before and after exercise in treatment-resistant schizophrenia. Schizophrenia Research. 2018 Dec 1;202:158-65.
61. Woodward ML, Lin J, Gicas KM, Su W, Hui CL, Honer WG, Chen EY, Lang DJ. Medial temporal lobe cortical changes in response to exercise interventions in people with early psychosis: A randomized controlled trial. Schizophrenia Research. 2020 May 30.
62. Wu MK, Wang CK, Bai YM, Huang CY, Lee SD. Outcomes of obese, clozapine-treated inpatients with schizophrenia placed on a six-month diet and physical activity program. Psychiatric Services. 2007 Apr;58(4):544-50.
63. Yarborough BJ, Leo MC, Stumbo S, Perrin NA, Green CA. STRIDE: a randomized trial of a lifestyle intervention to promote weight loss among individuals taking antipsychotic medications. BMC psychiatry. 2013 Dec 1;13(1):238.

# II. Supplementary Tables

## Supplementary Table 1. Scopus search strategy

| Query string |
| --- |
| ( TITLE-ABS-KEY ( schizophrenia  OR  bipolar  OR  manic  AND depressive  OR  psychosis ) )  AND  ( TITLE-ABS-KEY ( exercise  OR  exercise  AND therapy  OR  physical  AND activity ) )  AND  ( TITLE-ABS-KEY ( randomized  AND controlled  OR  randomized  AND controlled  AND trial  OR  rct  OR  randomized  AND control  AND trial ) ) |

## Supplementary Table 2. Database findings

| **Online Database** | **Findings** |
| --- | --- |
| Medline (via EBSCO) | 436 |
| Scopus | 25 |
| PsychInfo (Ovid) | 97 |
| Total | 558 |

## Supplementary Table 3. Summary of review's authors judgments on risk of bias

**Attux et al 2013**

| Bias | Criteria for judging risk of bias in the Risk of Bias assessment tool | Authors’ judgement | Support for judgement |
| --- | --- | --- | --- |
|  |  |  |  |
| Random Sequence Generation  (Selection Bias) | Criteria for a judgement of ‘Low risk’ of bias  The investigators describe a random component in the sequence generation process such as:   - Referring to a random number table; - Using a computer random number generator; - Coin tossing; - Shuffling cards or envelopes; - Throwing dice; - Drawing of lots; - Minimization.   Criteria for the judgement of ‘High risk’ of bias  The investigators describe a non-random component in the sequence generation process. Usually, the description would involve some systematic, non-random approach, for example:   - Sequence generated by odd or even date of birth; - Sequence generated by some rule based on date (or day) of admission; - Sequence generated by some rule based on hospital or clinic record number.   Other non-random approaches happen much less frequently than the systematic approaches mentioned above and tend to be obvious.  They usually involve judgement or some method of non-random categorization of participants, for example:   - Allocation by judgement of the clinician; - Allocation by preference of the participant; - Allocation based on the results of a laboratory test or a series of tests; - Allocation by availability of the intervention   Criteria for the judgement of ‘Unclear risk’ of bias  Insufficient information about the sequence generation process to permit judgement of ‘Low risk’ or ‘High risk’. | Low risk | Quote 1: "Participants who agreed to take part in the study signed written informed consent and were randomly assigned to the intervention group or a standard care group using a randomization table available on the web site [www.randomization.com](http://www.randomization.com)” |
| Allocation Concealment (Selection Bias) | Criteria for a judgement of ‘Low risk’ of bias  Participants and investigators enrolling participants could not foresee assignment because one of the following, or an equivalent method, was used to conceal allocation:   - Central allocation (including telephone, web-based and pharmacy-controlled randomization); - Sequentially numbered drug containers of identical appearance; - Sequentially numbered, opaque, sealed envelopes.   Criteria for the judgement of ‘High risk’ of bias  Participants or investigators enrolling participants could possibly foresee assignments and thus introduce selection bias, such as allocation based on:   - Using an open random allocation schedule (e.g. a list of random numbers); - Assignment envelopes were used without appropriate safeguards (e.g. if envelopes were unsealed or non­opaque or not sequentially numbered); - Alternation or rotation; - Date of birth; - Case record number; - Any other explicitly unconcealed procedure.   Criteria for the judgement of ‘Unclear risk’ of bias  Insufficient information to permit judgement of ‘Low risk’ or ‘High risk’. | Unclear risk | Comment: Not described within the study. |
| Blinding of Participants And Personnel  (Performance Bias)  All Outcomes | Criteria for a judgement of ‘Low risk’ of bias   - No blinding or incomplete blinding, but the review authors judge that the outcome is not likely to be influenced by lack of blinding; - Blinding of participants and key study personnel ensured, and unlikely that the blinding could have been broken.   Criteria for the judgement of ‘High risk’ of bias   - No blinding or incomplete blinding, and the outcome is likely to be influenced by lack of blinding; - Blinding of key study participants and personnel attempted, but likely that the blinding could have been broken, and the outcome is likely to be influenced by lack of blinding.   Criteria for the judgement of ‘Unclear risk’ of bias   - Insufficient information to permit judgement of ‘Low risk’ or ‘High risk’; - The study did not address this outcome | Unclear risk | Comment: Not described within the study. |
| Blinding of Outcome Assessment  (Detection Bias)  All Outcomes | Criteria for a judgement of ‘Low risk’ of bias   - No blinding of outcome assessment, but the review authors judge that the outcome measurement is not likely to be influenced by lack of blinding; - Blinding of outcome assessment ensured, and unlikely that the blinding could have been broken.   Criteria for the judgement of ‘High risk’ of bias   - No blinding of outcome assessment, and the outcome measurement is likely to be influenced by lack of blinding; - Blinding of outcome assessment, but likely that the blinding could have been broken, and the outcome measurement is likely to be influenced by lack of blinding.   Criteria for the judgement of ‘Unclear risk’ of bias   - Insufficient information to permit judgement of ‘Low risk’ or ‘High risk’; - The study did not address this outcome | Low risk | Quote: "Blind investigators applied the following instruments to participants of the trial at baseline and three month follow up…” |
| Incomplete Outcome Data  (Attrition Bias)  All Outcomes | Criteria for a judgement of ‘Low risk’ of bias   - No missing outcome data; - Reasons for missing outcome data unlikely to be related to true outcome (for survival data, censoring unlikely to be introducing bias); - Missing outcome data balanced in numbers across intervention groups, with similar reasons for missing data across groups; - For dichotomous outcome data, the proportion of missing outcomes compared with observed event risk not enough to have a clinically relevant impact on the intervention effect estimate; - For continuous outcome data, plausible effect size (difference in means or standardized difference in means) among missing outcomes not enough to have a clinically relevant impact on observed effect size; - Missing data have been imputed using appropriate methods.   Criteria for the judgement of ‘High risk’ of bias   - Reason for missing outcome data likely to be related to true outcome, with either imbalance in numbers or reasons for missing data across intervention groups; - For dichotomous outcome data, the proportion of missing outcomes compared with observed event risk enough to induce clinically relevant bias in intervention effect estimate; - For continuous outcome data, plausible effect size (difference in means or standardized difference in means) among missing outcomes enough to induce clinically relevant bias in observed effect size; - ‘As-treated’ analysis done with substantial departure of the intervention received from that assigned at randomization; - Potentially inappropriate application of simple imputation.   Criteria for the judgement of ‘Unclear risk’ of bias   - Insufficient information to permit judgement of ‘Low risk’ or ‘High risk’; - The study did not address this outcome | Low risk | Quote: “Analysis of the main outcomes were based on the intention-to-treat analysis with the Last Observation Carried Forward” |
| Selective Reporting (Reporting Bias) | Criteria for a judgement of ‘Low risk’ of bias   - The study protocol is available and all of the study’s pre-specified (primary and secondary) outcomes that are of interest in the review have been reported in the pre-specified way; - The study protocol is not available but it is clear that the published reports include all expected outcomes, including those that were pre-specified (convincing text of this nature may be uncommon).   Criteria for the judgement of ‘High risk’ of bias   - Not all of the study’s pre-specified primary outcomes have been reported; - One or more primary outcomes is reported using measurements, analysis methods or subsets of the data (e.g. subscales) that were not pre-specified; - One or more reported primary outcomes were not pre-specified (unless clear justification for their reporting is provided, such as an unexpected adverse effect); - One or more outcomes of interest in the review are reported incompletely so that they cannot be entered in a meta-analysis; - The study report fails to include results for a key outcome that would be expected to have been reported for such a study   Criteria for the judgement of ‘Unclear risk’ of bias  Insufficient information to permit judgement of ‘Low risk’ or ‘High risk’. It is likely that the majority of studies will fall into this category. | High risk | Comments: A study protocol is not available therefore, unable to determine if all of the study’s pre-specified primary outcomes were reported. |
| Other Bias | Criteria for a judgement of ‘Low risk’ of bias  Trials not published in the list of suspected predatory journals presented by Manca et al. 2017.  Criteria for the judgement of ‘High risk’ of bias  Trials published in the list of suspected predatory journals presented by Manca et al. 2017. | Low Risk | comments: The trial is not published in the list of suspected predatory journals presented by Manca et al. 2017. |

**Bang-Kittilsen et al 2020**

| Bias | Criteria for judging risk of bias in the Risk of Bias assessment tool | Authors’ judgement | Support for judgement |
| --- | --- | --- | --- |
| Random Sequence Generation  (Selection Bias) | Criteria for a judgement of ‘Low risk’ of bias  The investigators describe a random component in the sequence generation process such as:   - Referring to a random number table; - Using a computer random number generator; - Coin tossing; - Shuffling cards or envelopes; - Throwing dice; - Drawing of lots; - Minimization.   Criteria for the judgement of ‘High risk’ of bias  The investigators describe a non-random component in the sequence generation process. Usually, the description would involve some systematic, non-random approach, for example:   - Sequence generated by odd or even date of birth; - Sequence generated by some rule based on date (or day) of admission; - Sequence generated by some rule based on hospital or clinic record number.   Other non-random approaches happen much less frequently than the systematic approaches mentioned above and tend to be obvious.  They usually involve judgement or some method of non-random categorization of participants, for example:   - Allocation by judgement of the clinician; - Allocation by preference of the participant; - Allocation based on the results of a laboratory test or a series of tests; - Allocation by availability of the intervention   Criteria for the judgement of ‘Unclear risk’ of bias  Insufficient information about the sequence generation process to permit judgement of ‘Low risk’ or ‘High risk’. | Low risk | Quote: "Eighty-two participants were included and randomly allocated to HITT (n=43 or AVG (n=39) by a study coordinator using concealed envelopes” |
| Allocation Concealment (Selection Bias) | Criteria for a judgement of ‘Low risk’ of bias  Participants and investigators enrolling participants could not foresee assignment because one of the following, or an equivalent method, was used to conceal allocation:   - Central allocation (including telephone, web-based and pharmacy-controlled randomization); - Sequentially numbered drug containers of identical appearance; - Sequentially numbered, opaque, sealed envelopes.   Criteria for the judgement of ‘High risk’ of bias  Participants or investigators enrolling participants could possibly foresee assignments and thus introduce selection bias, such as allocation based on:   - Using an open random allocation schedule (e.g. a list of random numbers); - Assignment envelopes were used without appropriate safeguards (e.g. if envelopes were unsealed or non­opaque or not sequentially numbered); - Alternation or rotation; - Date of birth; - Case record number; - Any other explicitly unconcealed procedure.   Criteria for the judgement of ‘Unclear risk’ of bias  Insufficient information to permit judgement of ‘Low risk’ or ‘High risk’. | Low risk | Quote: "The allocation sequence, generated by a computerized random number generator, kept treatment assignment unpredictable by varying the size of the stratification blocks. Allocation was performed blinded for baseline assessment results, but equal distribution of baseline CRF in the two groups was obtained by stratification on the expected median peak VO2 based on the results from the feasibility study.” |
| Blinding of Participants And Personnel  (Performance Bias)  All Outcomes | Criteria for a judgement of ‘Low risk’ of bias   - No blinding or incomplete blinding, but the review authors judge that the outcome is not likely to be influenced by lack of blinding; - Blinding of participants and key study personnel ensured, and unlikely that the blinding could have been broken.   Criteria for the judgement of ‘High risk’ of bias   - No blinding or incomplete blinding, and the outcome is likely to be influenced by lack of blinding; - Blinding of key study participants and personnel attempted, but likely that the blinding could have been broken, and the outcome is likely to be influenced by lack of blinding.   Criteria for the judgement of ‘Unclear risk’ of bias   - Insufficient information to permit judgement of ‘Low risk’ or ‘High risk’; - The study did not address this outcome | High risk | Quote: "Allocation, interventions and assessments were conducted separately and participants from the two groups never met. Due to the study design, the participants could not be blinded. An intervention team (a physiotherapist, a nurse with a master’s degree in sport education and four mental health care workers) was trained to supervise both interventions and could not be blinded” |
| Blinding of Outcome Assessment  (Detection Bias)  All Outcomes | Criteria for a judgement of ‘Low risk’ of bias   - No blinding of outcome assessment, but the review authors judge that the outcome measurement is not likely to be influenced by lack of blinding; - Blinding of outcome assessment ensured, and unlikely that the blinding could have been broken.   Criteria for the judgement of ‘High risk’ of bias   - No blinding of outcome assessment, and the outcome measurement is likely to be influenced by lack of blinding; - Blinding of outcome assessment, but likely that the blinding could have been broken, and the outcome measurement is likely to be influenced by lack of blinding.   Criteria for the judgement of ‘Unclear risk’ of bias   - Insufficient information to permit judgement of ‘Low risk’ or ‘High risk’; - The study did not address this outcome | Unclear risk | Comment: Not described within the study. |
| Incomplete Outcome Data  (Attrition Bias)  All Outcomes | Criteria for a judgement of ‘Low risk’ of bias   - No missing outcome data; - Reasons for missing outcome data unlikely to be related to true outcome (for survival data, censoring unlikely to be introducing bias); - Missing outcome data balanced in numbers across intervention groups, with similar reasons for missing data across groups; - For dichotomous outcome data, the proportion of missing outcomes compared with observed event risk not enough to have a clinically relevant impact on the intervention effect estimate; - For continuous outcome data, plausible effect size (difference in means or standardized difference in means) among missing outcomes not enough to have a clinically relevant impact on observed effect size; - Missing data have been imputed using appropriate methods.   Criteria for the judgement of ‘High risk’ of bias   - Reason for missing outcome data likely to be related to true outcome, with either imbalance in numbers or reasons for missing data across intervention groups; - For dichotomous outcome data, the proportion of missing outcomes compared with observed event risk enough to induce clinically relevant bias in intervention effect estimate; - For continuous outcome data, plausible effect size (difference in means or standardized difference in means) among missing outcomes enough to induce clinically relevant bias in observed effect size; - ‘As-treated’ analysis done with substantial departure of the intervention received from that assigned at randomization; - Potentially inappropriate application of simple imputation.   Criteria for the judgement of ‘Unclear risk’ of bias   - Insufficient information to permit judgement of ‘Low risk’ or ‘High risk’; - The study did not address this outcome | Low risk | Quote: “The attrition rate was 13% at the post-intervention assessment. Including participants lost to follow-up the total attrition rate at the end of the study was 31%. Protocol violations were registered for 10 participants. They were allowed to stay in the study to reduce missing data and to enable both IIT analysis of all randomized participants.” |
| Selective Reporting (Reporting Bias) | Criteria for a judgement of ‘Low risk’ of bias   - The study protocol is available and all of the study’s pre-specified (primary and secondary) outcomes that are of interest in the review have been reported in the pre-specified way; - The study protocol is not available but it is clear that the published reports include all expected outcomes, including those that were pre-specified (convincing text of this nature may be uncommon).   Criteria for the judgement of ‘High risk’ of bias   - Not all of the study’s pre-specified primary outcomes have been reported; - One or more primary outcomes is reported using measurements, analysis methods or subsets of the data (e.g. subscales) that were not pre-specified; - One or more reported primary outcomes were not pre-specified (unless clear justification for their reporting is provided, such as an unexpected adverse effect); - One or more outcomes of interest in the review are reported incompletely so that they cannot be entered in a meta-analysis; - The study report fails to include results for a key outcome that would be expected to have been reported for such a study   Criteria for the judgement of ‘Unclear risk’ of bias  Insufficient information to permit judgement of ‘Low risk’ or ‘High risk’. It is likely that the majority of studies will fall into this category. | Low risk | Quote: "The study reports the primary outcome data from the Effects of Physical Activity in Psychosis Study (EPHAPS), pre-reported in ClinicalTrials.gov (NCT02205684, first posted 31.07.14)” |
| Other Bias | Criteria for a judgement of ‘Low risk’ of bias  Trials not published in the list of suspected predatory journals presented by Manca et al. 2017.  Criteria for the judgement of ‘High risk’ of bias  Trials published in the list of suspected predatory journals presented by Manca et al. 2017. | Low Risk | comments: The trial is not published in the list of suspected predatory journals presented by Manca et al. 2017. |

**Beebe et al 2009**

| Bias | Criteria for judging risk of bias in the Risk of Bias assessment tool | Authors’ judgement | Support for judgement |
| --- | --- | --- | --- |
|  |  |  |  |
| Random Sequence Generation  (Selection Bias) | Criteria for a judgement of ‘Low risk’ of bias  The investigators describe a random component in the sequence generation process such as:   - Referring to a random number table; - Using a computer random number generator; - Coin tossing; - Shuffling cards or envelopes; - Throwing dice; - Drawing of lots; - Minimization.   Criteria for the judgement of ‘High risk’ of bias  The investigators describe a non-random component in the sequence generation process. Usually, the description would involve some systematic, non-random approach, for example:   - Sequence generated by odd or even date of birth; - Sequence generated by some rule based on date (or day) of admission; - Sequence generated by some rule based on hospital or clinic record number.   Other non-random approaches happen much less frequently than the systematic approaches mentioned above and tend to be obvious.  They usually involve judgement or some method of non-random categorization of participants, for example:   - Allocation by judgement of the clinician; - Allocation by preference of the participant; - Allocation based on the results of a laboratory test or a series of tests; - Allocation by availability of the intervention   Criteria for the judgement of ‘Unclear risk’ of bias  Insufficient information about the sequence generation process to permit judgement of ‘Low risk’ or ‘High risk’. | Unclear risk | Quote: “The 12 persons agreeing to participate were randomly assigned to experimental (n=6) or control groups (n=6) using a randomization schedule designed by the statistician”  Unclear what randomization schedule means |
| Allocation Concealment (Selection Bias) | Criteria for a judgement of ‘Low risk’ of bias  Participants and investigators enrolling participants could not foresee assignment because one of the following, or an equivalent method, was used to conceal allocation:   - Central allocation (including telephone, web-based and pharmacy-controlled randomization); - Sequentially numbered drug containers of identical appearance; - Sequentially numbered, opaque, sealed envelopes.   Criteria for the judgement of ‘High risk’ of bias  Participants or investigators enrolling participants could possibly foresee assignments and thus introduce selection bias, such as allocation based on:   - Using an open random allocation schedule (e.g. a list of random numbers); - Assignment envelopes were used without appropriate safeguards (e.g. if envelopes were unsealed or non­opaque or not sequentially numbered); - Alternation or rotation; - Date of birth; - Case record number; - Any other explicitly unconcealed procedure.   Criteria for the judgement of ‘Unclear risk’ of bias  Insufficient information to permit judgement of ‘Low risk’ or ‘High risk’. | Unclear risk | Comment: Not described within the study. |
| Blinding of Participants And Personnel  (Performance Bias)  All Outcomes | Criteria for a judgement of ‘Low risk’ of bias   - No blinding or incomplete blinding, but the review authors judge that the outcome is not likely to be influenced by lack of blinding; - Blinding of participants and key study personnel ensured, and unlikely that the blinding could have been broken.   Criteria for the judgement of ‘High risk’ of bias   - No blinding or incomplete blinding, and the outcome is likely to be influenced by lack of blinding; - Blinding of key study participants and personnel attempted, but likely that the blinding could have been broken, and the outcome is likely to be influenced by lack of blinding.   Criteria for the judgement of ‘Unclear risk’ of bias   - Insufficient information to permit judgement of ‘Low risk’ or ‘High risk’; - The study did not address this outcome | Unclear risk | Comment: Not described within the study. |
| Blinding of Outcome Assessment  (Detection Bias)  All Outcomes | Criteria for a judgement of ‘Low risk’ of bias   - No blinding of outcome assessment, but the review authors judge that the outcome measurement is not likely to be influenced by lack of blinding; - Blinding of outcome assessment ensured, and unlikely that the blinding could have been broken.   Criteria for the judgement of ‘High risk’ of bias   - No blinding of outcome assessment, and the outcome measurement is likely to be influenced by lack of blinding; - Blinding of outcome assessment, but likely that the blinding could have been broken, and the outcome measurement is likely to be influenced by lack of blinding.   Criteria for the judgement of ‘Unclear risk’ of bias   - Insufficient information to permit judgement of ‘Low risk’ or ‘High risk’; - The study did not address this outcome | Unclear risk | Comment: Not described within the study. |
| Incomplete Outcome Data  (Attrition Bias)  All Outcomes | Criteria for a judgement of ‘Low risk’ of bias   - No missing outcome data; - Reasons for missing outcome data unlikely to be related to true outcome (for survival data, censoring unlikely to be introducing bias); - Missing outcome data balanced in numbers across intervention groups, with similar reasons for missing data across groups; - For dichotomous outcome data, the proportion of missing outcomes compared with observed event risk not enough to have a clinically relevant impact on the intervention effect estimate; - For continuous outcome data, plausible effect size (difference in means or standardized difference in means) among missing outcomes not enough to have a clinically relevant impact on observed effect size; - Missing data have been imputed using appropriate methods.   Criteria for the judgement of ‘High risk’ of bias   - Reason for missing outcome data likely to be related to true outcome, with either imbalance in numbers or reasons for missing data across intervention groups; - For dichotomous outcome data, the proportion of missing outcomes compared with observed event risk enough to induce clinically relevant bias in intervention effect estimate; - For continuous outcome data, plausible effect size (difference in means or standardized difference in means) among missing outcomes enough to induce clinically relevant bias in observed effect size; - ‘As-treated’ analysis done with substantial departure of the intervention received from that assigned at randomization; - Potentially inappropriate application of simple imputation.   Criteria for the judgement of ‘Unclear risk’ of bias   - Insufficient information to permit judgement of ‘Low risk’ or ‘High risk’; - The study did not address this outcome | High risk | Quote: “Attendance ranged from 43% to 91% of session attended out of the total sessions offered; 75% of participants attended more than half of the session and 50% attended 2/3 of the session. The most common reason reported for a missed exercise session as conflict with another appointment.”  Comment: nothing was stated about intention to treat analysis for any missing data |
| Selective Reporting (Reporting Bias) | Criteria for a judgement of ‘Low risk’ of bias   - The study protocol is available and all of the study’s pre-specified (primary and secondary) outcomes that are of interest in the review have been reported in the pre-specified way; - The study protocol is not available but it is clear that the published reports include all expected outcomes, including those that were pre-specified (convincing text of this nature may be uncommon).   Criteria for the judgement of ‘High risk’ of bias   - Not all of the study’s pre-specified primary outcomes have been reported; - One or more primary outcomes is reported using measurements, analysis methods or subsets of the data (e.g. subscales) that were not pre-specified; - One or more reported primary outcomes were not pre-specified (unless clear justification for their reporting is provided, such as an unexpected adverse effect); - One or more outcomes of interest in the review are reported incompletely so that they cannot be entered in a meta-analysis; - The study report fails to include results for a key outcome that would be expected to have been reported for such a study   Criteria for the judgement of ‘Unclear risk’ of bias  Insufficient information to permit judgement of ‘Low risk’ or ‘High risk’. It is likely that the majority of studies will fall into this category. | High risk | Comments: A study protocol is not available therefore, unable to determine if all of the study’s pre-specified primary outcomes were reported. |
| Other Bias | Criteria for a judgement of ‘Low risk’ of bias  Trials not published in the list of suspected predatory journals presented by Manca et al. 2017.  Criteria for the judgement of ‘High risk’ of bias  Trials published in the list of suspected predatory journals presented by Manca et al. 2017. | Low Risk | comments: The trial is not published in the list of suspected predatory journals presented by Manca et al. 2017. |

**Brobakken et al 2019**

| Bias | Criteria for judging risk of bias in the Risk of Bias assessment tool | Authors’ judgement | Support for judgement |
| --- | --- | --- | --- |
|  |  |  |  |
| Random Sequence Generation  (Selection Bias) | Criteria for a judgement of ‘Low risk’ of bias  The investigators describe a random component in the sequence generation process such as:   - Referring to a random number table; - Using a computer random number generator; - Coin tossing; - Shuffling cards or envelopes; - Throwing dice; - Drawing of lots; - Minimization.   Criteria for the judgement of ‘High risk’ of bias  The investigators describe a non-random component in the sequence generation process. Usually, the description would involve some systematic, non-random approach, for example:   - Sequence generated by odd or even date of birth; - Sequence generated by some rule based on date (or day) of admission; - Sequence generated by some rule based on hospital or clinic record number.   Other non-random approaches happen much less frequently than the systematic approaches mentioned above and tend to be obvious.  They usually involve judgement or some method of non-random categorization of participants, for example:   - Allocation by judgement of the clinician; - Allocation by preference of the participant; - Allocation based on the results of a laboratory test or a series of tests; - Allocation by availability of the intervention   Criteria for the judgement of ‘Unclear risk’ of bias  Insufficient information about the sequence generation process to permit judgement of ‘Low risk’ or ‘High risk’. | Low risk | Quote: “A computer based randomization method was used to randomly select patients to either the treatment group or the control group in a 1:1 ratio” |
| Allocation Concealment (Selection Bias) | Criteria for a judgement of ‘Low risk’ of bias  Participants and investigators enrolling participants could not foresee assignment because one of the following, or an equivalent method, was used to conceal allocation:   - Central allocation (including telephone, web-based and pharmacy-controlled randomization); - Sequentially numbered drug containers of identical appearance; - Sequentially numbered, opaque, sealed envelopes.   Criteria for the judgement of ‘High risk’ of bias  Participants or investigators enrolling participants could possibly foresee assignments and thus introduce selection bias, such as allocation based on:   - Using an open random allocation schedule (e.g. a list of random numbers); - Assignment envelopes were used without appropriate safeguards (e.g. if envelopes were unsealed or non­opaque or not sequentially numbered); - Alternation or rotation; - Date of birth; - Case record number; - Any other explicitly unconcealed procedure.   Criteria for the judgement of ‘Unclear risk’ of bias  Insufficient information to permit judgement of ‘Low risk’ or ‘High risk’. | Unclear risk | Quote: “The unit for Applied Clinical Research at the university carried out the randomization procedures to ensure blinded allocation”  Comment: There is not adequate information on allocation concealment. |
| Blinding of Participants And Personnel  (Performance Bias)  All Outcomes | Criteria for a judgement of ‘Low risk’ of bias   - No blinding or incomplete blinding, but the review authors judge that the outcome is not likely to be influenced by lack of blinding; - Blinding of participants and key study personnel ensured, and unlikely that the blinding could have been broken.   Criteria for the judgement of ‘High risk’ of bias   - No blinding or incomplete blinding, and the outcome is likely to be influenced by lack of blinding; - Blinding of key study participants and personnel attempted, but likely that the blinding could have been broken, and the outcome is likely to be influenced by lack of blinding.   Criteria for the judgement of ‘Unclear risk’ of bias   - Insufficient information to permit judgement of ‘Low risk’ or ‘High risk’; - The study did not address this outcome | High risk | Quote: "All treadmill tests were conducted by the same non-blinded exercise physiologist” |
| Blinding of Outcome Assessment  (Detection Bias)  All Outcomes | Criteria for a judgement of ‘Low risk’ of bias   - No blinding of outcome assessment, but the review authors judge that the outcome measurement is not likely to be influenced by lack of blinding; - Blinding of outcome assessment ensured, and unlikely that the blinding could have been broken.   Criteria for the judgement of ‘High risk’ of bias   - No blinding of outcome assessment, and the outcome measurement is likely to be influenced by lack of blinding; - Blinding of outcome assessment, but likely that the blinding could have been broken, and the outcome measurement is likely to be influenced by lack of blinding.   Criteria for the judgement of ‘Unclear risk’ of bias   - Insufficient information to permit judgement of ‘Low risk’ or ‘High risk’; - The study did not address this outcome | High risk | Quote: "All treadmill tests were conducted by the same non-blinded exercise physiologist” |
| Incomplete Outcome Data  (Attrition Bias)  All Outcomes | Criteria for a judgement of ‘Low risk’ of bias   - No missing outcome data; - Reasons for missing outcome data unlikely to be related to true outcome (for survival data, censoring unlikely to be introducing bias); - Missing outcome data balanced in numbers across intervention groups, with similar reasons for missing data across groups; - For dichotomous outcome data, the proportion of missing outcomes compared with observed event risk not enough to have a clinically relevant impact on the intervention effect estimate; - For continuous outcome data, plausible effect size (difference in means or standardized difference in means) among missing outcomes not enough to have a clinically relevant impact on observed effect size; - Missing data have been imputed using appropriate methods.   Criteria for the judgement of ‘High risk’ of bias   - Reason for missing outcome data likely to be related to true outcome, with either imbalance in numbers or reasons for missing data across intervention groups; - For dichotomous outcome data, the proportion of missing outcomes compared with observed event risk enough to induce clinically relevant bias in intervention effect estimate; - For continuous outcome data, plausible effect size (difference in means or standardized difference in means) among missing outcomes enough to induce clinically relevant bias in observed effect size; - ‘As-treated’ analysis done with substantial departure of the intervention received from that assigned at randomization; - Potentially inappropriate application of simple imputation.   Criteria for the judgement of ‘Unclear risk’ of bias   - Insufficient information to permit judgement of ‘Low risk’ or ‘High risk’; - The study did not address this outcome | Low risk | Comment: The dropout-rates were low, ITT analysis was carried out |
| Selective Reporting (Reporting Bias) | Criteria for a judgement of ‘Low risk’ of bias   - The study protocol is available and all of the study’s pre-specified (primary and secondary) outcomes that are of interest in the review have been reported in the pre-specified way; - The study protocol is not available but it is clear that the published reports include all expected outcomes, including those that were pre-specified (convincing text of this nature may be uncommon).   Criteria for the judgement of ‘High risk’ of bias   - Not all of the study’s pre-specified primary outcomes have been reported; - One or more primary outcomes is reported using measurements, analysis methods or subsets of the data (e.g. subscales) that were not pre-specified; - One or more reported primary outcomes were not pre-specified (unless clear justification for their reporting is provided, such as an unexpected adverse effect); - One or more outcomes of interest in the review are reported incompletely so that they cannot be entered in a meta-analysis; - The study report fails to include results for a key outcome that would be expected to have been reported for such a study   Criteria for the judgement of ‘Unclear risk’ of bias  Insufficient information to permit judgement of ‘Low risk’ or ‘High risk’. It is likely that the majority of studies will fall into this category. | Low risk | Comment: Protocol is published at ClinicalTrials.gov. Identifier: NCT02743143 |
| Other Bias | Criteria for a judgement of ‘Low risk’ of bias  Trials not published in the list of suspected predatory journals presented by Manca et al. 2017.  Criteria for the judgement of ‘High risk’ of bias  Trials published in the list of suspected predatory journals presented by Manca et al. 2017. | Low Risk | comments: The trial is not published in the list of suspected predatory journals presented by Manca et al. 2017. |

**Curcic et al 2017**

| Bias | Criteria for judging risk of bias in the Risk of Bias assessment tool | Authors’ judgement | Support for judgement |
| --- | --- | --- | --- |
|  |  |  |  |
| Random Sequence Generation  (Selection Bias) | Criteria for a judgement of ‘Low risk’ of bias  The investigators describe a random component in the sequence generation process such as:   - Referring to a random number table; - Using a computer random number generator; - Coin tossing; - Shuffling cards or envelopes; - Throwing dice; - Drawing of lots; - Minimization.   Criteria for the judgement of ‘High risk’ of bias  The investigators describe a non-random component in the sequence generation process. Usually, the description would involve some systematic, non-random approach, for example:   - Sequence generated by odd or even date of birth; - Sequence generated by some rule based on date (or day) of admission; - Sequence generated by some rule based on hospital or clinic record number.   Other non-random approaches happen much less frequently than the systematic approaches mentioned above and tend to be obvious.  They usually involve judgement or some method of non-random categorization of participants, for example:   - Allocation by judgement of the clinician; - Allocation by preference of the participant; - Allocation based on the results of a laboratory test or a series of tests; - Allocation by availability of the intervention   Criteria for the judgement of ‘Unclear risk’ of bias  Insufficient information about the sequence generation process to permit judgement of ‘Low risk’ or ‘High risk’. | Unclear risk | Quote: "The 80 participants agreeing to participate were randomly assigned to experimental (40 patients) or control (40 patients) by using simple randomization method”  Not clear which randomization method |
| Allocation Concealment (Selection Bias) | Criteria for a judgement of ‘Low risk’ of bias  Participants and investigators enrolling participants could not foresee assignment because one of the following, or an equivalent method, was used to conceal allocation:   - Central allocation (including telephone, web-based and pharmacy-controlled randomization); - Sequentially numbered drug containers of identical appearance; - Sequentially numbered, opaque, sealed envelopes.   Criteria for the judgement of ‘High risk’ of bias  Participants or investigators enrolling participants could possibly foresee assignments and thus introduce selection bias, such as allocation based on:   - Using an open random allocation schedule (e.g. a list of random numbers); - Assignment envelopes were used without appropriate safeguards (e.g. if envelopes were unsealed or non­opaque or not sequentially numbered); - Alternation or rotation; - Date of birth; - Case record number; - Any other explicitly unconcealed procedure.   Criteria for the judgement of ‘Unclear risk’ of bias  Insufficient information to permit judgement of ‘Low risk’ or ‘High risk’. | Unclear risk | Comment: There is not adequate information on allocation concealment. |
| Blinding of Participants And Personnel  (Performance Bias)  All Outcomes | Criteria for a judgement of ‘Low risk’ of bias   - No blinding or incomplete blinding, but the review authors judge that the outcome is not likely to be influenced by lack of blinding; - Blinding of participants and key study personnel ensured, and unlikely that the blinding could have been broken.   Criteria for the judgement of ‘High risk’ of bias   - No blinding or incomplete blinding, and the outcome is likely to be influenced by lack of blinding; - Blinding of key study participants and personnel attempted, but likely that the blinding could have been broken, and the outcome is likely to be influenced by lack of blinding.   Criteria for the judgement of ‘Unclear risk’ of bias   - Insufficient information to permit judgement of ‘Low risk’ or ‘High risk’; - The study did not address this outcome | Unclear risk | Comment: Blinding of participants and personnel not possible. However, no description of the blinding strategies were described |
| Blinding of Outcome Assessment  (Detection Bias)  All Outcomes | Criteria for a judgement of ‘Low risk’ of bias   - No blinding of outcome assessment, but the review authors judge that the outcome measurement is not likely to be influenced by lack of blinding; - Blinding of outcome assessment ensured, and unlikely that the blinding could have been broken.   Criteria for the judgement of ‘High risk’ of bias   - No blinding of outcome assessment, and the outcome measurement is likely to be influenced by lack of blinding; - Blinding of outcome assessment, but likely that the blinding could have been broken, and the outcome measurement is likely to be influenced by lack of blinding.   Criteria for the judgement of ‘Unclear risk’ of bias   - Insufficient information to permit judgement of ‘Low risk’ or ‘High risk’; - The study did not address this outcome | Unclear risk | Comment: No description of whether blinded personnel performed the outcome assessments |
| Incomplete Outcome Data  (Attrition Bias)  All Outcomes | Criteria for a judgement of ‘Low risk’ of bias   - No missing outcome data; - Reasons for missing outcome data unlikely to be related to true outcome (for survival data, censoring unlikely to be introducing bias); - Missing outcome data balanced in numbers across intervention groups, with similar reasons for missing data across groups; - For dichotomous outcome data, the proportion of missing outcomes compared with observed event risk not enough to have a clinically relevant impact on the intervention effect estimate; - For continuous outcome data, plausible effect size (difference in means or standardized difference in means) among missing outcomes not enough to have a clinically relevant impact on observed effect size; - Missing data have been imputed using appropriate methods.   Criteria for the judgement of ‘High risk’ of bias   - Reason for missing outcome data likely to be related to true outcome, with either imbalance in numbers or reasons for missing data across intervention groups; - For dichotomous outcome data, the proportion of missing outcomes compared with observed event risk enough to induce clinically relevant bias in intervention effect estimate; - For continuous outcome data, plausible effect size (difference in means or standardized difference in means) among missing outcomes enough to induce clinically relevant bias in observed effect size; - ‘As-treated’ analysis done with substantial departure of the intervention received from that assigned at randomization; - Potentially inappropriate application of simple imputation.   Criteria for the judgement of ‘Unclear risk’ of bias   - Insufficient information to permit judgement of ‘Low risk’ or ‘High risk’; - The study did not address this outcome | Unclear risk | Comment: No description of the number of participants dropping out, or adherence. No comment on intention-to-treat analyses |
| Selective Reporting (Reporting Bias) | Criteria for a judgement of ‘Low risk’ of bias   - The study protocol is available and all of the study’s pre-specified (primary and secondary) outcomes that are of interest in the review have been reported in the pre-specified way; - The study protocol is not available but it is clear that the published reports include all expected outcomes, including those that were pre-specified (convincing text of this nature may be uncommon).   Criteria for the judgement of ‘High risk’ of bias   - Not all of the study’s pre-specified primary outcomes have been reported; - One or more primary outcomes is reported using measurements, analysis methods or subsets of the data (e.g. subscales) that were not pre-specified; - One or more reported primary outcomes were not pre-specified (unless clear justification for their reporting is provided, such as an unexpected adverse effect); - One or more outcomes of interest in the review are reported incompletely so that they cannot be entered in a meta-analysis; - The study report fails to include results for a key outcome that would be expected to have been reported for such a study   Criteria for the judgement of ‘Unclear risk’ of bias  Insufficient information to permit judgement of ‘Low risk’ or ‘High risk’. It is likely that the majority of studies will fall into this category. | High risk | Comments: A study protocol is not available therefore, unable to determine if all of the study’s pre-specified primary outcomes were reported. |
| Other Bias | Criteria for a judgement of ‘Low risk’ of bias  Trials not published in the list of suspected predatory journals presented by Manca et al. 2017.  Criteria for the judgement of ‘High risk’ of bias  Trials published in the list of suspected predatory journals presented by Manca et al. 2017. | Low Risk | comments: The trial is not published in the list of suspected predatory journals presented by Manca et al. 2017. |

**Duraiswamy et al 2007**

| Bias | Criteria for judging risk of bias in the Risk of Bias assessment tool | Authors’ judgement | Support for judgement |
| --- | --- | --- | --- |
|  |  |  |  |
| Random Sequence Generation  (Selection Bias) | Criteria for a judgement of ‘Low risk’ of bias  The investigators describe a random component in the sequence generation process such as:   - Referring to a random number table; - Using a computer random number generator; - Coin tossing; - Shuffling cards or envelopes; - Throwing dice; - Drawing of lots; - Minimization.   Criteria for the judgement of ‘High risk’ of bias  The investigators describe a non-random component in the sequence generation process. Usually, the description would involve some systematic, non-random approach, for example:   - Sequence generated by odd or even date of birth; - Sequence generated by some rule based on date (or day) of admission; - Sequence generated by some rule based on hospital or clinic record number.   Other non-random approaches happen much less frequently than the systematic approaches mentioned above and tend to be obvious.  They usually involve judgement or some method of non-random categorization of participants, for example:   - Allocation by judgement of the clinician; - Allocation by preference of the participant; - Allocation based on the results of a laboratory test or a series of tests; - Allocation by availability of the intervention   Criteria for the judgement of ‘Unclear risk’ of bias  Insufficient information about the sequence generation process to permit judgement of ‘Low risk’ or ‘High risk’. | Low risk | Quote: "After the baseline assessments were done, subjects were randomly assigned using a computer-generated random number table to receive either YT (n=31) or PT (n=30)” |
| Allocation Concealment (Selection Bias) | Criteria for a judgement of ‘Low risk’ of bias  Participants and investigators enrolling participants could not foresee assignment because one of the following, or an equivalent method, was used to conceal allocation:   - Central allocation (including telephone, web-based and pharmacy-controlled randomization); - Sequentially numbered drug containers of identical appearance; - Sequentially numbered, opaque, sealed envelopes.   Criteria for the judgement of ‘High risk’ of bias  Participants or investigators enrolling participants could possibly foresee assignments and thus introduce selection bias, such as allocation based on:   - Using an open random allocation schedule (e.g. a list of random numbers); - Assignment envelopes were used without appropriate safeguards (e.g. if envelopes were unsealed or non­opaque or not sequentially numbered); - Alternation or rotation; - Date of birth; - Case record number; - Any other explicitly unconcealed procedure.   Criteria for the judgement of ‘Unclear risk’ of bias  Insufficient information to permit judgement of ‘Low risk’ or ‘High risk’. | Unclear risk | Comment: There is not adequate information on allocation concealment. |
| Blinding of Participants And Personnel  (Performance Bias)  All Outcomes | Criteria for a judgement of ‘Low risk’ of bias   - No blinding or incomplete blinding, but the review authors judge that the outcome is not likely to be influenced by lack of blinding; - Blinding of participants and key study personnel ensured, and unlikely that the blinding could have been broken.   Criteria for the judgement of ‘High risk’ of bias   - No blinding or incomplete blinding, and the outcome is likely to be influenced by lack of blinding; - Blinding of key study participants and personnel attempted, but likely that the blinding could have been broken, and the outcome is likely to be influenced by lack of blinding.   Criteria for the judgement of ‘Unclear risk’ of bias   - Insufficient information to permit judgement of ‘Low risk’ or ‘High risk’; - The study did not address this outcome | Unclear risk | Comment: Blinding of participants and personnel not possible. However, no description of the blinding strategies were described |
| Blinding of Outcome Assessment  (Detection Bias)  All Outcomes | Criteria for a judgement of ‘Low risk’ of bias   - No blinding of outcome assessment, but the review authors judge that the outcome measurement is not likely to be influenced by lack of blinding; - Blinding of outcome assessment ensured, and unlikely that the blinding could have been broken.   Criteria for the judgement of ‘High risk’ of bias   - No blinding of outcome assessment, and the outcome measurement is likely to be influenced by lack of blinding; - Blinding of outcome assessment, but likely that the blinding could have been broken, and the outcome measurement is likely to be influenced by lack of blinding.   Criteria for the judgement of ‘Unclear risk’ of bias   - Insufficient information to permit judgement of ‘Low risk’ or ‘High risk’; - The study did not address this outcome | Unclear risk | Comment: No description of whether blinded personnel performed the outcome assessments |
| Incomplete Outcome Data  (Attrition Bias)  All Outcomes | Criteria for a judgement of ‘Low risk’ of bias   - No missing outcome data; - Reasons for missing outcome data unlikely to be related to true outcome (for survival data, censoring unlikely to be introducing bias); - Missing outcome data balanced in numbers across intervention groups, with similar reasons for missing data across groups; - For dichotomous outcome data, the proportion of missing outcomes compared with observed event risk not enough to have a clinically relevant impact on the intervention effect estimate; - For continuous outcome data, plausible effect size (difference in means or standardized difference in means) among missing outcomes not enough to have a clinically relevant impact on observed effect size; - Missing data have been imputed using appropriate methods.   Criteria for the judgement of ‘High risk’ of bias   - Reason for missing outcome data likely to be related to true outcome, with either imbalance in numbers or reasons for missing data across intervention groups; - For dichotomous outcome data, the proportion of missing outcomes compared with observed event risk enough to induce clinically relevant bias in intervention effect estimate; - For continuous outcome data, plausible effect size (difference in means or standardized difference in means) among missing outcomes enough to induce clinically relevant bias in observed effect size; - ‘As-treated’ analysis done with substantial departure of the intervention received from that assigned at randomization; - Potentially inappropriate application of simple imputation.   Criteria for the judgement of ‘Unclear risk’ of bias   - Insufficient information to permit judgement of ‘Low risk’ or ‘High risk’; - The study did not address this outcome | Low risk | Quote: “All but four subjects who completed 3 weeks of training were available for follow-up assessment after 4 months”  Comment: There was a low attrition rate and the groups did not differ statistically |
| Selective Reporting (Reporting Bias) | Criteria for a judgement of ‘Low risk’ of bias   - The study protocol is available and all of the study’s pre-specified (primary and secondary) outcomes that are of interest in the review have been reported in the pre-specified way; - The study protocol is not available but it is clear that the published reports include all expected outcomes, including those that were pre-specified (convincing text of this nature may be uncommon).   Criteria for the judgement of ‘High risk’ of bias   - Not all of the study’s pre-specified primary outcomes have been reported; - One or more primary outcomes is reported using measurements, analysis methods or subsets of the data (e.g. subscales) that were not pre-specified; - One or more reported primary outcomes were not pre-specified (unless clear justification for their reporting is provided, such as an unexpected adverse effect); - One or more outcomes of interest in the review are reported incompletely so that they cannot be entered in a meta-analysis; - The study report fails to include results for a key outcome that would be expected to have been reported for such a study   Criteria for the judgement of ‘Unclear risk’ of bias  Insufficient information to permit judgement of ‘Low risk’ or ‘High risk’. It is likely that the majority of studies will fall into this category. | High risk | Comments: A study protocol is not available therefore, unable to determine if all of the study’s pre-specified primary outcomes were reported. |
| Other Bias | Criteria for a judgement of ‘Low risk’ of bias  Trials not published in the list of suspected predatory journals presented by Manca et al. 2017.  Criteria for the judgement of ‘High risk’ of bias  Trials published in the list of suspected predatory journals presented by Manca et al. 2017. | Low Risk | comments: The trial is not published in the list of suspected predatory journals presented by Manca et al. 2017. |

**Heggelung et al 2011**

| Bias | Criteria for judging risk of bias in the Risk of Bias assessment tool | Authors’ judgement | Support for judgement |
| --- | --- | --- | --- |
|  |  |  |  |
| Random Sequence Generation  (Selection Bias) | Criteria for a judgement of ‘Low risk’ of bias  The investigators describe a random component in the sequence generation process such as:   - Referring to a random number table; - Using a computer random number generator; - Coin tossing; - Shuffling cards or envelopes; - Throwing dice; - Drawing of lots; - Minimization.   Criteria for the judgement of ‘High risk’ of bias  The investigators describe a non-random component in the sequence generation process. Usually, the description would involve some systematic, non-random approach, for example:   - Sequence generated by odd or even date of birth; - Sequence generated by some rule based on date (or day) of admission; - Sequence generated by some rule based on hospital or clinic record number.   Other non-random approaches happen much less frequently than the systematic approaches mentioned above and tend to be obvious.  They usually involve judgement or some method of non-random categorization of participants, for example:   - Allocation by judgement of the clinician; - Allocation by preference of the participant; - Allocation based on the results of a laboratory test or a series of tests; - Allocation by availability of the intervention   Criteria for the judgement of ‘Unclear risk’ of bias  Insufficient information about the sequence generation process to permit judgement of ‘Low risk’ or ‘High risk’. | High risk | Quote: "A limitation of the study was that we did not conduct a random allocation of subjects to the two groups, but included consecutive patients first to the HIT group then to the CG group. Some patients with schizophrenia have distrust in randomization, and randomized controlled trials might exclude patients with ah high level of symptoms from participating” |
| Allocation Concealment (Selection Bias) | Criteria for a judgement of ‘Low risk’ of bias  Participants and investigators enrolling participants could not foresee assignment because one of the following, or an equivalent method, was used to conceal allocation:   - Central allocation (including telephone, web-based and pharmacy-controlled randomization); - Sequentially numbered drug containers of identical appearance; - Sequentially numbered, opaque, sealed envelopes.   Criteria for the judgement of ‘High risk’ of bias  Participants or investigators enrolling participants could possibly foresee assignments and thus introduce selection bias, such as allocation based on:   - Using an open random allocation schedule (e.g. a list of random numbers); - Assignment envelopes were used without appropriate safeguards (e.g. if envelopes were unsealed or non­opaque or not sequentially numbered); - Alternation or rotation; - Date of birth; - Case record number; - Any other explicitly unconcealed procedure.   Criteria for the judgement of ‘Unclear risk’ of bias  Insufficient information to permit judgement of ‘Low risk’ or ‘High risk’. | High risk | Quote: "A limitation of the study was that we did not conduct a random allocation of subjects to the two groups, but included consecutive patients first to the HIT group then to the CG group. Some patients with schizophrenia have distrust in randomization, and randomized controlled trials might exclude patients with ah high level of symptoms from participating” |
| Blinding of Participants And Personnel  (Performance Bias)  All Outcomes | Criteria for a judgement of ‘Low risk’ of bias   - No blinding or incomplete blinding, but the review authors judge that the outcome is not likely to be influenced by lack of blinding; - Blinding of participants and key study personnel ensured, and unlikely that the blinding could have been broken.   Criteria for the judgement of ‘High risk’ of bias   - No blinding or incomplete blinding, and the outcome is likely to be influenced by lack of blinding; - Blinding of key study participants and personnel attempted, but likely that the blinding could have been broken, and the outcome is likely to be influenced by lack of blinding.   Criteria for the judgement of ‘Unclear risk’ of bias   - Insufficient information to permit judgement of ‘Low risk’ or ‘High risk’; - The study did not address this outcome | High risk | Quote: “Testing was not done blinded to allocation.” |
| Blinding of Outcome Assessment  (Detection Bias)  All Outcomes | Criteria for a judgement of ‘Low risk’ of bias   - No blinding of outcome assessment, but the review authors judge that the outcome measurement is not likely to be influenced by lack of blinding; - Blinding of outcome assessment ensured, and unlikely that the blinding could have been broken.   Criteria for the judgement of ‘High risk’ of bias   - No blinding of outcome assessment, and the outcome measurement is likely to be influenced by lack of blinding; - Blinding of outcome assessment, but likely that the blinding could have been broken, and the outcome measurement is likely to be influenced by lack of blinding.   Criteria for the judgement of ‘Unclear risk’ of bias   - Insufficient information to permit judgement of ‘Low risk’ or ‘High risk’; - The study did not address this outcome | High risk | Quote: “Testing was not done blinded to allocation.” |
| Incomplete Outcome Data  (Attrition Bias)  All Outcomes | Criteria for a judgement of ‘Low risk’ of bias   - No missing outcome data; - Reasons for missing outcome data unlikely to be related to true outcome (for survival data, censoring unlikely to be introducing bias); - Missing outcome data balanced in numbers across intervention groups, with similar reasons for missing data across groups; - For dichotomous outcome data, the proportion of missing outcomes compared with observed event risk not enough to have a clinically relevant impact on the intervention effect estimate; - For continuous outcome data, plausible effect size (difference in means or standardized difference in means) among missing outcomes not enough to have a clinically relevant impact on observed effect size; - Missing data have been imputed using appropriate methods.   Criteria for the judgement of ‘High risk’ of bias   - Reason for missing outcome data likely to be related to true outcome, with either imbalance in numbers or reasons for missing data across intervention groups; - For dichotomous outcome data, the proportion of missing outcomes compared with observed event risk enough to induce clinically relevant bias in intervention effect estimate; - For continuous outcome data, plausible effect size (difference in means or standardized difference in means) among missing outcomes enough to induce clinically relevant bias in observed effect size; - ‘As-treated’ analysis done with substantial departure of the intervention received from that assigned at randomization; - Potentially inappropriate application of simple imputation.   Criteria for the judgement of ‘Unclear risk’ of bias   - Insufficient information to permit judgement of ‘Low risk’ or ‘High risk’; - The study did not address this outcome | High risk | Quote: “During the training period, six of the 25 included patients did not complete the study and are not included in the results” |
| Selective Reporting (Reporting Bias) | Criteria for a judgement of ‘Low risk’ of bias   - The study protocol is available and all of the study’s pre-specified (primary and secondary) outcomes that are of interest in the review have been reported in the pre-specified way; - The study protocol is not available but it is clear that the published reports include all expected outcomes, including those that were pre-specified (convincing text of this nature may be uncommon).   Criteria for the judgement of ‘High risk’ of bias   - Not all of the study’s pre-specified primary outcomes have been reported; - One or more primary outcomes is reported using measurements, analysis methods or subsets of the data (e.g. subscales) that were not pre-specified; - One or more reported primary outcomes were not pre-specified (unless clear justification for their reporting is provided, such as an unexpected adverse effect); - One or more outcomes of interest in the review are reported incompletely so that they cannot be entered in a meta-analysis; - The study report fails to include results for a key outcome that would be expected to have been reported for such a study   Criteria for the judgement of ‘Unclear risk’ of bias  Insufficient information to permit judgement of ‘Low risk’ or ‘High risk’. It is likely that the majority of studies will fall into this category. | Low risk | Comment: The study was registered at ClinicalTrials.gov. Identifier: NCT00286299 |
| Other Bias | Criteria for a judgement of ‘Low risk’ of bias  Trials not published in the list of suspected predatory journals presented by Manca et al. 2017.  Criteria for the judgement of ‘High risk’ of bias  Trials published in the list of suspected predatory journals presented by Manca et al. 2017. | Low Risk | Comments: The trial is not published in the list of suspected predatory journals presented by Manca et al. 2017. |

**Ikai et al 2013**

| Bias | Criteria for judging risk of bias in the Risk of Bias assessment tool | Authors’ judgement | Support for judgement |
| --- | --- | --- | --- |
|  |  |  |  |
| Random Sequence Generation  (Selection Bias) | Criteria for a judgement of ‘Low risk’ of bias  The investigators describe a random component in the sequence generation process such as:   - Referring to a random number table; - Using a computer random number generator; - Coin tossing; - Shuffling cards or envelopes; - Throwing dice; - Drawing of lots; - Minimization.   Criteria for the judgement of ‘High risk’ of bias  The investigators describe a non-random component in the sequence generation process. Usually, the description would involve some systematic, non-random approach, for example:   - Sequence generated by odd or even date of birth; - Sequence generated by some rule based on date (or day) of admission; - Sequence generated by some rule based on hospital or clinic record number.   Other non-random approaches happen much less frequently than the systematic approaches mentioned above and tend to be obvious.  They usually involve judgement or some method of non-random categorization of participants, for example:   - Allocation by judgement of the clinician; - Allocation by preference of the participant; - Allocation based on the results of a laboratory test or a series of tests; - Allocation by availability of the intervention   Criteria for the judgement of ‘Unclear risk’ of bias  Insufficient information about the sequence generation process to permit judgement of ‘Low risk’ or ‘High risk’. | Low risk | Quote: "Subjects were randomly assigned to either of the following two groups: a yoga therapy group and a regular day-care group. Randomization with a usage of sealed opaque envelopes performed by *Doctors* *in the hospital* who were not involved in the study.” |
| Allocation Concealment (Selection Bias) | Criteria for a judgement of ‘Low risk’ of bias  Participants and investigators enrolling participants could not foresee assignment because one of the following, or an equivalent method, was used to conceal allocation:   - Central allocation (including telephone, web-based and pharmacy-controlled randomization); - Sequentially numbered drug containers of identical appearance; - Sequentially numbered, opaque, sealed envelopes.   Criteria for the judgement of ‘High risk’ of bias  Participants or investigators enrolling participants could possibly foresee assignments and thus introduce selection bias, such as allocation based on:   - Using an open random allocation schedule (e.g. a list of random numbers); - Assignment envelopes were used without appropriate safeguards (e.g. if envelopes were unsealed or non­opaque or not sequentially numbered); - Alternation or rotation; - Date of birth; - Case record number; - Any other explicitly unconcealed procedure.   Criteria for the judgement of ‘Unclear risk’ of bias  Insufficient information to permit judgement of ‘Low risk’ or ‘High risk’. | Low risk | Quote: "The randomization list without any stratification or blocks was made with a use of computer program, and opaque envelopes were opened after the baseline assessment” |
| Blinding of Participants And Personnel  (Performance Bias)  All Outcomes | Criteria for a judgement of ‘Low risk’ of bias   - No blinding or incomplete blinding, but the review authors judge that the outcome is not likely to be influenced by lack of blinding; - Blinding of participants and key study personnel ensured, and unlikely that the blinding could have been broken.   Criteria for the judgement of ‘High risk’ of bias   - No blinding or incomplete blinding, and the outcome is likely to be influenced by lack of blinding; - Blinding of key study participants and personnel attempted, but likely that the blinding could have been broken, and the outcome is likely to be influenced by lack of blinding.   Criteria for the judgement of ‘Unclear risk’ of bias   - Insufficient information to permit judgement of ‘Low risk’ or ‘High risk’; - The study did not address this outcome | High risk | Quote: “due to the single-blind study design, the participants in the yoga group might have been expecting favourable effects of the intervention while the bodily stability, in contrast to some psychological measures appears to be less affect psychologically. It would be unrealistic to conduct a double-blind RCT” |
| Blinding of Outcome Assessment  (Detection Bias)  All Outcomes | Criteria for a judgement of ‘Low risk’ of bias   - No blinding of outcome assessment, but the review authors judge that the outcome measurement is not likely to be influenced by lack of blinding; - Blinding of outcome assessment ensured, and unlikely that the blinding could have been broken.   Criteria for the judgement of ‘High risk’ of bias   - No blinding of outcome assessment, and the outcome measurement is likely to be influenced by lack of blinding; - Blinding of outcome assessment, but likely that the blinding could have been broken, and the outcome measurement is likely to be influenced by lack of blinding.   Criteria for the judgement of ‘Unclear risk’ of bias   - Insufficient information to permit judgement of ‘Low risk’ or ‘High risk’; - The study did not address this outcome | Low risk | Quote: “due to the single-blind study design, the participants in the yoga group might have been expecting favourable effects of the intervention while the bodily stability, in contrast to some psychological measures appears to be less affect psychologically. It would be unrealistic to conduct a double-blind RCT”  Comment: The assessors were blinded to subject allocation. |
| Incomplete Outcome Data  (Attrition Bias)  All Outcomes | Criteria for a judgement of ‘Low risk’ of bias   - No missing outcome data; - Reasons for missing outcome data unlikely to be related to true outcome (for survival data, censoring unlikely to be introducing bias); - Missing outcome data balanced in numbers across intervention groups, with similar reasons for missing data across groups; - For dichotomous outcome data, the proportion of missing outcomes compared with observed event risk not enough to have a clinically relevant impact on the intervention effect estimate; - For continuous outcome data, plausible effect size (difference in means or standardized difference in means) among missing outcomes not enough to have a clinically relevant impact on observed effect size; - Missing data have been imputed using appropriate methods.   Criteria for the judgement of ‘High risk’ of bias   - Reason for missing outcome data likely to be related to true outcome, with either imbalance in numbers or reasons for missing data across intervention groups; - For dichotomous outcome data, the proportion of missing outcomes compared with observed event risk enough to induce clinically relevant bias in intervention effect estimate; - For continuous outcome data, plausible effect size (difference in means or standardized difference in means) among missing outcomes enough to induce clinically relevant bias in observed effect size; - ‘As-treated’ analysis done with substantial departure of the intervention received from that assigned at randomization; - Potentially inappropriate application of simple imputation.   Criteria for the judgement of ‘Unclear risk’ of bias   - Insufficient information to permit judgement of ‘Low risk’ or ‘High risk’; - The study did not address this outcome | Low risk | Quote: “Analyses were performed on an intent-to-treat basis, and data were reported using a last-observation carried forward method.” |
| Selective Reporting (Reporting Bias) | Criteria for a judgement of ‘Low risk’ of bias   - The study protocol is available and all of the study’s pre-specified (primary and secondary) outcomes that are of interest in the review have been reported in the pre-specified way; - The study protocol is not available but it is clear that the published reports include all expected outcomes, including those that were pre-specified (convincing text of this nature may be uncommon).   Criteria for the judgement of ‘High risk’ of bias   - Not all of the study’s pre-specified primary outcomes have been reported; - One or more primary outcomes is reported using measurements, analysis methods or subsets of the data (e.g. subscales) that were not pre-specified; - One or more reported primary outcomes were not pre-specified (unless clear justification for their reporting is provided, such as an unexpected adverse effect); - One or more outcomes of interest in the review are reported incompletely so that they cannot be entered in a meta-analysis; - The study report fails to include results for a key outcome that would be expected to have been reported for such a study   Criteria for the judgement of ‘Unclear risk’ of bias  Insufficient information to permit judgement of ‘Low risk’ or ‘High risk’. It is likely that the majority of studies will fall into this category. | Low risk | Quote: “The study was registered at the University Medical Information Network Clinical Trial Registery (Identifier: UMIN 000008201)” |
| Other Bias | Criteria for a judgement of ‘Low risk’ of bias  Trials not published in the list of suspected predatory journals presented by Manca et al. 2017.  Criteria for the judgement of ‘High risk’ of bias  Trials published in the list of suspected predatory journals presented by Manca et al. 2017. | Low Risk | comments: The trial is not published in the list of suspected predatory journals presented by Manca et al. 2017. |

**Ikai et al 2014**

| Bias | Criteria for judging risk of bias in the Risk of Bias assessment tool | Authors’ judgement | Support for judgement |
| --- | --- | --- | --- |
|  |  |  |  |
| Random Sequence Generation  (Selection Bias) | Criteria for a judgement of ‘Low risk’ of bias  The investigators describe a random component in the sequence generation process such as:   - Referring to a random number table; - Using a computer random number generator; - Coin tossing; - Shuffling cards or envelopes; - Throwing dice; - Drawing of lots; - Minimization.   Criteria for the judgement of ‘High risk’ of bias  The investigators describe a non-random component in the sequence generation process. Usually, the description would involve some systematic, non-random approach, for example:   - Sequence generated by odd or even date of birth; - Sequence generated by some rule based on date (or day) of admission; - Sequence generated by some rule based on hospital or clinic record number.   Other non-random approaches happen much less frequently than the systematic approaches mentioned above and tend to be obvious.  They usually involve judgement or some method of non-random categorization of participants, for example:   - Allocation by judgement of the clinician; - Allocation by preference of the participant; - Allocation based on the results of a laboratory test or a series of tests; - Allocation by availability of the intervention   Criteria for the judgement of ‘Unclear risk’ of bias  Insufficient information about the sequence generation process to permit judgement of ‘Low risk’ or ‘High risk’. | Low risk | Quote: " The randomization was performed  by using sealed envelopes prepared by physicians at Yamanashi Prefectural Kita Hospital who were not involved in this study” |
| Allocation Concealment (Selection Bias) | Criteria for a judgement of ‘Low risk’ of bias  Participants and investigators enrolling participants could not foresee assignment because one of the following, or an equivalent method, was used to conceal allocation:   - Central allocation (including telephone, web-based and pharmacy-controlled randomization); - Sequentially numbered drug containers of identical appearance; - Sequentially numbered, opaque, sealed envelopes.   Criteria for the judgement of ‘High risk’ of bias  Participants or investigators enrolling participants could possibly foresee assignments and thus introduce selection bias, such as allocation based on:   - Using an open random allocation schedule (e.g. a list of random numbers); - Assignment envelopes were used without appropriate safeguards (e.g. if envelopes were unsealed or non­opaque or not sequentially numbered); - Alternation or rotation; - Date of birth; - Case record number; - Any other explicitly unconcealed procedure.   Criteria for the judgement of ‘Unclear risk’ of bias  Insufficient information to permit judgement of ‘Low risk’ or ‘High risk’. | Low risk | Quote: " A simple randomization list with  no special stratification or blocks was made by using a computer program. One of the four physicians opened the envelopes after the baseline assessment” |
| Blinding of Participants And Personnel  (Performance Bias)  All Outcomes | Criteria for a judgement of ‘Low risk’ of bias   - No blinding or incomplete blinding, but the review authors judge that the outcome is not likely to be influenced by lack of blinding; - Blinding of participants and key study personnel ensured, and unlikely that the blinding could have been broken.   Criteria for the judgement of ‘High risk’ of bias   - No blinding or incomplete blinding, and the outcome is likely to be influenced by lack of blinding; - Blinding of key study participants and personnel attempted, but likely that the blinding could have been broken, and the outcome is likely to be influenced by lack of blinding.   Criteria for the judgement of ‘Unclear risk’ of bias   - Insufficient information to permit judgement of ‘Low risk’ or ‘High risk’; - The study did not address this outcome | High risk | Quote: “it is impossible to conduct double-blind studies with yoga” |
| Blinding of Outcome Assessment  (Detection Bias)  All Outcomes | Criteria for a judgement of ‘Low risk’ of bias   - No blinding of outcome assessment, but the review authors judge that the outcome measurement is not likely to be influenced by lack of blinding; - Blinding of outcome assessment ensured, and unlikely that the blinding could have been broken.   Criteria for the judgement of ‘High risk’ of bias   - No blinding of outcome assessment, and the outcome measurement is likely to be influenced by lack of blinding; - Blinding of outcome assessment, but likely that the blinding could have been broken, and the outcome measurement is likely to be influenced by lack of blinding.   Criteria for the judgement of ‘Unclear risk’ of bias   - Insufficient information to permit judgement of ‘Low risk’ or ‘High risk’; - The study did not address this outcome | Low risk | Quote: " All assessments were performed by trained psychiatrists who were blind to the patients’ allocations and were not involved in the yoga therapy”  Comment: The assessor was blinded. |
| Incomplete Outcome Data  (Attrition Bias)  All Outcomes | Criteria for a judgement of ‘Low risk’ of bias   - No missing outcome data; - Reasons for missing outcome data unlikely to be related to true outcome (for survival data, censoring unlikely to be introducing bias); - Missing outcome data balanced in numbers across intervention groups, with similar reasons for missing data across groups; - For dichotomous outcome data, the proportion of missing outcomes compared with observed event risk not enough to have a clinically relevant impact on the intervention effect estimate; - For continuous outcome data, plausible effect size (difference in means or standardized difference in means) among missing outcomes not enough to have a clinically relevant impact on observed effect size; - Missing data have been imputed using appropriate methods.   Criteria for the judgement of ‘High risk’ of bias   - Reason for missing outcome data likely to be related to true outcome, with either imbalance in numbers or reasons for missing data across intervention groups; - For dichotomous outcome data, the proportion of missing outcomes compared with observed event risk enough to induce clinically relevant bias in intervention effect estimate; - For continuous outcome data, plausible effect size (difference in means or standardized difference in means) among missing outcomes enough to induce clinically relevant bias in observed effect size; - ‘As-treated’ analysis done with substantial departure of the intervention received from that assigned at randomization; - Potentially inappropriate application of simple imputation.   Criteria for the judgement of ‘Unclear risk’ of bias   - Insufficient information to permit judgement of ‘Low risk’ or ‘High risk’; - The study did not address this outcome | Low risk | Quote: “Main analyses were performed by using a last-observation-carried-forward method” |
| Selective Reporting (Reporting Bias) | Criteria for a judgement of ‘Low risk’ of bias   - The study protocol is available and all of the study’s pre-specified (primary and secondary) outcomes that are of interest in the review have been reported in the pre-specified way; - The study protocol is not available but it is clear that the published reports include all expected outcomes, including those that were pre-specified (convincing text of this nature may be uncommon).   Criteria for the judgement of ‘High risk’ of bias   - Not all of the study’s pre-specified primary outcomes have been reported; - One or more primary outcomes is reported using measurements, analysis methods or subsets of the data (e.g. subscales) that were not pre-specified; - One or more reported primary outcomes were not pre-specified (unless clear justification for their reporting is provided, such as an unexpected adverse effect); - One or more outcomes of interest in the review are reported incompletely so that they cannot be entered in a meta-analysis; - The study report fails to include results for a key outcome that would be expected to have been reported for such a study   Criteria for the judgement of ‘Unclear risk’ of bias  Insufficient information to permit judgement of ‘Low risk’ or ‘High risk’. It is likely that the majority of studies will fall into this category. | Low risk | Quote: “The study was registered at  the University Medical Information Network Clinical Trial Registry (Identifier: UMIN000009305).” |
| Other Bias | Criteria for a judgement of ‘Low risk’ of bias  Trials not published in the list of suspected predatory journals presented by Manca et al. 2017.  Criteria for the judgement of ‘High risk’ of bias  Trials published in the list of suspected predatory journals presented by Manca et al. 2017. | Low Risk | comments: The trial is not published in the list of suspected predatory journals presented by Manca et al. 2017. |

**Ikai et al 2017**

| Bias | Criteria for judging risk of bias in the Risk of Bias assessment tool | Authors’ judgement | Support for judgement |
| --- | --- | --- | --- |
|  |  |  |  |
| Random Sequence Generation  (Selection Bias) | Criteria for a judgement of ‘Low risk’ of bias  The investigators describe a random component in the sequence generation process such as:   - Referring to a random number table; - Using a computer random number generator; - Coin tossing; - Shuffling cards or envelopes; - Throwing dice; - Drawing of lots; - Minimization.   Criteria for the judgement of ‘High risk’ of bias  The investigators describe a non-random component in the sequence generation process. Usually, the description would involve some systematic, non-random approach, for example:   - Sequence generated by odd or even date of birth; - Sequence generated by some rule based on date (or day) of admission; - Sequence generated by some rule based on hospital or clinic record number.   Other non-random approaches happen much less frequently than the systematic approaches mentioned above and tend to be obvious.  They usually involve judgement or some method of non-random categorization of participants, for example:   - Allocation by judgement of the clinician; - Allocation by preference of the participant; - Allocation based on the results of a laboratory test or a series of tests; - Allocation by availability of the intervention   Criteria for the judgement of ‘Unclear risk’ of bias  Insufficient information about the sequence generation process to permit judgement of ‘Low risk’ or ‘High risk’. | Low risk | Quote:" Randomization was stratified for sex and age, and was performed using computers by a research assistant at Keio University who was otherwise not involved in this study.” |
| Allocation Concealment (Selection Bias) | Criteria for a judgement of ‘Low risk’ of bias  Participants and investigators enrolling participants could not foresee assignment because one of the following, or an equivalent method, was used to conceal allocation:   - Central allocation (including telephone, web-based and pharmacy-controlled randomization); - Sequentially numbered drug containers of identical appearance; - Sequentially numbered, opaque, sealed envelopes.   Criteria for the judgement of ‘High risk’ of bias  Participants or investigators enrolling participants could possibly foresee assignments and thus introduce selection bias, such as allocation based on:   - Using an open random allocation schedule (e.g. a list of random numbers); - Assignment envelopes were used without appropriate safeguards (e.g. if envelopes were unsealed or non­opaque or not sequentially numbered); - Alternation or rotation; - Date of birth; - Case record number; - Any other explicitly unconcealed procedure.   Criteria for the judgement of ‘Unclear risk’ of bias  Insufficient information to permit judgement of ‘Low risk’ or ‘High risk’. | Low risk | Quote:" Randomization was stratified for sex and age, and was performed using computers by a research assistant at Keio University who was otherwise not involved in this study.” |
| Blinding of Participants And Personnel  (Performance Bias)  All Outcomes | Criteria for a judgement of ‘Low risk’ of bias   - No blinding or incomplete blinding, but the review authors judge that the outcome is not likely to be influenced by lack of blinding; - Blinding of participants and key study personnel ensured, and unlikely that the blinding could have been broken.   Criteria for the judgement of ‘High risk’ of bias   - No blinding or incomplete blinding, and the outcome is likely to be influenced by lack of blinding; - Blinding of key study participants and personnel attempted, but likely that the blinding could have been broken, and the outcome is likely to be influenced by lack of blinding.   Criteria for the judgement of ‘Unclear risk’ of bias   - Insufficient information to permit judgement of ‘Low risk’ or ‘High risk’; - The study did not address this outcome | Low risk | Quote: “due to the single-blind study design, expectation bias among the participants in the yoga group remains a possibility although a number of objective measures were adopted, and it would have been technically challenging to conduct a double-blind RCT of chair yoga therapy” |
| Blinding of Outcome Assessment  (Detection Bias)  All Outcomes | Criteria for a judgement of ‘Low risk’ of bias   - No blinding of outcome assessment, but the review authors judge that the outcome measurement is not likely to be influenced by lack of blinding; - Blinding of outcome assessment ensured, and unlikely that the blinding could have been broken.   Criteria for the judgement of ‘High risk’ of bias   - No blinding of outcome assessment, and the outcome measurement is likely to be influenced by lack of blinding; - Blinding of outcome assessment, but likely that the blinding could have been broken, and the outcome measurement is likely to be influenced by lack of blinding.   Criteria for the judgement of ‘Unclear risk’ of bias   - Insufficient information to permit judgement of ‘Low risk’ or ‘High risk’; - The study did not address this outcome | Low risk | Comment: this was a single-blind study with the outcome assessor blinded |
| Incomplete Outcome Data  (Attrition Bias)  All Outcomes | Criteria for a judgement of ‘Low risk’ of bias   - No missing outcome data; - Reasons for missing outcome data unlikely to be related to true outcome (for survival data, censoring unlikely to be introducing bias); - Missing outcome data balanced in numbers across intervention groups, with similar reasons for missing data across groups; - For dichotomous outcome data, the proportion of missing outcomes compared with observed event risk not enough to have a clinically relevant impact on the intervention effect estimate; - For continuous outcome data, plausible effect size (difference in means or standardized difference in means) among missing outcomes not enough to have a clinically relevant impact on observed effect size; - Missing data have been imputed using appropriate methods.   Criteria for the judgement of ‘High risk’ of bias   - Reason for missing outcome data likely to be related to true outcome, with either imbalance in numbers or reasons for missing data across intervention groups; - For dichotomous outcome data, the proportion of missing outcomes compared with observed event risk enough to induce clinically relevant bias in intervention effect estimate; - For continuous outcome data, plausible effect size (difference in means or standardized difference in means) among missing outcomes enough to induce clinically relevant bias in observed effect size; - ‘As-treated’ analysis done with substantial departure of the intervention received from that assigned at randomization; - Potentially inappropriate application of simple imputation.   Criteria for the judgement of ‘Unclear risk’ of bias   - Insufficient information to permit judgement of ‘Low risk’ or ‘High risk’; - The study did not address this outcome | Low risk | Quote: “Analyses were performed on an intent-to-treat basis” |
| Selective Reporting (Reporting Bias) | Criteria for a judgement of ‘Low risk’ of bias   - The study protocol is available and all of the study’s pre-specified (primary and secondary) outcomes that are of interest in the review have been reported in the pre-specified way; - The study protocol is not available but it is clear that the published reports include all expected outcomes, including those that were pre-specified (convincing text of this nature may be uncommon).   Criteria for the judgement of ‘High risk’ of bias   - Not all of the study’s pre-specified primary outcomes have been reported; - One or more primary outcomes is reported using measurements, analysis methods or subsets of the data (e.g. subscales) that were not pre-specified; - One or more reported primary outcomes were not pre-specified (unless clear justification for their reporting is provided, such as an unexpected adverse effect); - One or more outcomes of interest in the review are reported incompletely so that they cannot be entered in a meta-analysis; - The study report fails to include results for a key outcome that would be expected to have been reported for such a study   Criteria for the judgement of ‘Unclear risk’ of bias  Insufficient information to permit judgement of ‘Low risk’ or ‘High risk’. It is likely that the majority of studies will fall into this category. | Low risk | Quote: “The study was registered at the University Medical Information Network Clinical Trial Registry (Identifier: UMIN000015711).” |
| Other Bias | Criteria for a judgement of ‘Low risk’ of bias  Trials not published in the list of suspected predatory journals presented by Manca et al. 2017.  Criteria for the judgement of ‘High risk’ of bias  Trials published in the list of suspected predatory journals presented by Manca et al. 2017. | Low Risk | comments: The trial is not published in the list of suspected predatory journals presented by Manca et al. 2017. |

**Kaltsatou et al 2015**

| Bias | Criteria for judging risk of bias in the Risk of Bias assessment tool | Authors’ judgement | Support for judgement |
| --- | --- | --- | --- |
|  |  |  |  |
| Random Sequence Generation  (Selection Bias) | Criteria for a judgement of ‘Low risk’ of bias  The investigators describe a random component in the sequence generation process such as:   - Referring to a random number table; - Using a computer random number generator; - Coin tossing; - Shuffling cards or envelopes; - Throwing dice; - Drawing of lots; - Minimization.   Criteria for the judgement of ‘High risk’ of bias  The investigators describe a non-random component in the sequence generation process. Usually, the description would involve some systematic, non-random approach, for example:   - Sequence generated by odd or even date of birth; - Sequence generated by some rule based on date (or day) of admission; - Sequence generated by some rule based on hospital or clinic record number.   Other non-random approaches happen much less frequently than the systematic approaches mentioned above and tend to be obvious.  They usually involve judgement or some method of non-random categorization of participants, for example:   - Allocation by judgement of the clinician; - Allocation by preference of the participant; - Allocation based on the results of a laboratory test or a series of tests; - Allocation by availability of the intervention   Criteria for the judgement of ‘Unclear risk’ of bias  Insufficient information about the sequence generation process to permit judgement of ‘Low risk’ or ‘High risk’. | Low risk | Quote: " Sedentary patients with schizophrenia were recruited from a psychiatric outpatient department  of American Hellenic Educational Progressive Association Hospital, in where they were inpatients, and were randomly assigned by simple random allocation (drawing lots) to a dance group (Group A) or to a control group (Group B).” |
| Allocation Concealment (Selection Bias) | Criteria for a judgement of ‘Low risk’ of bias  Participants and investigators enrolling participants could not foresee assignment because one of the following, or an equivalent method, was used to conceal allocation:   - Central allocation (including telephone, web-based and pharmacy-controlled randomization); - Sequentially numbered drug containers of identical appearance; - Sequentially numbered, opaque, sealed envelopes.   Criteria for the judgement of ‘High risk’ of bias  Participants or investigators enrolling participants could possibly foresee assignments and thus introduce selection bias, such as allocation based on:   - Using an open random allocation schedule (e.g. a list of random numbers); - Assignment envelopes were used without appropriate safeguards (e.g. if envelopes were unsealed or non­opaque or not sequentially numbered); - Alternation or rotation; - Date of birth; - Case record number; - Any other explicitly unconcealed procedure.   Criteria for the judgement of ‘Unclear risk’ of bias  Insufficient information to permit judgement of ‘Low risk’ or ‘High risk’. | Unclear risk | Quote: “Allocation sequence was concealed until intervention was assigned”  Not clear how it was concealed |
| Blinding of Participants And Personnel  (Performance Bias)  All Outcomes | Criteria for a judgement of ‘Low risk’ of bias   - No blinding or incomplete blinding, but the review authors judge that the outcome is not likely to be influenced by lack of blinding; - Blinding of participants and key study personnel ensured, and unlikely that the blinding could have been broken.   Criteria for the judgement of ‘High risk’ of bias   - No blinding or incomplete blinding, and the outcome is likely to be influenced by lack of blinding; - Blinding of key study participants and personnel attempted, but likely that the blinding could have been broken, and the outcome is likely to be influenced by lack of blinding.   Criteria for the judgement of ‘Unclear risk’ of bias   - Insufficient information to permit judgement of ‘Low risk’ or ‘High risk’; - The study did not address this outcome | High risk | Quote: “the participants were blind to pretest and posttest results.”  Comment: Blinding of participants and personnel was not possible due to nature of intervention. |
| Blinding of Outcome Assessment  (Detection Bias)  All Outcomes | Criteria for a judgement of ‘Low risk’ of bias   - No blinding of outcome assessment, but the review authors judge that the outcome measurement is not likely to be influenced by lack of blinding; - Blinding of outcome assessment ensured, and unlikely that the blinding could have been broken.   Criteria for the judgement of ‘High risk’ of bias   - No blinding of outcome assessment, and the outcome measurement is likely to be influenced by lack of blinding; - Blinding of outcome assessment, but likely that the blinding could have been broken, and the outcome measurement is likely to be influenced by lack of blinding.   Criteria for the judgement of ‘Unclear risk’ of bias   - Insufficient information to permit judgement of ‘Low risk’ or ‘High risk’; - The study did not address this outcome | Low risk | Quote: " Raters were blinded to the group”  Comment: The assessors were blinded. |
| Incomplete Outcome Data  (Attrition Bias)  All Outcomes | Criteria for a judgement of ‘Low risk’ of bias   - No missing outcome data; - Reasons for missing outcome data unlikely to be related to true outcome (for survival data, censoring unlikely to be introducing bias); - Missing outcome data balanced in numbers across intervention groups, with similar reasons for missing data across groups; - For dichotomous outcome data, the proportion of missing outcomes compared with observed event risk not enough to have a clinically relevant impact on the intervention effect estimate; - For continuous outcome data, plausible effect size (difference in means or standardized difference in means) among missing outcomes not enough to have a clinically relevant impact on observed effect size; - Missing data have been imputed using appropriate methods.   Criteria for the judgement of ‘High risk’ of bias   - Reason for missing outcome data likely to be related to true outcome, with either imbalance in numbers or reasons for missing data across intervention groups; - For dichotomous outcome data, the proportion of missing outcomes compared with observed event risk enough to induce clinically relevant bias in intervention effect estimate; - For continuous outcome data, plausible effect size (difference in means or standardized difference in means) among missing outcomes enough to induce clinically relevant bias in observed effect size; - ‘As-treated’ analysis done with substantial departure of the intervention received from that assigned at randomization; - Potentially inappropriate application of simple imputation.   Criteria for the judgement of ‘Unclear risk’ of bias   - Insufficient information to permit judgement of ‘Low risk’ or ‘High risk’; - The study did not address this outcome | Low risk | Comment: No ITT analysis was carried out, however it appears no participants dropped out. All participants attended the final study visit. |
| Selective Reporting (Reporting Bias) | Criteria for a judgement of ‘Low risk’ of bias   - The study protocol is available and all of the study’s pre-specified (primary and secondary) outcomes that are of interest in the review have been reported in the pre-specified way; - The study protocol is not available but it is clear that the published reports include all expected outcomes, including those that were pre-specified (convincing text of this nature may be uncommon).   Criteria for the judgement of ‘High risk’ of bias   - Not all of the study’s pre-specified primary outcomes have been reported; - One or more primary outcomes is reported using measurements, analysis methods or subsets of the data (e.g. subscales) that were not pre-specified; - One or more reported primary outcomes were not pre-specified (unless clear justification for their reporting is provided, such as an unexpected adverse effect); - One or more outcomes of interest in the review are reported incompletely so that they cannot be entered in a meta-analysis; - The study report fails to include results for a key outcome that would be expected to have been reported for such a study   Criteria for the judgement of ‘Unclear risk’ of bias  Insufficient information to permit judgement of ‘Low risk’ or ‘High risk’. It is likely that the majority of studies will fall into this category. | High risk | Comments: A study protocol is not available therefore, unable to determine if all of the study’s pre-specified primary outcomes were reported. |
| Other Bias | Criteria for a judgement of ‘Low risk’ of bias  Trials not published in the list of suspected predatory journals presented by Manca et al. 2017.  Criteria for the judgement of ‘High risk’ of bias  Trials published in the list of suspected predatory journals presented by Manca et al. 2017. | Low Risk | comments: The trial is not published in the list of suspected predatory journals presented by Manca et al. 2017. |

**Kurebayashi et al., 2021**

| Bias | Criteria for judging risk of bias in the Risk of Bias assessment tool | Authors’ judgement | Support for judgement |
| --- | --- | --- | --- |
|  |  |  |  |
| Random Sequence Generation  (Selection Bias) | Criteria for a judgement of ‘Low risk’ of bias  The investigators describe a random component in the sequence generation process such as:   - Referring to a random number table; - Using a computer random number generator; - Coin tossing; - Shuffling cards or envelopes; - Throwing dice; - Drawing of lots; - Minimization.   Criteria for the judgement of ‘High risk’ of bias  The investigators describe a non-random component in the sequence generation process. Usually, the description would involve some systematic, non-random approach, for example:   - Sequence generated by odd or even date of birth; - Sequence generated by some rule based on date (or day) of admission; - Sequence generated by some rule based on hospital or clinic record number.   Other non-random approaches happen much less frequently than the systematic approaches mentioned above and tend to be obvious.  They usually involve judgement or some method of non-random categorization of participants, for example:   - Allocation by judgement of the clinician; - Allocation by preference of the participant; - Allocation based on the results of a laboratory test or a series of tests; - Allocation by availability of the intervention   Criteria for the judgement of ‘Unclear risk’ of bias  Insufficient information about the sequence generation process to permit judgement of ‘Low risk’ or ‘High risk’. | Low risk | Quote: " Cluster random assignment was introduced to avoid cross‐group contamination (each ward was considered as one unit). The participants were randomly and blindly divided by ward into treatment as usual (TAU) and exercise (ratio, 1:2) groups.” |
| Allocation Concealment (Selection Bias) | Criteria for a judgement of ‘Low risk’ of bias  Participants and investigators enrolling participants could not foresee assignment because one of the following, or an equivalent method, was used to conceal allocation:   - Central allocation (including telephone, web-based and pharmacy-controlled randomization); - Sequentially numbered drug containers of identical appearance; - Sequentially numbered, opaque, sealed envelopes.   Criteria for the judgement of ‘High risk’ of bias  Participants or investigators enrolling participants could possibly foresee assignments and thus introduce selection bias, such as allocation based on:   - Using an open random allocation schedule (e.g. a list of random numbers); - Assignment envelopes were used without appropriate safeguards (e.g. if envelopes were unsealed or non­opaque or not sequentially numbered); - Alternation or rotation; - Date of birth; - Case record number; - Any other explicitly unconcealed procedure.   Criteria for the judgement of ‘Unclear risk’ of bias  Insufficient information to permit judgement of ‘Low risk’ or ‘High risk’. | Unclear risk | Quote: “All managers did not open the envelope until they selected one, which indicated the allocation concealment mechanism.”  Not clear how it was concealed |
| Blinding of Participants And Personnel  (Performance Bias)  All Outcomes | Criteria for a judgement of ‘Low risk’ of bias   - No blinding or incomplete blinding, but the review authors judge that the outcome is not likely to be influenced by lack of blinding; - Blinding of participants and key study personnel ensured, and unlikely that the blinding could have been broken.   Criteria for the judgement of ‘High risk’ of bias   - No blinding or incomplete blinding, and the outcome is likely to be influenced by lack of blinding; - Blinding of key study participants and personnel attempted, but likely that the blinding could have been broken, and the outcome is likely to be influenced by lack of blinding.   Criteria for the judgement of ‘Unclear risk’ of bias   - Insufficient information to permit judgement of ‘Low risk’ or ‘High risk’; - The study did not address this outcome | Low risk | Quote: “The participants in both groups  were blinded to the groups to which they were assigned.” |
| Blinding of Outcome Assessment  (Detection Bias)  All Outcomes | Criteria for a judgement of ‘Low risk’ of bias   - No blinding of outcome assessment, but the review authors judge that the outcome measurement is not likely to be influenced by lack of blinding; - Blinding of outcome assessment ensured, and unlikely that the blinding could have been broken.   Criteria for the judgement of ‘High risk’ of bias   - No blinding of outcome assessment, and the outcome measurement is likely to be influenced by lack of blinding; - Blinding of outcome assessment, but likely that the blinding could have been broken, and the outcome measurement is likely to be influenced by lack of blinding.   Criteria for the judgement of ‘Unclear risk’ of bias   - Insufficient information to permit judgement of ‘Low risk’ or ‘High risk’; - The study did not address this outcome | High risk | Comment: No comment in the manuscript |
| Incomplete Outcome Data  (Attrition Bias)  All Outcomes | Criteria for a judgement of ‘Low risk’ of bias   - No missing outcome data; - Reasons for missing outcome data unlikely to be related to true outcome (for survival data, censoring unlikely to be introducing bias); - Missing outcome data balanced in numbers across intervention groups, with similar reasons for missing data across groups; - For dichotomous outcome data, the proportion of missing outcomes compared with observed event risk not enough to have a clinically relevant impact on the intervention effect estimate; - For continuous outcome data, plausible effect size (difference in means or standardized difference in means) among missing outcomes not enough to have a clinically relevant impact on observed effect size; - Missing data have been imputed using appropriate methods.   Criteria for the judgement of ‘High risk’ of bias   - Reason for missing outcome data likely to be related to true outcome, with either imbalance in numbers or reasons for missing data across intervention groups; - For dichotomous outcome data, the proportion of missing outcomes compared with observed event risk enough to induce clinically relevant bias in intervention effect estimate; - For continuous outcome data, plausible effect size (difference in means or standardized difference in means) among missing outcomes enough to induce clinically relevant bias in observed effect size; - ‘As-treated’ analysis done with substantial departure of the intervention received from that assigned at randomization; - Potentially inappropriate application of simple imputation.   Criteria for the judgement of ‘Unclear risk’ of bias   - Insufficient information to permit judgement of ‘Low risk’ or ‘High risk’; - The study did not address this outcome | Low risk | Comment: Groups were skewed and the sample size was low, but all participants were included in the analysis |
| Selective Reporting (Reporting Bias) | Criteria for a judgement of ‘Low risk’ of bias   - The study protocol is available and all of the study’s pre-specified (primary and secondary) outcomes that are of interest in the review have been reported in the pre-specified way; - The study protocol is not available but it is clear that the published reports include all expected outcomes, including those that were pre-specified (convincing text of this nature may be uncommon).   Criteria for the judgement of ‘High risk’ of bias   - Not all of the study’s pre-specified primary outcomes have been reported; - One or more primary outcomes is reported using measurements, analysis methods or subsets of the data (e.g. subscales) that were not pre-specified; - One or more reported primary outcomes were not pre-specified (unless clear justification for their reporting is provided, such as an unexpected adverse effect); - One or more outcomes of interest in the review are reported incompletely so that they cannot be entered in a meta-analysis; - The study report fails to include results for a key outcome that would be expected to have been reported for such a study   Criteria for the judgement of ‘Unclear risk’ of bias  Insufficient information to permit judgement of ‘Low risk’ or ‘High risk’. It is likely that the majority of studies will fall into this category. | High risk | Comments: A study protocol is not available therefore, unable to determine if all of the study’s pre-specified primary outcomes were reported. |
| Other Bias | Criteria for a judgement of ‘Low risk’ of bias  Trials not published in the list of suspected predatory journals presented by Manca et al. 2017.  Criteria for the judgement of ‘High risk’ of bias  Trials published in the list of suspected predatory journals presented by Manca et al. 2017. | Low Risk | comments: The trial is not published in the list of suspected predatory journals presented by Manca et al. 2017. |

**Kwon et al., 2006**

| Bias | Criteria for judging risk of bias in the Risk of Bias assessment tool | Authors’ judgement | Support for judgement |
| --- | --- | --- | --- |
|  |  |  |  |
| Random Sequence Generation  (Selection Bias) | Criteria for a judgement of ‘Low risk’ of bias  The investigators describe a random component in the sequence generation process such as:   - Referring to a random number table; - Using a computer random number generator; - Coin tossing; - Shuffling cards or envelopes; - Throwing dice; - Drawing of lots; - Minimization.   Criteria for the judgement of ‘High risk’ of bias  The investigators describe a non-random component in the sequence generation process. Usually, the description would involve some systematic, non-random approach, for example:   - Sequence generated by odd or even date of birth; - Sequence generated by some rule based on date (or day) of admission; - Sequence generated by some rule based on hospital or clinic record number.   Other non-random approaches happen much less frequently than the systematic approaches mentioned above and tend to be obvious.  They usually involve judgement or some method of non-random categorization of participants, for example:   - Allocation by judgement of the clinician; - Allocation by preference of the participant; - Allocation based on the results of a laboratory test or a series of tests; - Allocation by availability of the intervention   Criteria for the judgement of ‘Unclear risk’ of bias  Insufficient information about the sequence generation process to permit judgement of ‘Low risk’ or ‘High risk’. | Unclear risk | Comment: No description of the random sequence generation |
| Allocation Concealment (Selection Bias) | Criteria for a judgement of ‘Low risk’ of bias  Participants and investigators enrolling participants could not foresee assignment because one of the following, or an equivalent method, was used to conceal allocation:   - Central allocation (including telephone, web-based and pharmacy-controlled randomization); - Sequentially numbered drug containers of identical appearance; - Sequentially numbered, opaque, sealed envelopes.   Criteria for the judgement of ‘High risk’ of bias  Participants or investigators enrolling participants could possibly foresee assignments and thus introduce selection bias, such as allocation based on:   - Using an open random allocation schedule (e.g. a list of random numbers); - Assignment envelopes were used without appropriate safeguards (e.g. if envelopes were unsealed or non­opaque or not sequentially numbered); - Alternation or rotation; - Date of birth; - Case record number; - Any other explicitly unconcealed procedure.   Criteria for the judgement of ‘Unclear risk’ of bias  Insufficient information to permit judgement of ‘Low risk’ or ‘High risk’. | Unclear risk | Quote: “The participants who fulfilled the inclusion/ exclusion criteria were randomly allocated to either the intervention group or the control group”  Comment: It’s not clear whether the allocation was concealed |
| Blinding of Participants And Personnel  (Performance Bias)  All Outcomes | Criteria for a judgement of ‘Low risk’ of bias   - No blinding or incomplete blinding, but the review authors judge that the outcome is not likely to be influenced by lack of blinding; - Blinding of participants and key study personnel ensured, and unlikely that the blinding could have been broken.   Criteria for the judgement of ‘High risk’ of bias   - No blinding or incomplete blinding, and the outcome is likely to be influenced by lack of blinding; - Blinding of key study participants and personnel attempted, but likely that the blinding could have been broken, and the outcome is likely to be influenced by lack of blinding.   Criteria for the judgement of ‘Unclear risk’ of bias   - Insufficient information to permit judgement of ‘Low risk’ or ‘High risk’; - The study did not address this outcome | High risk | Comment: Blinding of participants and personnel was not possible due to nature of intervention. |
| Blinding of Outcome Assessment  (Detection Bias)  All Outcomes | Criteria for a judgement of ‘Low risk’ of bias   - No blinding of outcome assessment, but the review authors judge that the outcome measurement is not likely to be influenced by lack of blinding; - Blinding of outcome assessment ensured, and unlikely that the blinding could have been broken.   Criteria for the judgement of ‘High risk’ of bias   - No blinding of outcome assessment, and the outcome measurement is likely to be influenced by lack of blinding; - Blinding of outcome assessment, but likely that the blinding could have been broken, and the outcome measurement is likely to be influenced by lack of blinding.   Criteria for the judgement of ‘Unclear risk’ of bias   - Insufficient information to permit judgement of ‘Low risk’ or ‘High risk’; - The study did not address this outcome | High risk | Comment: There was no description of blinding assessors |
| Incomplete Outcome Data  (Attrition Bias)  All Outcomes | Criteria for a judgement of ‘Low risk’ of bias   - No missing outcome data; - Reasons for missing outcome data unlikely to be related to true outcome (for survival data, censoring unlikely to be introducing bias); - Missing outcome data balanced in numbers across intervention groups, with similar reasons for missing data across groups; - For dichotomous outcome data, the proportion of missing outcomes compared with observed event risk not enough to have a clinically relevant impact on the intervention effect estimate; - For continuous outcome data, plausible effect size (difference in means or standardized difference in means) among missing outcomes not enough to have a clinically relevant impact on observed effect size; - Missing data have been imputed using appropriate methods.   Criteria for the judgement of ‘High risk’ of bias   - Reason for missing outcome data likely to be related to true outcome, with either imbalance in numbers or reasons for missing data across intervention groups; - For dichotomous outcome data, the proportion of missing outcomes compared with observed event risk enough to induce clinically relevant bias in intervention effect estimate; - For continuous outcome data, plausible effect size (difference in means or standardized difference in means) among missing outcomes enough to induce clinically relevant bias in observed effect size; - ‘As-treated’ analysis done with substantial departure of the intervention received from that assigned at randomization; - Potentially inappropriate application of simple imputation.   Criteria for the judgement of ‘Unclear risk’ of bias   - Insufficient information to permit judgement of ‘Low risk’ or ‘High risk’; - The study did not address this outcome | Low risk | Quote: “In cases which complete data regarding weight during 12 weeks were unavailable, last-observation-carried forward was used” |
| Selective Reporting (Reporting Bias) | Criteria for a judgement of ‘Low risk’ of bias   - The study protocol is available and all of the study’s pre-specified (primary and secondary) outcomes that are of interest in the review have been reported in the pre-specified way; - The study protocol is not available but it is clear that the published reports include all expected outcomes, including those that were pre-specified (convincing text of this nature may be uncommon).   Criteria for the judgement of ‘High risk’ of bias   - Not all of the study’s pre-specified primary outcomes have been reported; - One or more primary outcomes is reported using measurements, analysis methods or subsets of the data (e.g. subscales) that were not pre-specified; - One or more reported primary outcomes were not pre-specified (unless clear justification for their reporting is provided, such as an unexpected adverse effect); - One or more outcomes of interest in the review are reported incompletely so that they cannot be entered in a meta-analysis; - The study report fails to include results for a key outcome that would be expected to have been reported for such a study   Criteria for the judgement of ‘Unclear risk’ of bias  Insufficient information to permit judgement of ‘Low risk’ or ‘High risk’. It is likely that the majority of studies will fall into this category. | High risk | Comments: A study protocol is not available therefore, unable to determine if all of the study’s pre-specified primary outcomes were reported. |
| Other Bias | Criteria for a judgement of ‘Low risk’ of bias  Trials not published in the list of suspected predatory journals presented by Manca et al. 2017.  Criteria for the judgement of ‘High risk’ of bias  Trials published in the list of suspected predatory journals presented by Manca et al. 2017. | Low Risk | comments: The trial is not published in the list of suspected predatory journals presented by Manca et al. 2017. |

**Li et al., 2020**

| Bias | Criteria for judging risk of bias in the Risk of Bias assessment tool | Authors’ judgement | Support for judgement |
| --- | --- | --- | --- |
|  |  |  |  |
| Random Sequence Generation  (Selection Bias) | Criteria for a judgement of ‘Low risk’ of bias  The investigators describe a random component in the sequence generation process such as:   - Referring to a random number table; - Using a computer random number generator; - Coin tossing; - Shuffling cards or envelopes; - Throwing dice; - Drawing of lots; - Minimization.   Criteria for the judgement of ‘High risk’ of bias  The investigators describe a non-random component in the sequence generation process. Usually, the description would involve some systematic, non-random approach, for example:   - Sequence generated by odd or even date of birth; - Sequence generated by some rule based on date (or day) of admission; - Sequence generated by some rule based on hospital or clinic record number.   Other non-random approaches happen much less frequently than the systematic approaches mentioned above and tend to be obvious.  They usually involve judgement or some method of non-random categorization of participants, for example:   - Allocation by judgement of the clinician; - Allocation by preference of the participant; - Allocation based on the results of a laboratory test or a series of tests; - Allocation by availability of the intervention   Criteria for the judgement of ‘Unclear risk’ of bias  Insufficient information about the sequence generation process to permit judgement of ‘Low risk’ or ‘High risk’. | Low risk | Quote: “Patients were randomly assigned  to the Baduanjin exercise (treatment) or the brisk walking (control) groups using a random permuted block design. The types of intervention were written on a piece of paper and put in a sealed black box, and we picked one for each patient who agreed to be in the study. 30 patients were randomly allocated to the Baduanjin group, and the other 31 patients were assigned to the brisk walking control group.” |
| Allocation Concealment (Selection Bias) | Criteria for a judgement of ‘Low risk’ of bias  Participants and investigators enrolling participants could not foresee assignment because one of the following, or an equivalent method, was used to conceal allocation:   - Central allocation (including telephone, web-based and pharmacy-controlled randomization); - Sequentially numbered drug containers of identical appearance; - Sequentially numbered, opaque, sealed envelopes.   Criteria for the judgement of ‘High risk’ of bias  Participants or investigators enrolling participants could possibly foresee assignments and thus introduce selection bias, such as allocation based on:   - Using an open random allocation schedule (e.g. a list of random numbers); - Assignment envelopes were used without appropriate safeguards (e.g. if envelopes were unsealed or non­opaque or not sequentially numbered); - Alternation or rotation; - Date of birth; - Case record number; - Any other explicitly unconcealed procedure.   Criteria for the judgement of ‘Unclear risk’ of bias  Insufficient information to permit judgement of ‘Low risk’ or ‘High risk’. | Low risk | Quote: “Patients were randomly assigned  to the Baduanjin exercise (treatment) or the brisk walking (control) groups using a random permuted block design. The types of intervention were written on a piece of paper and put in a sealed black box, and we picked one for each patient who agreed to be in the study. 30 patients were randomly allocated to the Baduanjin group, and the other 31 patients were assigned to the brisk walking control group.” |
| Blinding of Participants And Personnel  (Performance Bias)  All Outcomes | Criteria for a judgement of ‘Low risk’ of bias   - No blinding or incomplete blinding, but the review authors judge that the outcome is not likely to be influenced by lack of blinding; - Blinding of participants and key study personnel ensured, and unlikely that the blinding could have been broken.   Criteria for the judgement of ‘High risk’ of bias   - No blinding or incomplete blinding, and the outcome is likely to be influenced by lack of blinding; - Blinding of key study participants and personnel attempted, but likely that the blinding could have been broken, and the outcome is likely to be influenced by lack of blinding.   Criteria for the judgement of ‘Unclear risk’ of bias   - Insufficient information to permit judgement of ‘Low risk’ or ‘High risk’; - The study did not address this outcome | High risk | Quote: “All participants were given a complete description of the study before  they provided written informed consent”  Comment: Blinding of participants and personnel was not possible due to nature of intervention. |
| Blinding of Outcome Assessment  (Detection Bias)  All Outcomes | Criteria for a judgement of ‘Low risk’ of bias   - No blinding of outcome assessment, but the review authors judge that the outcome measurement is not likely to be influenced by lack of blinding; - Blinding of outcome assessment ensured, and unlikely that the blinding could have been broken.   Criteria for the judgement of ‘High risk’ of bias   - No blinding of outcome assessment, and the outcome measurement is likely to be influenced by lack of blinding; - Blinding of outcome assessment, but likely that the blinding could have been broken, and the outcome measurement is likely to be influenced by lack of blinding.   Criteria for the judgement of ‘Unclear risk’ of bias   - Insufficient information to permit judgement of ‘Low risk’ or ‘High risk’; - The study did not address this outcome | Low risk | Quote: “The neurocognitive tests of all patients were administered by Dr. Luo, who was blinded to the participants’ group assignment” |
| Incomplete Outcome Data  (Attrition Bias)  All Outcomes | Criteria for a judgement of ‘Low risk’ of bias   - No missing outcome data; - Reasons for missing outcome data unlikely to be related to true outcome (for survival data, censoring unlikely to be introducing bias); - Missing outcome data balanced in numbers across intervention groups, with similar reasons for missing data across groups; - For dichotomous outcome data, the proportion of missing outcomes compared with observed event risk not enough to have a clinically relevant impact on the intervention effect estimate; - For continuous outcome data, plausible effect size (difference in means or standardized difference in means) among missing outcomes not enough to have a clinically relevant impact on observed effect size; - Missing data have been imputed using appropriate methods.   Criteria for the judgement of ‘High risk’ of bias   - Reason for missing outcome data likely to be related to true outcome, with either imbalance in numbers or reasons for missing data across intervention groups; - For dichotomous outcome data, the proportion of missing outcomes compared with observed event risk enough to induce clinically relevant bias in intervention effect estimate; - For continuous outcome data, plausible effect size (difference in means or standardized difference in means) among missing outcomes enough to induce clinically relevant bias in observed effect size; - ‘As-treated’ analysis done with substantial departure of the intervention received from that assigned at randomization; - Potentially inappropriate application of simple imputation.   Criteria for the judgement of ‘Unclear risk’ of bias   - Insufficient information to permit judgement of ‘Low risk’ or ‘High risk’; - The study did not address this outcome | Low risk | Quote: “No patients dropped out during this study.” |
| Selective Reporting (Reporting Bias) | Criteria for a judgement of ‘Low risk’ of bias   - The study protocol is available and all of the study’s pre-specified (primary and secondary) outcomes that are of interest in the review have been reported in the pre-specified way; - The study protocol is not available but it is clear that the published reports include all expected outcomes, including those that were pre-specified (convincing text of this nature may be uncommon).   Criteria for the judgement of ‘High risk’ of bias   - Not all of the study’s pre-specified primary outcomes have been reported; - One or more primary outcomes is reported using measurements, analysis methods or subsets of the data (e.g. subscales) that were not pre-specified; - One or more reported primary outcomes were not pre-specified (unless clear justification for their reporting is provided, such as an unexpected adverse effect); - One or more outcomes of interest in the review are reported incompletely so that they cannot be entered in a meta-analysis; - The study report fails to include results for a key outcome that would be expected to have been reported for such a study   Criteria for the judgement of ‘Unclear risk’ of bias  Insufficient information to permit judgement of ‘Low risk’ or ‘High risk’. It is likely that the majority of studies will fall into this category. | High risk | Comments: A study protocol is not available therefore, unable to determine if all of the study’s pre-specified primary outcomes were reported. |
| Other Bias | Criteria for a judgement of ‘Low risk’ of bias  Trials not published in the list of suspected predatory journals presented by Manca et al. 2017.  Criteria for the judgement of ‘High risk’ of bias  Trials published in the list of suspected predatory journals presented by Manca et al. 2017. | Low Risk | comments: The trial is not published in the list of suspected predatory journals presented by Manca et al. 2017. |

**Loh et al., 2016**

| Bias | Criteria for judging risk of bias in the Risk of Bias assessment tool | Authors’ judgement | Support for judgement |
| --- | --- | --- | --- |
|  |  |  |  |
| Random Sequence Generation  (Selection Bias) | Criteria for a judgement of ‘Low risk’ of bias  The investigators describe a random component in the sequence generation process such as:   - Referring to a random number table; - Using a computer random number generator; - Coin tossing; - Shuffling cards or envelopes; - Throwing dice; - Drawing of lots; - Minimization.   Criteria for the judgement of ‘High risk’ of bias  The investigators describe a non-random component in the sequence generation process. Usually, the description would involve some systematic, non-random approach, for example:   - Sequence generated by odd or even date of birth; - Sequence generated by some rule based on date (or day) of admission; - Sequence generated by some rule based on hospital or clinic record number.   Other non-random approaches happen much less frequently than the systematic approaches mentioned above and tend to be obvious.  They usually involve judgement or some method of non-random categorization of participants, for example:   - Allocation by judgement of the clinician; - Allocation by preference of the participant; - Allocation based on the results of a laboratory test or a series of tests; - Allocation by availability of the intervention   Criteria for the judgement of ‘Unclear risk’ of bias  Insufficient information about the sequence generation process to permit judgement of ‘Low risk’ or ‘High risk’. | High risk | Quote: “Participants were randomly assigned to either the intervention or control (treatment-as-usual) groups using a computerized random number generator. Randomization was done in blocks of two to ensure equal number of  patients in each group.” |
| Allocation Concealment (Selection Bias) | Criteria for a judgement of ‘Low risk’ of bias  Participants and investigators enrolling participants could not foresee assignment because one of the following, or an equivalent method, was used to conceal allocation:   - Central allocation (including telephone, web-based and pharmacy-controlled randomization); - Sequentially numbered drug containers of identical appearance; - Sequentially numbered, opaque, sealed envelopes.   Criteria for the judgement of ‘High risk’ of bias  Participants or investigators enrolling participants could possibly foresee assignments and thus introduce selection bias, such as allocation based on:   - Using an open random allocation schedule (e.g. a list of random numbers); - Assignment envelopes were used without appropriate safeguards (e.g. if envelopes were unsealed or non­opaque or not sequentially numbered); - Alternation or rotation; - Date of birth; - Case record number; - Any other explicitly unconcealed procedure.   Criteria for the judgement of ‘Unclear risk’ of bias  Insufficient information to permit judgement of ‘Low risk’ or ‘High risk’. | Low risk | Quote: “Allocation of participants were concealed from the primary investigators of the study as randomization codes were kept by a research assistant not involved in this study and further concealed by sealing codes in envelopes.” |
| Blinding of Participants And Personnel  (Performance Bias)  All Outcomes | Criteria for a judgement of ‘Low risk’ of bias   - No blinding or incomplete blinding, but the review authors judge that the outcome is not likely to be influenced by lack of blinding; - Blinding of participants and key study personnel ensured, and unlikely that the blinding could have been broken.   Criteria for the judgement of ‘High risk’ of bias   - No blinding or incomplete blinding, and the outcome is likely to be influenced by lack of blinding; - Blinding of key study participants and personnel attempted, but likely that the blinding could have been broken, and the outcome is likely to be influenced by lack of blinding.   Criteria for the judgement of ‘Unclear risk’ of bias   - Insufficient information to permit judgement of ‘Low risk’ or ‘High risk’; - The study did not address this outcome | High risk | Quote: “patients are non-blinded to the intervention that they were receiving”  Comment: Blinding of participants and personnel was not possible due to nature of intervention. |
| Blinding of Outcome Assessment  (Detection Bias)  All Outcomes | Criteria for a judgement of ‘Low risk’ of bias   - No blinding of outcome assessment, but the review authors judge that the outcome measurement is not likely to be influenced by lack of blinding; - Blinding of outcome assessment ensured, and unlikely that the blinding could have been broken.   Criteria for the judgement of ‘High risk’ of bias   - No blinding of outcome assessment, and the outcome measurement is likely to be influenced by lack of blinding; - Blinding of outcome assessment, but likely that the blinding could have been broken, and the outcome measurement is likely to be influenced by lack of blinding.   Criteria for the judgement of ‘Unclear risk’ of bias   - Insufficient information to permit judgement of ‘Low risk’ or ‘High risk’; - The study did not address this outcome | Unclear risk | Comment: not statement was made on whether the assessor was blinded |
| Incomplete Outcome Data  (Attrition Bias)  All Outcomes | Criteria for a judgement of ‘Low risk’ of bias   - No missing outcome data; - Reasons for missing outcome data unlikely to be related to true outcome (for survival data, censoring unlikely to be introducing bias); - Missing outcome data balanced in numbers across intervention groups, with similar reasons for missing data across groups; - For dichotomous outcome data, the proportion of missing outcomes compared with observed event risk not enough to have a clinically relevant impact on the intervention effect estimate; - For continuous outcome data, plausible effect size (difference in means or standardized difference in means) among missing outcomes not enough to have a clinically relevant impact on observed effect size; - Missing data have been imputed using appropriate methods.   Criteria for the judgement of ‘High risk’ of bias   - Reason for missing outcome data likely to be related to true outcome, with either imbalance in numbers or reasons for missing data across intervention groups; - For dichotomous outcome data, the proportion of missing outcomes compared with observed event risk enough to induce clinically relevant bias in intervention effect estimate; - For continuous outcome data, plausible effect size (difference in means or standardized difference in means) among missing outcomes enough to induce clinically relevant bias in observed effect size; - ‘As-treated’ analysis done with substantial departure of the intervention received from that assigned at randomization; - Potentially inappropriate application of simple imputation.   Criteria for the judgement of ‘Unclear risk’ of bias   - Insufficient information to permit judgement of ‘Low risk’ or ‘High risk’; - The study did not address this outcome | High risk | Quote: “In the intervention group, four (7.8%) participants had dropped out of the study”  Comment: no ITT analyses were described. Follow up data analysis was conducted on 48 participants rather than 52, suggesting that missing data was excluded from the analysis. |
| Selective Reporting (Reporting Bias) | Criteria for a judgement of ‘Low risk’ of bias   - The study protocol is available and all of the study’s pre-specified (primary and secondary) outcomes that are of interest in the review have been reported in the pre-specified way; - The study protocol is not available but it is clear that the published reports include all expected outcomes, including those that were pre-specified (convincing text of this nature may be uncommon).   Criteria for the judgement of ‘High risk’ of bias   - Not all of the study’s pre-specified primary outcomes have been reported; - One or more primary outcomes is reported using measurements, analysis methods or subsets of the data (e.g. subscales) that were not pre-specified; - One or more reported primary outcomes were not pre-specified (unless clear justification for their reporting is provided, such as an unexpected adverse effect); - One or more outcomes of interest in the review are reported incompletely so that they cannot be entered in a meta-analysis; - The study report fails to include results for a key outcome that would be expected to have been reported for such a study   Criteria for the judgement of ‘Unclear risk’ of bias  Insufficient information to permit judgement of ‘Low risk’ or ‘High risk’. It is likely that the majority of studies will fall into this category. | High risk | Comments: A study protocol is not available therefore, unable to determine if all of the study’s pre-specified primary outcomes were reported. |
| Other Bias | Criteria for a judgement of ‘Low risk’ of bias  Trials not published in the list of suspected predatory journals presented by Manca et al. 2017.  Criteria for the judgement of ‘High risk’ of bias  Trials published in the list of suspected predatory journals presented by Manca et al. 2017. | Low Risk | comments: The trial is not published in the list of suspected predatory journals presented by Manca et al. 2017. |

**Manjunath et al., 2013**

| Bias | Criteria for judging risk of bias in the Risk of Bias assessment tool | Authors’ judgement | Support for judgement |
| --- | --- | --- | --- |
|  |  |  |  |
| Random Sequence Generation  (Selection Bias) | Criteria for a judgement of ‘Low risk’ of bias  The investigators describe a random component in the sequence generation process such as:   - Referring to a random number table; - Using a computer random number generator; - Coin tossing; - Shuffling cards or envelopes; - Throwing dice; - Drawing of lots; - Minimization.   Criteria for the judgement of ‘High risk’ of bias  The investigators describe a non-random component in the sequence generation process. Usually, the description would involve some systematic, non-random approach, for example:   - Sequence generated by odd or even date of birth; - Sequence generated by some rule based on date (or day) of admission; - Sequence generated by some rule based on hospital or clinic record number.   Other non-random approaches happen much less frequently than the systematic approaches mentioned above and tend to be obvious.  They usually involve judgement or some method of non-random categorization of participants, for example:   - Allocation by judgement of the clinician; - Allocation by preference of the participant; - Allocation based on the results of a laboratory test or a series of tests; - Allocation by availability of the intervention   Criteria for the judgement of ‘Unclear risk’ of bias  Insufficient information about the sequence generation process to permit judgement of ‘Low risk’ or ‘High risk’. | Unclear risk | Quote: “A randomization table was generated for 90 patients to have equal representation of yoga or exercise as an add-on/complementary treatment. However, the recruitment  had to be stopped after 88 patients when 44 patients each had been randomized into yoga and exercise groups”  Not a clear description of the randomization table |
| Allocation Concealment (Selection Bias) | Criteria for a judgement of ‘Low risk’ of bias  Participants and investigators enrolling participants could not foresee assignment because one of the following, or an equivalent method, was used to conceal allocation:   - Central allocation (including telephone, web-based and pharmacy-controlled randomization); - Sequentially numbered drug containers of identical appearance; - Sequentially numbered, opaque, sealed envelopes.   Criteria for the judgement of ‘High risk’ of bias  Participants or investigators enrolling participants could possibly foresee assignments and thus introduce selection bias, such as allocation based on:   - Using an open random allocation schedule (e.g. a list of random numbers); - Assignment envelopes were used without appropriate safeguards (e.g. if envelopes were unsealed or non­opaque or not sequentially numbered); - Alternation or rotation; - Date of birth; - Case record number; - Any other explicitly unconcealed procedure.   Criteria for the judgement of ‘Unclear risk’ of bias  Insufficient information to permit judgement of ‘Low risk’ or ‘High risk’. | Unclear risk | Comment: It’s not clear whether the allocation was concealed |
| Blinding of Participants And Personnel  (Performance Bias)  All Outcomes | Criteria for a judgement of ‘Low risk’ of bias   - No blinding or incomplete blinding, but the review authors judge that the outcome is not likely to be influenced by lack of blinding; - Blinding of participants and key study personnel ensured, and unlikely that the blinding could have been broken.   Criteria for the judgement of ‘High risk’ of bias   - No blinding or incomplete blinding, and the outcome is likely to be influenced by lack of blinding; - Blinding of key study participants and personnel attempted, but likely that the blinding could have been broken, and the outcome is likely to be influenced by lack of blinding.   Criteria for the judgement of ‘Unclear risk’ of bias   - Insufficient information to permit judgement of ‘Low risk’ or ‘High risk’; - The study did not address this outcome | High risk | Comment: Blinding of participants and personnel was not possible due to nature of intervention. |
| Blinding of Outcome Assessment  (Detection Bias)  All Outcomes | Criteria for a judgement of ‘Low risk’ of bias   - No blinding of outcome assessment, but the review authors judge that the outcome measurement is not likely to be influenced by lack of blinding; - Blinding of outcome assessment ensured, and unlikely that the blinding could have been broken.   Criteria for the judgement of ‘High risk’ of bias   - No blinding of outcome assessment, and the outcome measurement is likely to be influenced by lack of blinding; - Blinding of outcome assessment, but likely that the blinding could have been broken, and the outcome measurement is likely to be influenced by lack of blinding.   Criteria for the judgement of ‘Unclear risk’ of bias   - Insufficient information to permit judgement of ‘Low risk’ or ‘High risk’; - The study did not address this outcome | Low risk | Quote: “The rater on this scale was uninvolved in the treatment and therefore blind to group status” |
| Incomplete Outcome Data  (Attrition Bias)  All Outcomes | Criteria for a judgement of ‘Low risk’ of bias   - No missing outcome data; - Reasons for missing outcome data unlikely to be related to true outcome (for survival data, censoring unlikely to be introducing bias); - Missing outcome data balanced in numbers across intervention groups, with similar reasons for missing data across groups; - For dichotomous outcome data, the proportion of missing outcomes compared with observed event risk not enough to have a clinically relevant impact on the intervention effect estimate; - For continuous outcome data, plausible effect size (difference in means or standardized difference in means) among missing outcomes not enough to have a clinically relevant impact on observed effect size; - Missing data have been imputed using appropriate methods.   Criteria for the judgement of ‘High risk’ of bias   - Reason for missing outcome data likely to be related to true outcome, with either imbalance in numbers or reasons for missing data across intervention groups; - For dichotomous outcome data, the proportion of missing outcomes compared with observed event risk enough to induce clinically relevant bias in intervention effect estimate; - For continuous outcome data, plausible effect size (difference in means or standardized difference in means) among missing outcomes enough to induce clinically relevant bias in observed effect size; - ‘As-treated’ analysis done with substantial departure of the intervention received from that assigned at randomization; - Potentially inappropriate application of simple imputation.   Criteria for the judgement of ‘Unclear risk’ of bias   - Insufficient information to permit judgement of ‘Low risk’ or ‘High risk’; - The study did not address this outcome | High risk | Comment: 28 participants dropped out. An ITT was not completed, however, group differences between those that dropped out and the other participants was assessed. There were statistically significant differences. |
| Selective Reporting (Reporting Bias) | Criteria for a judgement of ‘Low risk’ of bias   - The study protocol is available and all of the study’s pre-specified (primary and secondary) outcomes that are of interest in the review have been reported in the pre-specified way; - The study protocol is not available but it is clear that the published reports include all expected outcomes, including those that were pre-specified (convincing text of this nature may be uncommon).   Criteria for the judgement of ‘High risk’ of bias   - Not all of the study’s pre-specified primary outcomes have been reported; - One or more primary outcomes is reported using measurements, analysis methods or subsets of the data (e.g. subscales) that were not pre-specified; - One or more reported primary outcomes were not pre-specified (unless clear justification for their reporting is provided, such as an unexpected adverse effect); - One or more outcomes of interest in the review are reported incompletely so that they cannot be entered in a meta-analysis; - The study report fails to include results for a key outcome that would be expected to have been reported for such a study   Criteria for the judgement of ‘Unclear risk’ of bias  Insufficient information to permit judgement of ‘Low risk’ or ‘High risk’. It is likely that the majority of studies will fall into this category. | High risk | Comments: A study protocol is not available therefore, unable to determine if all of the study’s pre-specified primary outcomes were reported. |
| Other Bias | Criteria for a judgement of ‘Low risk’ of bias  Trials not published in the list of suspected predatory journals presented by Manca et al. 2017.  Criteria for the judgement of ‘High risk’ of bias  Trials published in the list of suspected predatory journals presented by Manca et al. 2017. | Low Risk | comments: The trial is not published in the list of suspected predatory journals presented by Manca et al. 2017. |

**Methapatara et al. 2011**

| Bias | Criteria for judging risk of bias in the Risk of Bias assessment tool | Authors’ judgement | Support for judgement |
| --- | --- | --- | --- |
|  |  |  |  |
| Random Sequence Generation  (Selection Bias) | Criteria for a judgement of ‘Low risk’ of bias  The investigators describe a random component in the sequence generation process such as:   - Referring to a random number table; - Using a computer random number generator; - Coin tossing; - Shuffling cards or envelopes; - Throwing dice; - Drawing of lots; - Minimization.   Criteria for the judgement of ‘High risk’ of bias  The investigators describe a non-random component in the sequence generation process. Usually, the description would involve some systematic, non-random approach, for example:   - Sequence generated by odd or even date of birth; - Sequence generated by some rule based on date (or day) of admission; - Sequence generated by some rule based on hospital or clinic record number.   Other non-random approaches happen much less frequently than the systematic approaches mentioned above and tend to be obvious.  They usually involve judgement or some method of non-random categorization of participants, for example:   - Allocation by judgement of the clinician; - Allocation by preference of the participant; - Allocation based on the results of a laboratory test or a series of tests; - Allocation by availability of the intervention   Criteria for the judgement of ‘Unclear risk’ of bias  Insufficient information about the sequence generation process to permit judgement of ‘Low risk’ or ‘High risk’. | Low risk | Quote: “Stratified randomization was applied for allocating participants into intervention and control groups. Participants were divided into those taking antipsychotics with a high propensity to induce weight gain (i.e. clozapine, olanzapine) and those without a high propensity to induce weight gain (all other antipsychotics). The allocation ratio for being an intervention participant or a control was 1:1.” |
| Allocation Concealment (Selection Bias) | Criteria for a judgement of ‘Low risk’ of bias  Participants and investigators enrolling participants could not foresee assignment because one of the following, or an equivalent method, was used to conceal allocation:   - Central allocation (including telephone, web-based and pharmacy-controlled randomization); - Sequentially numbered drug containers of identical appearance; - Sequentially numbered, opaque, sealed envelopes.   Criteria for the judgement of ‘High risk’ of bias  Participants or investigators enrolling participants could possibly foresee assignments and thus introduce selection bias, such as allocation based on:   - Using an open random allocation schedule (e.g. a list of random numbers); - Assignment envelopes were used without appropriate safeguards (e.g. if envelopes were unsealed or non­opaque or not sequentially numbered); - Alternation or rotation; - Date of birth; - Case record number; - Any other explicitly unconcealed procedure.   Criteria for the judgement of ‘Unclear risk’ of bias  Insufficient information to permit judgement of ‘Low risk’ or ‘High risk’. | Low risk | Quote: “Random allocation sequences were generated by the computer. The random number indicating intervention  or control was kept in an opaque and sealed envelope. The envelope was opened after the baseline assessment of each participant had been completed. No blindness was applied in this study.” |
| Blinding of Participants And Personnel  (Performance Bias)  All Outcomes | Criteria for a judgement of ‘Low risk’ of bias   - No blinding or incomplete blinding, but the review authors judge that the outcome is not likely to be influenced by lack of blinding; - Blinding of participants and key study personnel ensured, and unlikely that the blinding could have been broken.   Criteria for the judgement of ‘High risk’ of bias   - No blinding or incomplete blinding, and the outcome is likely to be influenced by lack of blinding; - Blinding of key study participants and personnel attempted, but likely that the blinding could have been broken, and the outcome is likely to be influenced by lack of blinding.   Criteria for the judgement of ‘Unclear risk’ of bias   - Insufficient information to permit judgement of ‘Low risk’ or ‘High risk’; - The study did not address this outcome | High risk | Quote: “No blindness was applied in this study.” |
| Blinding of Outcome Assessment  (Detection Bias)  All Outcomes | Criteria for a judgement of ‘Low risk’ of bias   - No blinding of outcome assessment, but the review authors judge that the outcome measurement is not likely to be influenced by lack of blinding; - Blinding of outcome assessment ensured, and unlikely that the blinding could have been broken.   Criteria for the judgement of ‘High risk’ of bias   - No blinding of outcome assessment, and the outcome measurement is likely to be influenced by lack of blinding; - Blinding of outcome assessment, but likely that the blinding could have been broken, and the outcome measurement is likely to be influenced by lack of blinding.   Criteria for the judgement of ‘Unclear risk’ of bias   - Insufficient information to permit judgement of ‘Low risk’ or ‘High risk’; - The study did not address this outcome | High risk | Quote: “No blindness was applied in this study.” |
| Incomplete Outcome Data  (Attrition Bias)  All Outcomes | Criteria for a judgement of ‘Low risk’ of bias   - No missing outcome data; - Reasons for missing outcome data unlikely to be related to true outcome (for survival data, censoring unlikely to be introducing bias); - Missing outcome data balanced in numbers across intervention groups, with similar reasons for missing data across groups; - For dichotomous outcome data, the proportion of missing outcomes compared with observed event risk not enough to have a clinically relevant impact on the intervention effect estimate; - For continuous outcome data, plausible effect size (difference in means or standardized difference in means) among missing outcomes not enough to have a clinically relevant impact on observed effect size; - Missing data have been imputed using appropriate methods.   Criteria for the judgement of ‘High risk’ of bias   - Reason for missing outcome data likely to be related to true outcome, with either imbalance in numbers or reasons for missing data across intervention groups; - For dichotomous outcome data, the proportion of missing outcomes compared with observed event risk enough to induce clinically relevant bias in intervention effect estimate; - For continuous outcome data, plausible effect size (difference in means or standardized difference in means) among missing outcomes enough to induce clinically relevant bias in observed effect size; - ‘As-treated’ analysis done with substantial departure of the intervention received from that assigned at randomization; - Potentially inappropriate application of simple imputation.   Criteria for the judgement of ‘Unclear risk’ of bias   - Insufficient information to permit judgement of ‘Low risk’ or ‘High risk’; - The study did not address this outcome | Low risk | Quote: “Last observation carried forward analysis was applied for the bodyweight,  BMI and waist circumference” |
| Selective Reporting (Reporting Bias) | Criteria for a judgement of ‘Low risk’ of bias   - The study protocol is available and all of the study’s pre-specified (primary and secondary) outcomes that are of interest in the review have been reported in the pre-specified way; - The study protocol is not available but it is clear that the published reports include all expected outcomes, including those that were pre-specified (convincing text of this nature may be uncommon).   Criteria for the judgement of ‘High risk’ of bias   - Not all of the study’s pre-specified primary outcomes have been reported; - One or more primary outcomes is reported using measurements, analysis methods or subsets of the data (e.g. subscales) that were not pre-specified; - One or more reported primary outcomes were not pre-specified (unless clear justification for their reporting is provided, such as an unexpected adverse effect); - One or more outcomes of interest in the review are reported incompletely so that they cannot be entered in a meta-analysis; - The study report fails to include results for a key outcome that would be expected to have been reported for such a study   Criteria for the judgement of ‘Unclear risk’ of bias  Insufficient information to permit judgement of ‘Low risk’ or ‘High risk’. It is likely that the majority of studies will fall into this category. | High risk | Comments: A study protocol is not available therefore, unable to determine if all of the study’s pre-specified primary outcomes were reported. |
| Other Bias | Criteria for a judgement of ‘Low risk’ of bias  Trials not published in the list of suspected predatory journals presented by Manca et al. 2017.  Criteria for the judgement of ‘High risk’ of bias  Trials published in the list of suspected predatory journals presented by Manca et al. 2017. | Low Risk | comments: The trial is not published in the list of suspected predatory journals presented by Manca et al. 2017. |

**Oertel-Knochel et al., 2014**

| Bias | Criteria for judging risk of bias in the Risk of Bias assessment tool | Authors’ judgement | Support for judgement |
| --- | --- | --- | --- |
|  |  |  |  |
| Random Sequence Generation  (Selection Bias) | Criteria for a judgement of ‘Low risk’ of bias  The investigators describe a random component in the sequence generation process such as:   - Referring to a random number table; - Using a computer random number generator; - Coin tossing; - Shuffling cards or envelopes; - Throwing dice; - Drawing of lots; - Minimization.   Criteria for the judgement of ‘High risk’ of bias  The investigators describe a non-random component in the sequence generation process. Usually, the description would involve some systematic, non-random approach, for example:   - Sequence generated by odd or even date of birth; - Sequence generated by some rule based on date (or day) of admission; - Sequence generated by some rule based on hospital or clinic record number.   Other non-random approaches happen much less frequently than the systematic approaches mentioned above and tend to be obvious.  They usually involve judgement or some method of non-random categorization of participants, for example:   - Allocation by judgement of the clinician; - Allocation by preference of the participant; - Allocation based on the results of a laboratory test or a series of tests; - Allocation by availability of the intervention   Criteria for the judgement of ‘Unclear risk’ of bias  Insufficient information about the sequence generation process to permit judgement of ‘Low risk’ or ‘High risk’. | Low risk | Quote: “The recruitment and randomization process has been  done as follows: the different interventions were numbered (1 = exercise, 2 = relaxation, 3 = waiting control). All participants were informed that they will be randomly distributed to one of the possible interventions… using Microsoft Excel software” |
| Allocation Concealment (Selection Bias) | Criteria for a judgement of ‘Low risk’ of bias  Participants and investigators enrolling participants could not foresee assignment because one of the following, or an equivalent method, was used to conceal allocation:   - Central allocation (including telephone, web-based and pharmacy-controlled randomization); - Sequentially numbered drug containers of identical appearance; - Sequentially numbered, opaque, sealed envelopes.   Criteria for the judgement of ‘High risk’ of bias  Participants or investigators enrolling participants could possibly foresee assignments and thus introduce selection bias, such as allocation based on:   - Using an open random allocation schedule (e.g. a list of random numbers); - Assignment envelopes were used without appropriate safeguards (e.g. if envelopes were unsealed or non­opaque or not sequentially numbered); - Alternation or rotation; - Date of birth; - Case record number; - Any other explicitly unconcealed procedure.   Criteria for the judgement of ‘Unclear risk’ of bias  Insufficient information to permit judgement of ‘Low risk’ or ‘High risk’. | High risk | Quote: “Every patient who was interested to participate in the study has been allocated a random number (1,2,3) using Microsoft excel software. Therefore, each patient has been informed about the allocated intervention.”  An unconcealed procedure |
| Blinding of Participants And Personnel  (Performance Bias)  All Outcomes | Criteria for a judgement of ‘Low risk’ of bias   - No blinding or incomplete blinding, but the review authors judge that the outcome is not likely to be influenced by lack of blinding; - Blinding of participants and key study personnel ensured, and unlikely that the blinding could have been broken.   Criteria for the judgement of ‘High risk’ of bias   - No blinding or incomplete blinding, and the outcome is likely to be influenced by lack of blinding; - Blinding of key study participants and personnel attempted, but likely that the blinding could have been broken, and the outcome is likely to be influenced by lack of blinding.   Criteria for the judgement of ‘Unclear risk’ of bias   - Insufficient information to permit judgement of ‘Low risk’ or ‘High risk’; - The study did not address this outcome | Low risk | Quote: “Before starting the intervention phase, the participants were, blind to pre-test results, randomly assigned to one of the following groups: The first group received both cognitive and physical exercise training (physical exercise group, n = 16); the second group underwent cognitive and relaxation training (relaxation group, n = 17). In addition to the intervention groups, we had a ‘waiting control group,’ including n = 18 participants, to control for potential bias factors” |
| Blinding of Outcome Assessment  (Detection Bias)  All Outcomes | Criteria for a judgement of ‘Low risk’ of bias   - No blinding of outcome assessment, but the review authors judge that the outcome measurement is not likely to be influenced by lack of blinding; - Blinding of outcome assessment ensured, and unlikely that the blinding could have been broken.   Criteria for the judgement of ‘High risk’ of bias   - No blinding of outcome assessment, and the outcome measurement is likely to be influenced by lack of blinding; - Blinding of outcome assessment, but likely that the blinding could have been broken, and the outcome measurement is likely to be influenced by lack of blinding.   Criteria for the judgement of ‘Unclear risk’ of bias   - Insufficient information to permit judgement of ‘Low risk’ or ‘High risk’; - The study did not address this outcome | Low risk | Quote: “Assessors were  blind to treatment group.” |
| Incomplete Outcome Data  (Attrition Bias)  All Outcomes | Criteria for a judgement of ‘Low risk’ of bias   - No missing outcome data; - Reasons for missing outcome data unlikely to be related to true outcome (for survival data, censoring unlikely to be introducing bias); - Missing outcome data balanced in numbers across intervention groups, with similar reasons for missing data across groups; - For dichotomous outcome data, the proportion of missing outcomes compared with observed event risk not enough to have a clinically relevant impact on the intervention effect estimate; - For continuous outcome data, plausible effect size (difference in means or standardized difference in means) among missing outcomes not enough to have a clinically relevant impact on observed effect size; - Missing data have been imputed using appropriate methods.   Criteria for the judgement of ‘High risk’ of bias   - Reason for missing outcome data likely to be related to true outcome, with either imbalance in numbers or reasons for missing data across intervention groups; - For dichotomous outcome data, the proportion of missing outcomes compared with observed event risk enough to induce clinically relevant bias in intervention effect estimate; - For continuous outcome data, plausible effect size (difference in means or standardized difference in means) among missing outcomes enough to induce clinically relevant bias in observed effect size; - ‘As-treated’ analysis done with substantial departure of the intervention received from that assigned at randomization; - Potentially inappropriate application of simple imputation.   Criteria for the judgement of ‘Unclear risk’ of bias   - Insufficient information to permit judgement of ‘Low risk’ or ‘High risk’; - The study did not address this outcome | Low risk | Quote: “Initially, n = 75 patients took part in the study, but only fifty-one patients completed the intervention (drop-out rate  32 %). The drop-out rate was equally distributed across disease and intervention groups. … We conducted an additional ITT with all scores, which changed significantly between pre- and post-testing. To perform the ITT, we replaced all missing values in the post-testing with the mean of the respective group (‘mean replacement’) and repeated the analyses. The results of the ITT indicated that the previous significant results for the cognitive testing and the individual psychopathology remained significant without the results of the state anxiety (STAI), which was no longer significant (p[0.05).” |
| Selective Reporting (Reporting Bias) | Criteria for a judgement of ‘Low risk’ of bias   - The study protocol is available and all of the study’s pre-specified (primary and secondary) outcomes that are of interest in the review have been reported in the pre-specified way; - The study protocol is not available but it is clear that the published reports include all expected outcomes, including those that were pre-specified (convincing text of this nature may be uncommon).   Criteria for the judgement of ‘High risk’ of bias   - Not all of the study’s pre-specified primary outcomes have been reported; - One or more primary outcomes is reported using measurements, analysis methods or subsets of the data (e.g. subscales) that were not pre-specified; - One or more reported primary outcomes were not pre-specified (unless clear justification for their reporting is provided, such as an unexpected adverse effect); - One or more outcomes of interest in the review are reported incompletely so that they cannot be entered in a meta-analysis; - The study report fails to include results for a key outcome that would be expected to have been reported for such a study   Criteria for the judgement of ‘Unclear risk’ of bias  Insufficient information to permit judgement of ‘Low risk’ or ‘High risk’. It is likely that the majority of studies will fall into this category. | High risk | Comments: A study protocol is not available therefore, unable to determine if all of the study’s pre-specified primary outcomes were reported. |
| Other Bias | Criteria for a judgement of ‘Low risk’ of bias  Trials not published in the list of suspected predatory journals presented by Manca et al. 2017.  Criteria for the judgement of ‘High risk’ of bias  Trials published in the list of suspected predatory journals presented by Manca et al. 2017. | Low Risk | comments: The trial is not published in the list of suspected predatory journals presented by Manca et al. 2017. |

**Sailer et al. 2015**

| Bias | Criteria for judging risk of bias in the Risk of Bias assessment tool | Authors’ judgement | Support for judgement |
| --- | --- | --- | --- |
|  |  |  |  |
| Random Sequence Generation  (Selection Bias) | Criteria for a judgement of ‘Low risk’ of bias  The investigators describe a random component in the sequence generation process such as:   - Referring to a random number table; - Using a computer random number generator; - Coin tossing; - Shuffling cards or envelopes; - Throwing dice; - Drawing of lots; - Minimization.   Criteria for the judgement of ‘High risk’ of bias  The investigators describe a non-random component in the sequence generation process. Usually, the description would involve some systematic, non-random approach, for example:   - Sequence generated by odd or even date of birth; - Sequence generated by some rule based on date (or day) of admission; - Sequence generated by some rule based on hospital or clinic record number.   Other non-random approaches happen much less frequently than the systematic approaches mentioned above and tend to be obvious.  They usually involve judgement or some method of non-random categorization of participants, for example:   - Allocation by judgement of the clinician; - Allocation by preference of the participant; - Allocation based on the results of a laboratory test or a series of tests; - Allocation by availability of the intervention   Criteria for the judgement of ‘Unclear risk’ of bias  Insufficient information about the sequence generation process to permit judgement of ‘Low risk’ or ‘High risk’. | Unclear risk | Quote: “To assure proper  randomization, the therapists were provided with closed and identical envelopes that contained materials for either the experimental or control condition and were asked to pick one envelope for each patient.”  Comment: It’s not clear how the allocation sequence was generated |
| Allocation Concealment (Selection Bias) | Criteria for a judgement of ‘Low risk’ of bias  Participants and investigators enrolling participants could not foresee assignment because one of the following, or an equivalent method, was used to conceal allocation:   - Central allocation (including telephone, web-based and pharmacy-controlled randomization); - Sequentially numbered drug containers of identical appearance; - Sequentially numbered, opaque, sealed envelopes.   Criteria for the judgement of ‘High risk’ of bias  Participants or investigators enrolling participants could possibly foresee assignments and thus introduce selection bias, such as allocation based on:   - Using an open random allocation schedule (e.g. a list of random numbers); - Assignment envelopes were used without appropriate safeguards (e.g. if envelopes were unsealed or non­opaque or not sequentially numbered); - Alternation or rotation; - Date of birth; - Case record number; - Any other explicitly unconcealed procedure.   Criteria for the judgement of ‘Unclear risk’ of bias  Insufficient information to permit judgement of ‘Low risk’ or ‘High risk’. | Low risk | Quote: “To assure proper  randomization, the therapists were provided with closed and identical envelopes that contained materials for either the experimental or control condition and were asked to pick one envelope for each patient.” |
| Blinding of Participants And Personnel  (Performance Bias)  All Outcomes | Criteria for a judgement of ‘Low risk’ of bias   - No blinding or incomplete blinding, but the review authors judge that the outcome is not likely to be influenced by lack of blinding; - Blinding of participants and key study personnel ensured, and unlikely that the blinding could have been broken.   Criteria for the judgement of ‘High risk’ of bias   - No blinding or incomplete blinding, and the outcome is likely to be influenced by lack of blinding; - Blinding of key study participants and personnel attempted, but likely that the blinding could have been broken, and the outcome is likely to be influenced by lack of blinding.   Criteria for the judgement of ‘Unclear risk’ of bias   - Insufficient information to permit judgement of ‘Low risk’ or ‘High risk’; - The study did not address this outcome | Low risk | Quote: “The individuals conducting the exercise sessions, the nursing staff, and the researchers did not know the treatment  condition.” |
| Blinding of Outcome Assessment  (Detection Bias)  All Outcomes | Criteria for a judgement of ‘Low risk’ of bias   - No blinding of outcome assessment, but the review authors judge that the outcome measurement is not likely to be influenced by lack of blinding; - Blinding of outcome assessment ensured, and unlikely that the blinding could have been broken.   Criteria for the judgement of ‘High risk’ of bias   - No blinding of outcome assessment, and the outcome measurement is likely to be influenced by lack of blinding; - Blinding of outcome assessment, but likely that the blinding could have been broken, and the outcome measurement is likely to be influenced by lack of blinding.   Criteria for the judgement of ‘Unclear risk’ of bias   - Insufficient information to permit judgement of ‘Low risk’ or ‘High risk’; - The study did not address this outcome | Low risk | Quote: “the double-blinded randomized and controlled design and the robustness of the results strengthen the confidence in the  observed effects”  Comment: Double-blinded suggesting that the outcome assessor was also blinded |
| Incomplete Outcome Data  (Attrition Bias)  All Outcomes | Criteria for a judgement of ‘Low risk’ of bias   - No missing outcome data; - Reasons for missing outcome data unlikely to be related to true outcome (for survival data, censoring unlikely to be introducing bias); - Missing outcome data balanced in numbers across intervention groups, with similar reasons for missing data across groups; - For dichotomous outcome data, the proportion of missing outcomes compared with observed event risk not enough to have a clinically relevant impact on the intervention effect estimate; - For continuous outcome data, plausible effect size (difference in means or standardized difference in means) among missing outcomes not enough to have a clinically relevant impact on observed effect size; - Missing data have been imputed using appropriate methods.   Criteria for the judgement of ‘High risk’ of bias   - Reason for missing outcome data likely to be related to true outcome, with either imbalance in numbers or reasons for missing data across intervention groups; - For dichotomous outcome data, the proportion of missing outcomes compared with observed event risk enough to induce clinically relevant bias in intervention effect estimate; - For continuous outcome data, plausible effect size (difference in means or standardized difference in means) among missing outcomes enough to induce clinically relevant bias in observed effect size; - ‘As-treated’ analysis done with substantial departure of the intervention received from that assigned at randomization; - Potentially inappropriate application of simple imputation.   Criteria for the judgement of ‘Unclear risk’ of bias   - Insufficient information to permit judgement of ‘Low risk’ or ‘High risk’; - The study did not address this outcome | High risk | Comment: Several participants did not attend the week 4 study assessment. No ITT analysis was described. |
| Selective Reporting (Reporting Bias) | Criteria for a judgement of ‘Low risk’ of bias   - The study protocol is available and all of the study’s pre-specified (primary and secondary) outcomes that are of interest in the review have been reported in the pre-specified way; - The study protocol is not available but it is clear that the published reports include all expected outcomes, including those that were pre-specified (convincing text of this nature may be uncommon).   Criteria for the judgement of ‘High risk’ of bias   - Not all of the study’s pre-specified primary outcomes have been reported; - One or more primary outcomes is reported using measurements, analysis methods or subsets of the data (e.g. subscales) that were not pre-specified; - One or more reported primary outcomes were not pre-specified (unless clear justification for their reporting is provided, such as an unexpected adverse effect); - One or more outcomes of interest in the review are reported incompletely so that they cannot be entered in a meta-analysis; - The study report fails to include results for a key outcome that would be expected to have been reported for such a study   Criteria for the judgement of ‘Unclear risk’ of bias  Insufficient information to permit judgement of ‘Low risk’ or ‘High risk’. It is likely that the majority of studies will fall into this category. | Low risk | Quote: “ClinicalTrials.gov ID; URL: NCT01547026 Registered 3 March 2012.” |
| Other Bias | Criteria for a judgement of ‘Low risk’ of bias  Trials not published in the list of suspected predatory journals presented by Manca et al. 2017.  Criteria for the judgement of ‘High risk’ of bias  Trials published in the list of suspected predatory journals presented by Manca et al. 2017. | Low Risk | comments: The trial is not published in the list of suspected predatory journals presented by Manca et al. 2017. |

**Schewee et al., 2013**

| Bias | Criteria for judging risk of bias in the Risk of Bias assessment tool | Authors’ judgement | Support for judgement |
| --- | --- | --- | --- |
|  |  |  |  |
| Random Sequence Generation  (Selection Bias) | Criteria for a judgement of ‘Low risk’ of bias  The investigators describe a random component in the sequence generation process such as:   - Referring to a random number table; - Using a computer random number generator; - Coin tossing; - Shuffling cards or envelopes; - Throwing dice; - Drawing of lots; - Minimization.   Criteria for the judgement of ‘High risk’ of bias  The investigators describe a non-random component in the sequence generation process. Usually, the description would involve some systematic, non-random approach, for example:   - Sequence generated by odd or even date of birth; - Sequence generated by some rule based on date (or day) of admission; - Sequence generated by some rule based on hospital or clinic record number.   Other non-random approaches happen much less frequently than the systematic approaches mentioned above and tend to be obvious.  They usually involve judgement or some method of non-random categorization of participants, for example:   - Allocation by judgement of the clinician; - Allocation by preference of the participant; - Allocation based on the results of a laboratory test or a series of tests; - Allocation by availability of the intervention   Criteria for the judgement of ‘Unclear risk’ of bias  Insufficient information about the sequence generation process to permit judgement of ‘Low risk’ or ‘High risk’. | Low risk | Quote: “After baseline measurements,  a computer-generated randomisation procedure,  incorporating concealed allocation (ratio  1 : 1), was followed with stratification for gender, location and body mass index” |
| Allocation Concealment (Selection Bias) | Criteria for a judgement of ‘Low risk’ of bias  Participants and investigators enrolling participants could not foresee assignment because one of the following, or an equivalent method, was used to conceal allocation:   - Central allocation (including telephone, web-based and pharmacy-controlled randomization); - Sequentially numbered drug containers of identical appearance; - Sequentially numbered, opaque, sealed envelopes.   Criteria for the judgement of ‘High risk’ of bias  Participants or investigators enrolling participants could possibly foresee assignments and thus introduce selection bias, such as allocation based on:   - Using an open random allocation schedule (e.g. a list of random numbers); - Assignment envelopes were used without appropriate safeguards (e.g. if envelopes were unsealed or non­opaque or not sequentially numbered); - Alternation or rotation; - Date of birth; - Case record number; - Any other explicitly unconcealed procedure.   Criteria for the judgement of ‘Unclear risk’ of bias  Insufficient information to permit judgement of ‘Low risk’ or ‘High risk’. | Low risk | Quote: “After baseline measurements,  a computer-generated randomisation procedure,  incorporating concealed allocation (ratio  1 : 1), was followed with stratification for gender, location and body mass index” |
| Blinding of Participants And Personnel  (Performance Bias)  All Outcomes | Criteria for a judgement of ‘Low risk’ of bias   - No blinding or incomplete blinding, but the review authors judge that the outcome is not likely to be influenced by lack of blinding; - Blinding of participants and key study personnel ensured, and unlikely that the blinding could have been broken.   Criteria for the judgement of ‘High risk’ of bias   - No blinding or incomplete blinding, and the outcome is likely to be influenced by lack of blinding; - Blinding of key study participants and personnel attempted, but likely that the blinding could have been broken, and the outcome is likely to be influenced by lack of blinding.   Criteria for the judgement of ‘Unclear risk’ of bias   - Insufficient information to permit judgement of ‘Low risk’ or ‘High risk’; - The study did not address this outcome | High risk | Comment: Blinding of participants and personnel was not possible due to nature of intervention. |
| Blinding of Outcome Assessment  (Detection Bias)  All Outcomes | Criteria for a judgement of ‘Low risk’ of bias   - No blinding of outcome assessment, but the review authors judge that the outcome measurement is not likely to be influenced by lack of blinding; - Blinding of outcome assessment ensured, and unlikely that the blinding could have been broken.   Criteria for the judgement of ‘High risk’ of bias   - No blinding of outcome assessment, and the outcome measurement is likely to be influenced by lack of blinding; - Blinding of outcome assessment, but likely that the blinding could have been broken, and the outcome measurement is likely to be influenced by lack of blinding.   Criteria for the judgement of ‘Unclear risk’ of bias   - Insufficient information to permit judgement of ‘Low risk’ or ‘High risk’; - The study did not address this outcome | Low risk | Quote: “All baseline and follow-up measurements (after 6 months of intervention) were assessed by a research assistant and sports physician, blinded to randomization” |
| Incomplete Outcome Data  (Attrition Bias)  All Outcomes | Criteria for a judgement of ‘Low risk’ of bias   - No missing outcome data; - Reasons for missing outcome data unlikely to be related to true outcome (for survival data, censoring unlikely to be introducing bias); - Missing outcome data balanced in numbers across intervention groups, with similar reasons for missing data across groups; - For dichotomous outcome data, the proportion of missing outcomes compared with observed event risk not enough to have a clinically relevant impact on the intervention effect estimate; - For continuous outcome data, plausible effect size (difference in means or standardized difference in means) among missing outcomes not enough to have a clinically relevant impact on observed effect size; - Missing data have been imputed using appropriate methods.   Criteria for the judgement of ‘High risk’ of bias   - Reason for missing outcome data likely to be related to true outcome, with either imbalance in numbers or reasons for missing data across intervention groups; - For dichotomous outcome data, the proportion of missing outcomes compared with observed event risk enough to induce clinically relevant bias in intervention effect estimate; - For continuous outcome data, plausible effect size (difference in means or standardized difference in means) among missing outcomes enough to induce clinically relevant bias in observed effect size; - ‘As-treated’ analysis done with substantial departure of the intervention received from that assigned at randomization; - Potentially inappropriate application of simple imputation.   Criteria for the judgement of ‘Unclear risk’ of bias   - Insufficient information to permit judgement of ‘Low risk’ or ‘High risk’; - The study did not address this outcome | Low risk | Quote: “Analyses were performed on intention-to-treat basis as well as per protocol. Intention-to-treat analyses included all subjects that were randomised, making efforts to obtain outcome data for all participating subjects, and analysing data for those patients with follow-up outcome data, disregarding missing data” |
| Selective Reporting (Reporting Bias) | Criteria for a judgement of ‘Low risk’ of bias   - The study protocol is available and all of the study’s pre-specified (primary and secondary) outcomes that are of interest in the review have been reported in the pre-specified way; - The study protocol is not available but it is clear that the published reports include all expected outcomes, including those that were pre-specified (convincing text of this nature may be uncommon).   Criteria for the judgement of ‘High risk’ of bias   - Not all of the study’s pre-specified primary outcomes have been reported; - One or more primary outcomes is reported using measurements, analysis methods or subsets of the data (e.g. subscales) that were not pre-specified; - One or more reported primary outcomes were not pre-specified (unless clear justification for their reporting is provided, such as an unexpected adverse effect); - One or more outcomes of interest in the review are reported incompletely so that they cannot be entered in a meta-analysis; - The study report fails to include results for a key outcome that would be expected to have been reported for such a study   Criteria for the judgement of ‘Unclear risk’ of bias  Insufficient information to permit judgement of ‘Low risk’ or ‘High risk’. It is likely that the majority of studies will fall into this category. | Low risk | Quote: “This randomised controlled trial was registered in the ISRCTN register (http://www.controlled-trials.com/ISRCTN46241817/).” |
| Other Bias | Criteria for a judgement of ‘Low risk’ of bias  Trials not published in the list of suspected predatory journals presented by Manca et al. 2017.  Criteria for the judgement of ‘High risk’ of bias  Trials published in the list of suspected predatory journals presented by Manca et al. 2017. | Low Risk | comments: The trial is not published in the list of suspected predatory journals presented by Manca et al. 2017. |

**Shimada et al. 2020**

| Bias | Criteria for judging risk of bias in the Risk of Bias assessment tool | Authors’ judgement | Support for judgement |
| --- | --- | --- | --- |
|  |  |  |  |
| Random Sequence Generation  (Selection Bias) | Criteria for a judgement of ‘Low risk’ of bias  The investigators describe a random component in the sequence generation process such as:   - Referring to a random number table; - Using a computer random number generator; - Coin tossing; - Shuffling cards or envelopes; - Throwing dice; - Drawing of lots; - Minimization.   Criteria for the judgement of ‘High risk’ of bias  The investigators describe a non-random component in the sequence generation process. Usually, the description would involve some systematic, non-random approach, for example:   - Sequence generated by odd or even date of birth; - Sequence generated by some rule based on date (or day) of admission; - Sequence generated by some rule based on hospital or clinic record number.   Other non-random approaches happen much less frequently than the systematic approaches mentioned above and tend to be obvious.  They usually involve judgement or some method of non-random categorization of participants, for example:   - Allocation by judgement of the clinician; - Allocation by preference of the participant; - Allocation based on the results of a laboratory test or a series of tests; - Allocation by availability of the intervention   Criteria for the judgement of ‘Unclear risk’ of bias  Insufficient information about the sequence generation process to permit judgement of ‘Low risk’ or ‘High risk’. | Low risk | Quote: “Randomization was stratified by sex (male/female) and age (20–29, 30–39, 40–49, 50–59, and 60–65 years) using a computer-generated randomization program. Within each stratum, patients were randomized 1:1 to the TAU + AE or the TAU alone group” |
| Allocation Concealment (Selection Bias) | Criteria for a judgement of ‘Low risk’ of bias  Participants and investigators enrolling participants could not foresee assignment because one of the following, or an equivalent method, was used to conceal allocation:   - Central allocation (including telephone, web-based and pharmacy-controlled randomization); - Sequentially numbered drug containers of identical appearance; - Sequentially numbered, opaque, sealed envelopes.   Criteria for the judgement of ‘High risk’ of bias  Participants or investigators enrolling participants could possibly foresee assignments and thus introduce selection bias, such as allocation based on:   - Using an open random allocation schedule (e.g. a list of random numbers); - Assignment envelopes were used without appropriate safeguards (e.g. if envelopes were unsealed or non­opaque or not sequentially numbered); - Alternation or rotation; - Date of birth; - Case record number; - Any other explicitly unconcealed procedure.   Criteria for the judgement of ‘Unclear risk’ of bias  Insufficient information to permit judgement of ‘Low risk’ or ‘High risk’. | Unclear risk | Quote: “Randomization was stratified by sex (male/female) and age (20–29, 30–39, 40–49, 50–59, and 60–65 years) using a computer-generated randomization program. Within each stratum, patients were randomized 1:1 to the TAU + AE or the TAU alone group”  Not clear if this is central allocation |
| Blinding of Participants And Personnel  (Performance Bias)  All Outcomes | Criteria for a judgement of ‘Low risk’ of bias   - No blinding or incomplete blinding, but the review authors judge that the outcome is not likely to be influenced by lack of blinding; - Blinding of participants and key study personnel ensured, and unlikely that the blinding could have been broken.   Criteria for the judgement of ‘High risk’ of bias   - No blinding or incomplete blinding, and the outcome is likely to be influenced by lack of blinding; - Blinding of key study participants and personnel attempted, but likely that the blinding could have been broken, and the outcome is likely to be influenced by lack of blinding.   Criteria for the judgement of ‘Unclear risk’ of bias   - Insufficient information to permit judgement of ‘Low risk’ or ‘High risk’; - The study did not address this outcome | High risk | Comment: Blinding of participants and personnel was not possible due to nature of intervention. |
| Blinding of Outcome Assessment  (Detection Bias)  All Outcomes | Criteria for a judgement of ‘Low risk’ of bias   - No blinding of outcome assessment, but the review authors judge that the outcome measurement is not likely to be influenced by lack of blinding; - Blinding of outcome assessment ensured, and unlikely that the blinding could have been broken.   Criteria for the judgement of ‘High risk’ of bias   - No blinding of outcome assessment, and the outcome measurement is likely to be influenced by lack of blinding; - Blinding of outcome assessment, but likely that the blinding could have been broken, and the outcome measurement is likely to be influenced by lack of blinding.   Criteria for the judgement of ‘Unclear risk’ of bias   - Insufficient information to permit judgement of ‘Low risk’ or ‘High risk’; - The study did not address this outcome | Low risk | Quote: “Assessments were conducted by trained evaluators who were blinded to treatment assignment” |
| Incomplete Outcome Data  (Attrition Bias)  All Outcomes | Criteria for a judgement of ‘Low risk’ of bias   - No missing outcome data; - Reasons for missing outcome data unlikely to be related to true outcome (for survival data, censoring unlikely to be introducing bias); - Missing outcome data balanced in numbers across intervention groups, with similar reasons for missing data across groups; - For dichotomous outcome data, the proportion of missing outcomes compared with observed event risk not enough to have a clinically relevant impact on the intervention effect estimate; - For continuous outcome data, plausible effect size (difference in means or standardized difference in means) among missing outcomes not enough to have a clinically relevant impact on observed effect size; - Missing data have been imputed using appropriate methods.   Criteria for the judgement of ‘High risk’ of bias   - Reason for missing outcome data likely to be related to true outcome, with either imbalance in numbers or reasons for missing data across intervention groups; - For dichotomous outcome data, the proportion of missing outcomes compared with observed event risk enough to induce clinically relevant bias in intervention effect estimate; - For continuous outcome data, plausible effect size (difference in means or standardized difference in means) among missing outcomes enough to induce clinically relevant bias in observed effect size; - ‘As-treated’ analysis done with substantial departure of the intervention received from that assigned at randomization; - Potentially inappropriate application of simple imputation.   Criteria for the judgement of ‘Unclear risk’ of bias   - Insufficient information to permit judgement of ‘Low risk’ or ‘High risk’; - The study did not address this outcome | Low risk | Quote: “One patient (3.13%) dropped  out of the TAU alone arm based on a withdrawal of consent to participate.  The follow-up sample used for the after 12-week intervention analyses consisted of 40 patients: 20 (50.00%) in the TAU + AE and 20 (50.00%) in the TAU alone arms. All patients completed 6- and 12- month follow-up assessments”  Comment: missing data is unlikely to change the outcomes of the study |
| Selective Reporting (Reporting Bias) | Criteria for a judgement of ‘Low risk’ of bias   - The study protocol is available and all of the study’s pre-specified (primary and secondary) outcomes that are of interest in the review have been reported in the pre-specified way; - The study protocol is not available but it is clear that the published reports include all expected outcomes, including those that were pre-specified (convincing text of this nature may be uncommon).   Criteria for the judgement of ‘High risk’ of bias   - Not all of the study’s pre-specified primary outcomes have been reported; - One or more primary outcomes is reported using measurements, analysis methods or subsets of the data (e.g. subscales) that were not pre-specified; - One or more reported primary outcomes were not pre-specified (unless clear justification for their reporting is provided, such as an unexpected adverse effect); - One or more outcomes of interest in the review are reported incompletely so that they cannot be entered in a meta-analysis; - The study report fails to include results for a key outcome that would be expected to have been reported for such a study   Criteria for the judgement of ‘Unclear risk’ of bias  Insufficient information to permit judgement of ‘Low risk’ or ‘High risk’. It is likely that the majority of studies will fall into this category. | Low risk | Quote: “The study was registered in the University Hospital Medical Information Network Clinical Trials Registry (UMIN000034910).” |
| Other Bias | Criteria for a judgement of ‘Low risk’ of bias  Trials not published in the list of suspected predatory journals presented by Manca et al. 2017.  Criteria for the judgement of ‘High risk’ of bias  Trials published in the list of suspected predatory journals presented by Manca et al. 2017. | Low Risk | comments: The trial is not published in the list of suspected predatory journals presented by Manca et al. 2017. |

**Su et al. 2016**

| Bias | Criteria for judging risk of bias in the Risk of Bias assessment tool | Authors’ judgement | Support for judgement |
| --- | --- | --- | --- |
|  |  |  |  |
| Random Sequence Generation  (Selection Bias) | Criteria for a judgement of ‘Low risk’ of bias  The investigators describe a random component in the sequence generation process such as:   - Referring to a random number table; - Using a computer random number generator; - Coin tossing; - Shuffling cards or envelopes; - Throwing dice; - Drawing of lots; - Minimization.   Criteria for the judgement of ‘High risk’ of bias  The investigators describe a non-random component in the sequence generation process. Usually, the description would involve some systematic, non-random approach, for example:   - Sequence generated by odd or even date of birth; - Sequence generated by some rule based on date (or day) of admission; - Sequence generated by some rule based on hospital or clinic record number.   Other non-random approaches happen much less frequently than the systematic approaches mentioned above and tend to be obvious.  They usually involve judgement or some method of non-random categorization of participants, for example:   - Allocation by judgement of the clinician; - Allocation by preference of the participant; - Allocation based on the results of a laboratory test or a series of tests; - Allocation by availability of the intervention   Criteria for the judgement of ‘Unclear risk’ of bias  Insufficient information about the sequence generation process to permit judgement of ‘Low risk’ or ‘High risk’. | High risk | Quote: “After baseline testing, participants were randomly allocated in the order in which they were enrolled into the study to participate in either AE or a stretching and toning control group. A computer-generated randomization scheme using block sizes of 4 and an allocation ratio of 1:1, with stratification for age(20–40 years and 41–60 years)and years of schooling was prepared by a research assistant who was not involved in the recruitment or treatment of the patients. Participants began their intervention within 2 weeks of randomization.” |
| Allocation Concealment (Selection Bias) | Criteria for a judgement of ‘Low risk’ of bias  Participants and investigators enrolling participants could not foresee assignment because one of the following, or an equivalent method, was used to conceal allocation:   - Central allocation (including telephone, web-based and pharmacy-controlled randomization); - Sequentially numbered drug containers of identical appearance; - Sequentially numbered, opaque, sealed envelopes.   Criteria for the judgement of ‘High risk’ of bias  Participants or investigators enrolling participants could possibly foresee assignments and thus introduce selection bias, such as allocation based on:   - Using an open random allocation schedule (e.g. a list of random numbers); - Assignment envelopes were used without appropriate safeguards (e.g. if envelopes were unsealed or non­opaque or not sequentially numbered); - Alternation or rotation; - Date of birth; - Case record number; - Any other explicitly unconcealed procedure.   Criteria for the judgement of ‘Unclear risk’ of bias  Insufficient information to permit judgement of ‘Low risk’ or ‘High risk’. | Low risk | Quote: “After baseline testing, participants were randomly allocated in the order in which they were enrolled into the study to participate in either AE or a stretching and toning control group. A computer-generated randomization scheme using block sizes of 4 and an allocation ratio of 1:1, with stratification for age(20–40 years and 41–60 years)and years of schooling was prepared by a research assistant who was not involved in the recruitment or treatment of the patients. Participants began their intervention within 2 weeks of randomization.” |
| Blinding of Participants And Personnel  (Performance Bias)  All Outcomes | Criteria for a judgement of ‘Low risk’ of bias   - No blinding or incomplete blinding, but the review authors judge that the outcome is not likely to be influenced by lack of blinding; - Blinding of participants and key study personnel ensured, and unlikely that the blinding could have been broken.   Criteria for the judgement of ‘High risk’ of bias   - No blinding or incomplete blinding, and the outcome is likely to be influenced by lack of blinding; - Blinding of key study participants and personnel attempted, but likely that the blinding could have been broken, and the outcome is likely to be influenced by lack of blinding.   Criteria for the judgement of ‘Unclear risk’ of bias   - Insufficient information to permit judgement of ‘Low risk’ or ‘High risk’; - The study did not address this outcome | High risk | Quote: “The trainer was blinded to the results of all assessments”  Comment: Blinding of participants and personnel was not possible due to nature of intervention. |
| Blinding of Outcome Assessment  (Detection Bias)  All Outcomes | Criteria for a judgement of ‘Low risk’ of bias   - No blinding of outcome assessment, but the review authors judge that the outcome measurement is not likely to be influenced by lack of blinding; - Blinding of outcome assessment ensured, and unlikely that the blinding could have been broken.   Criteria for the judgement of ‘High risk’ of bias   - No blinding of outcome assessment, and the outcome measurement is likely to be influenced by lack of blinding; - Blinding of outcome assessment, but likely that the blinding could have been broken, and the outcome measurement is likely to be influenced by lack of blinding.   Criteria for the judgement of ‘Unclear risk’ of bias   - Insufficient information to permit judgement of ‘Low risk’ or ‘High risk’; - The study did not address this outcome | Low risk | Quote: “Clinicians who assessed the outcome were unaware of treatment group assignment” |
| Incomplete Outcome Data  (Attrition Bias)  All Outcomes | Criteria for a judgement of ‘Low risk’ of bias   - No missing outcome data; - Reasons for missing outcome data unlikely to be related to true outcome (for survival data, censoring unlikely to be introducing bias); - Missing outcome data balanced in numbers across intervention groups, with similar reasons for missing data across groups; - For dichotomous outcome data, the proportion of missing outcomes compared with observed event risk not enough to have a clinically relevant impact on the intervention effect estimate; - For continuous outcome data, plausible effect size (difference in means or standardized difference in means) among missing outcomes not enough to have a clinically relevant impact on observed effect size; - Missing data have been imputed using appropriate methods.   Criteria for the judgement of ‘High risk’ of bias   - Reason for missing outcome data likely to be related to true outcome, with either imbalance in numbers or reasons for missing data across intervention groups; - For dichotomous outcome data, the proportion of missing outcomes compared with observed event risk enough to induce clinically relevant bias in intervention effect estimate; - For continuous outcome data, plausible effect size (difference in means or standardized difference in means) among missing outcomes enough to induce clinically relevant bias in observed effect size; - ‘As-treated’ analysis done with substantial departure of the intervention received from that assigned at randomization; - Potentially inappropriate application of simple imputation.   Criteria for the judgement of ‘Unclear risk’ of bias   - Insufficient information to permit judgement of ‘Low risk’ or ‘High risk’; - The study did not address this outcome | High risk | Quote: “Eight (26.7%) in the AE and 5 (18.5%) in the control group dropped out of the study before completing the intervention”  Comment: No ITT analysis was described |
| Selective Reporting (Reporting Bias) | Criteria for a judgement of ‘Low risk’ of bias   - The study protocol is available and all of the study’s pre-specified (primary and secondary) outcomes that are of interest in the review have been reported in the pre-specified way; - The study protocol is not available but it is clear that the published reports include all expected outcomes, including those that were pre-specified (convincing text of this nature may be uncommon).   Criteria for the judgement of ‘High risk’ of bias   - Not all of the study’s pre-specified primary outcomes have been reported; - One or more primary outcomes is reported using measurements, analysis methods or subsets of the data (e.g. subscales) that were not pre-specified; - One or more reported primary outcomes were not pre-specified (unless clear justification for their reporting is provided, such as an unexpected adverse effect); - One or more outcomes of interest in the review are reported incompletely so that they cannot be entered in a meta-analysis; - The study report fails to include results for a key outcome that would be expected to have been reported for such a study   Criteria for the judgement of ‘Unclear risk’ of bias  Insufficient information to permit judgement of ‘Low risk’ or ‘High risk’. It is likely that the majority of studies will fall into this category. | High risk | Comments: A study protocol is not available therefore, unable to determine if all of the study’s pre-specified primary outcomes were reported. |
| Other Bias | Criteria for a judgement of ‘Low risk’ of bias  Trials not published in the list of suspected predatory journals presented by Manca et al. 2017.  Criteria for the judgement of ‘High risk’ of bias  Trials published in the list of suspected predatory journals presented by Manca et al. 2017. | Low Risk | comments: The trial is not published in the list of suspected predatory journals presented by Manca et al. 2017. |

**Varambally et al., 2012**

| Bias | Criteria for judging risk of bias in the Risk of Bias assessment tool | Authors’ judgement | Support for judgement |
| --- | --- | --- | --- |
|  |  |  |  |
| Random Sequence Generation  (Selection Bias) | Criteria for a judgement of ‘Low risk’ of bias  The investigators describe a random component in the sequence generation process such as:   - Referring to a random number table; - Using a computer random number generator; - Coin tossing; - Shuffling cards or envelopes; - Throwing dice; - Drawing of lots; - Minimization.   Criteria for the judgement of ‘High risk’ of bias  The investigators describe a non-random component in the sequence generation process. Usually, the description would involve some systematic, non-random approach, for example:   - Sequence generated by odd or even date of birth; - Sequence generated by some rule based on date (or day) of admission; - Sequence generated by some rule based on hospital or clinic record number.   Other non-random approaches happen much less frequently than the systematic approaches mentioned above and tend to be obvious.  They usually involve judgement or some method of non-random categorization of participants, for example:   - Allocation by judgement of the clinician; - Allocation by preference of the participant; - Allocation based on the results of a laboratory test or a series of tests; - Allocation by availability of the intervention   Criteria for the judgement of ‘Unclear risk’ of bias  Insufficient information about the sequence generation process to permit judgement of ‘Low risk’ or ‘High risk’. | Low risk | Quote: “One investigator (JT) uninvolved in the treatments or assessments generated random numbers for 120 patients to be allocated to three groups of approximately equal numbers. A subject’s allocation to one of these groups was kept concealed and was ascertained only after he/she consented and when he/she was to be randomized” |
| Allocation Concealment (Selection Bias) | Criteria for a judgement of ‘Low risk’ of bias  Participants and investigators enrolling participants could not foresee assignment because one of the following, or an equivalent method, was used to conceal allocation:   - Central allocation (including telephone, web-based and pharmacy-controlled randomization); - Sequentially numbered drug containers of identical appearance; - Sequentially numbered, opaque, sealed envelopes.   Criteria for the judgement of ‘High risk’ of bias  Participants or investigators enrolling participants could possibly foresee assignments and thus introduce selection bias, such as allocation based on:   - Using an open random allocation schedule (e.g. a list of random numbers); - Assignment envelopes were used without appropriate safeguards (e.g. if envelopes were unsealed or non­opaque or not sequentially numbered); - Alternation or rotation; - Date of birth; - Case record number; - Any other explicitly unconcealed procedure.   Criteria for the judgement of ‘Unclear risk’ of bias  Insufficient information to permit judgement of ‘Low risk’ or ‘High risk’. | Low risk | Quote: “A subject’s allocation to one of these groups was kept concealed and was ascertained only after he/she consented and when he/she was to be randomized. Only the social worker and the yoga therapist in the study were  informed to start the corresponding intervention. The rest of the research team was unaware of the current group allocation. The three groups allocated were yogasana (n=47), exercise (n=37), and waitlist (n=36). Assessments  began after allocation.” |
| Blinding of Participants And Personnel  (Performance Bias)  All Outcomes | Criteria for a judgement of ‘Low risk’ of bias   - No blinding or incomplete blinding, but the review authors judge that the outcome is not likely to be influenced by lack of blinding; - Blinding of participants and key study personnel ensured, and unlikely that the blinding could have been broken.   Criteria for the judgement of ‘High risk’ of bias   - No blinding or incomplete blinding, and the outcome is likely to be influenced by lack of blinding; - Blinding of key study participants and personnel attempted, but likely that the blinding could have been broken, and the outcome is likely to be influenced by lack of blinding.   Criteria for the judgement of ‘Unclear risk’ of bias   - Insufficient information to permit judgement of ‘Low risk’ or ‘High risk’; - The study did not address this outcome | High risk | Quote: All patients were assessed by a blind rater at the start of the intervention and at the end of 4 months”  Comment: Blinding of participants was not possible due to nature of intervention. |
| Blinding of Outcome Assessment  (Detection Bias)  All Outcomes | Criteria for a judgement of ‘Low risk’ of bias   - No blinding of outcome assessment, but the review authors judge that the outcome measurement is not likely to be influenced by lack of blinding; - Blinding of outcome assessment ensured, and unlikely that the blinding could have been broken.   Criteria for the judgement of ‘High risk’ of bias   - No blinding of outcome assessment, and the outcome measurement is likely to be influenced by lack of blinding; - Blinding of outcome assessment, but likely that the blinding could have been broken, and the outcome measurement is likely to be influenced by lack of blinding.   Criteria for the judgement of ‘Unclear risk’ of bias   - Insufficient information to permit judgement of ‘Low risk’ or ‘High risk’; - The study did not address this outcome | Low risk | Quote: “All patients were assessed by a blind rater at the start of the intervention and at the end of 4 months” |
| Incomplete Outcome Data  (Attrition Bias)  All Outcomes | Criteria for a judgement of ‘Low risk’ of bias   - No missing outcome data; - Reasons for missing outcome data unlikely to be related to true outcome (for survival data, censoring unlikely to be introducing bias); - Missing outcome data balanced in numbers across intervention groups, with similar reasons for missing data across groups; - For dichotomous outcome data, the proportion of missing outcomes compared with observed event risk not enough to have a clinically relevant impact on the intervention effect estimate; - For continuous outcome data, plausible effect size (difference in means or standardized difference in means) among missing outcomes not enough to have a clinically relevant impact on observed effect size; - Missing data have been imputed using appropriate methods.   Criteria for the judgement of ‘High risk’ of bias   - Reason for missing outcome data likely to be related to true outcome, with either imbalance in numbers or reasons for missing data across intervention groups; - For dichotomous outcome data, the proportion of missing outcomes compared with observed event risk enough to induce clinically relevant bias in intervention effect estimate; - For continuous outcome data, plausible effect size (difference in means or standardized difference in means) among missing outcomes enough to induce clinically relevant bias in observed effect size; - ‘As-treated’ analysis done with substantial departure of the intervention received from that assigned at randomization; - Potentially inappropriate application of simple imputation.   Criteria for the judgement of ‘Unclear risk’ of bias   - Insufficient information to permit judgement of ‘Low risk’ or ‘High risk’; - The study did not address this outcome | High risk | Quote: “some did not turn up at the 4th month follow‑up and therefore the final sample was smaller; 39, 22, and 34 in yogasana, exercise, and waitlist groups, respectively”  Comment: This reflects a 20% drop out, no ITT analysis was described |
| Selective Reporting (Reporting Bias) | Criteria for a judgement of ‘Low risk’ of bias   - The study protocol is available and all of the study’s pre-specified (primary and secondary) outcomes that are of interest in the review have been reported in the pre-specified way; - The study protocol is not available but it is clear that the published reports include all expected outcomes, including those that were pre-specified (convincing text of this nature may be uncommon).   Criteria for the judgement of ‘High risk’ of bias   - Not all of the study’s pre-specified primary outcomes have been reported; - One or more primary outcomes is reported using measurements, analysis methods or subsets of the data (e.g. subscales) that were not pre-specified; - One or more reported primary outcomes were not pre-specified (unless clear justification for their reporting is provided, such as an unexpected adverse effect); - One or more outcomes of interest in the review are reported incompletely so that they cannot be entered in a meta-analysis; - The study report fails to include results for a key outcome that would be expected to have been reported for such a study   Criteria for the judgement of ‘Unclear risk’ of bias  Insufficient information to permit judgement of ‘Low risk’ or ‘High risk’. It is likely that the majority of studies will fall into this category. | High risk | Comments: A study protocol is not available therefore, unable to determine if all of the study’s pre-specified primary outcomes were reported. |
| Other Bias | Criteria for a judgement of ‘Low risk’ of bias  Trials not published in the list of suspected predatory journals presented by Manca et al. 2017.  Criteria for the judgement of ‘High risk’ of bias  Trials published in the list of suspected predatory journals presented by Manca et al. 2017. | Low Risk | comments: The trial is not published in the list of suspected predatory journals presented by Manca et al. 2017. |

**Wang et al., 2018**

| Bias | Criteria for judging risk of bias in the Risk of Bias assessment tool | Authors’ judgement | Support for judgement |
| --- | --- | --- | --- |
|  |  |  |  |
| Random Sequence Generation  (Selection Bias) | Criteria for a judgement of ‘Low risk’ of bias  The investigators describe a random component in the sequence generation process such as:   - Referring to a random number table; - Using a computer random number generator; - Coin tossing; - Shuffling cards or envelopes; - Throwing dice; - Drawing of lots; - Minimization.   Criteria for the judgement of ‘High risk’ of bias  The investigators describe a non-random component in the sequence generation process. Usually, the description would involve some systematic, non-random approach, for example:   - Sequence generated by odd or even date of birth; - Sequence generated by some rule based on date (or day) of admission; - Sequence generated by some rule based on hospital or clinic record number.   Other non-random approaches happen much less frequently than the systematic approaches mentioned above and tend to be obvious.  They usually involve judgement or some method of non-random categorization of participants, for example:   - Allocation by judgement of the clinician; - Allocation by preference of the participant; - Allocation based on the results of a laboratory test or a series of tests; - Allocation by availability of the intervention   Criteria for the judgement of ‘Unclear risk’ of bias  Insufficient information about the sequence generation process to permit judgement of ‘Low risk’ or ‘High risk’. | Unclear risk | Quote: “This study, which was conducted between 2012 and 2015, was  a single-blind randomized case-control trial. After screening and explaining the process of this study, all participants signed an informed consent form, following which they were  randomly allocated into an aerobic exercise (AE) group or a stretching control group.”  Comment: not clear how the randomization sequence was generated |
| Allocation Concealment (Selection Bias) | Criteria for a judgement of ‘Low risk’ of bias  Participants and investigators enrolling participants could not foresee assignment because one of the following, or an equivalent method, was used to conceal allocation:   - Central allocation (including telephone, web-based and pharmacy-controlled randomization); - Sequentially numbered drug containers of identical appearance; - Sequentially numbered, opaque, sealed envelopes.   Criteria for the judgement of ‘High risk’ of bias  Participants or investigators enrolling participants could possibly foresee assignments and thus introduce selection bias, such as allocation based on:   - Using an open random allocation schedule (e.g. a list of random numbers); - Assignment envelopes were used without appropriate safeguards (e.g. if envelopes were unsealed or non­opaque or not sequentially numbered); - Alternation or rotation; - Date of birth; - Case record number; - Any other explicitly unconcealed procedure.   Criteria for the judgement of ‘Unclear risk’ of bias  Insufficient information to permit judgement of ‘Low risk’ or ‘High risk’. | Unclear risk | Quote: “This study, which was conducted between 2012 and 2015, was  a single-blind randomized case-control trial. After screening and explaining the process of this study, all participants signed an informed consent form, following which they were  randomly allocated into an aerobic exercise (AE) group or a stretching control group.”  Comment: Allocation was not well described and therefore it’s unclear |
| Blinding of Participants And Personnel  (Performance Bias)  All Outcomes | Criteria for a judgement of ‘Low risk’ of bias   - No blinding or incomplete blinding, but the review authors judge that the outcome is not likely to be influenced by lack of blinding; - Blinding of participants and key study personnel ensured, and unlikely that the blinding could have been broken.   Criteria for the judgement of ‘High risk’ of bias   - No blinding or incomplete blinding, and the outcome is likely to be influenced by lack of blinding; - Blinding of key study participants and personnel attempted, but likely that the blinding could have been broken, and the outcome is likely to be influenced by lack of blinding.   Criteria for the judgement of ‘Unclear risk’ of bias   - Insufficient information to permit judgement of ‘Low risk’ or ‘High risk’; - The study did not address this outcome | High risk | Quote: “The psychiatrist who evaluated the participants was blind to their group assignments. The protocol was approved  by the Institutional Review Board of Kaohsiung Medical University”  Comment: This was a single blind study. Blinding of participants and personnel was not possible due to nature of intervention. |
| Blinding of Outcome Assessment  (Detection Bias)  All Outcomes | Criteria for a judgement of ‘Low risk’ of bias   - No blinding of outcome assessment, but the review authors judge that the outcome measurement is not likely to be influenced by lack of blinding; - Blinding of outcome assessment ensured, and unlikely that the blinding could have been broken.   Criteria for the judgement of ‘High risk’ of bias   - No blinding of outcome assessment, and the outcome measurement is likely to be influenced by lack of blinding; - Blinding of outcome assessment, but likely that the blinding could have been broken, and the outcome measurement is likely to be influenced by lack of blinding.   Criteria for the judgement of ‘Unclear risk’ of bias   - Insufficient information to permit judgement of ‘Low risk’ or ‘High risk’; - The study did not address this outcome | Low risk | Quote: “The psychiatrist who evaluated the participants was blind to their group assignments. The protocol was approved  by the Institutional Review Board of Kaohsiung Medical University” |
| Incomplete Outcome Data  (Attrition Bias)  All Outcomes | Criteria for a judgement of ‘Low risk’ of bias   - No missing outcome data; - Reasons for missing outcome data unlikely to be related to true outcome (for survival data, censoring unlikely to be introducing bias); - Missing outcome data balanced in numbers across intervention groups, with similar reasons for missing data across groups; - For dichotomous outcome data, the proportion of missing outcomes compared with observed event risk not enough to have a clinically relevant impact on the intervention effect estimate; - For continuous outcome data, plausible effect size (difference in means or standardized difference in means) among missing outcomes not enough to have a clinically relevant impact on observed effect size; - Missing data have been imputed using appropriate methods.   Criteria for the judgement of ‘High risk’ of bias   - Reason for missing outcome data likely to be related to true outcome, with either imbalance in numbers or reasons for missing data across intervention groups; - For dichotomous outcome data, the proportion of missing outcomes compared with observed event risk enough to induce clinically relevant bias in intervention effect estimate; - For continuous outcome data, plausible effect size (difference in means or standardized difference in means) among missing outcomes enough to induce clinically relevant bias in observed effect size; - ‘As-treated’ analysis done with substantial departure of the intervention received from that assigned at randomization; - Potentially inappropriate application of simple imputation.   Criteria for the judgement of ‘Unclear risk’ of bias   - Insufficient information to permit judgement of ‘Low risk’ or ‘High risk’; - The study did not address this outcome | High risk | Quote: “A total of 62 participants were therefore randomly assigned into either the aerobic exercise (AE) group (33 participants) or the control group (29 participants). Nine (27.27%) and seven participants (24.14%) in the AE and control groups did not complete the study, respectively.”  Comment: No ITT analysis was described |
| Selective Reporting (Reporting Bias) | Criteria for a judgement of ‘Low risk’ of bias   - The study protocol is available and all of the study’s pre-specified (primary and secondary) outcomes that are of interest in the review have been reported in the pre-specified way; - The study protocol is not available but it is clear that the published reports include all expected outcomes, including those that were pre-specified (convincing text of this nature may be uncommon).   Criteria for the judgement of ‘High risk’ of bias   - Not all of the study’s pre-specified primary outcomes have been reported; - One or more primary outcomes is reported using measurements, analysis methods or subsets of the data (e.g. subscales) that were not pre-specified; - One or more reported primary outcomes were not pre-specified (unless clear justification for their reporting is provided, such as an unexpected adverse effect); - One or more outcomes of interest in the review are reported incompletely so that they cannot be entered in a meta-analysis; - The study report fails to include results for a key outcome that would be expected to have been reported for such a study   Criteria for the judgement of ‘Unclear risk’ of bias  Insufficient information to permit judgement of ‘Low risk’ or ‘High risk’. It is likely that the majority of studies will fall into this category. | High risk | Comments: A study protocol is not available therefore, unable to determine if all of the study’s pre-specified primary outcomes were reported. |
| Other Bias | Criteria for a judgement of ‘Low risk’ of bias  Trials not published in the list of suspected predatory journals presented by Manca et al. 2017.  Criteria for the judgement of ‘High risk’ of bias  Trials published in the list of suspected predatory journals presented by Manca et al. 2017. | Low Risk | comments: The trial is not published in the list of suspected predatory journals presented by Manca et al. 2017. |

## Supplementary Table 4: Summary of Risk of bias assessment

|  | Random Sequence Generation | Allocation Concealment | Blinding of Participants and Personnel | Blinding of outcome assessment | Incomplete outcome data | Selective Reporting | Other Bias |
| --- | --- | --- | --- | --- | --- | --- | --- |
| Attux et al. 2013 | Low | Unclear | Unclear | Low | High | Low | Low |
| Bang-Kittilsen et al. 2020 | Low | Low | High | High | Low | Low | Low |
| Beebe et al. 2009 | Unclear | Unclear | Unclear | Unclear | High | High | Low |
| Brobakken et al. 2019 | Low | Unclear | High | High | Low | Low | Low |
| Curcic et al. 2017 | Unclear | Unclear | Unclear | Unclear | Unclear | High | Low |
| Duraiswamy et al. 2007 | Low | Unclear | Unclear | Unclear | Low | High | Low |
| Heggelung, et al., 2011 | High | High | High | High | High | Low | Low |
| Ikai et al. 2013 | Low | Low | High | Low | Low | Low | Low |
| Ikai et al., 2014 | Low | Low | High | Low | Low | Low | Low |
| Ikai et al., 2017 | Low | Low | High | Low | Low | Low | Low |
| Kaltsatou et al., 2015 | Low | Unclear | High | Low | Low | High | Low |
| Kurebayashi et al., 2021 | Low | Unclear | Low | High | Low | High | Low |
| Kwon et al. 2006 | Unclear | Unclear | High | High | Low | High | Low |
| Li et al. 2020 | Low | Low | High | Low | Low | High | Low |
| Loh et al.2016 | High | Low | High | Unclear | High | High | Low |
| Manjunath et al., 2013 | Unclear | Unclear | High | Low | High | High | Low |
| Methapatara et al. 2011 | Low | Low | High | High | Low | High | Low |
| Oertel-Knochel et al., 2014 | Low | High | Low | Low | Low | High | Low |
| Sailer et al. 2015 | Unclear | Low | Low | Low | High | Low | Low |
| Scheewe et al. 2013 | Low | Low | High | Low | Low | Low | Low |
| Shimada et al. 2020 | Low | Unclear | High | Low | Low | Low | Low |
| Su et al. 2016 | High | Low | High | Low | High | High | Low |
| Varambally et al., 2012 | Low | Low | High | Low | High | High | High |
| Wang et al., 2018 | Unclear | Unclear | High | Low | High |  |  |

# III. Supplementary Figures

## FIGURE S1. Medline Search Strategy

**
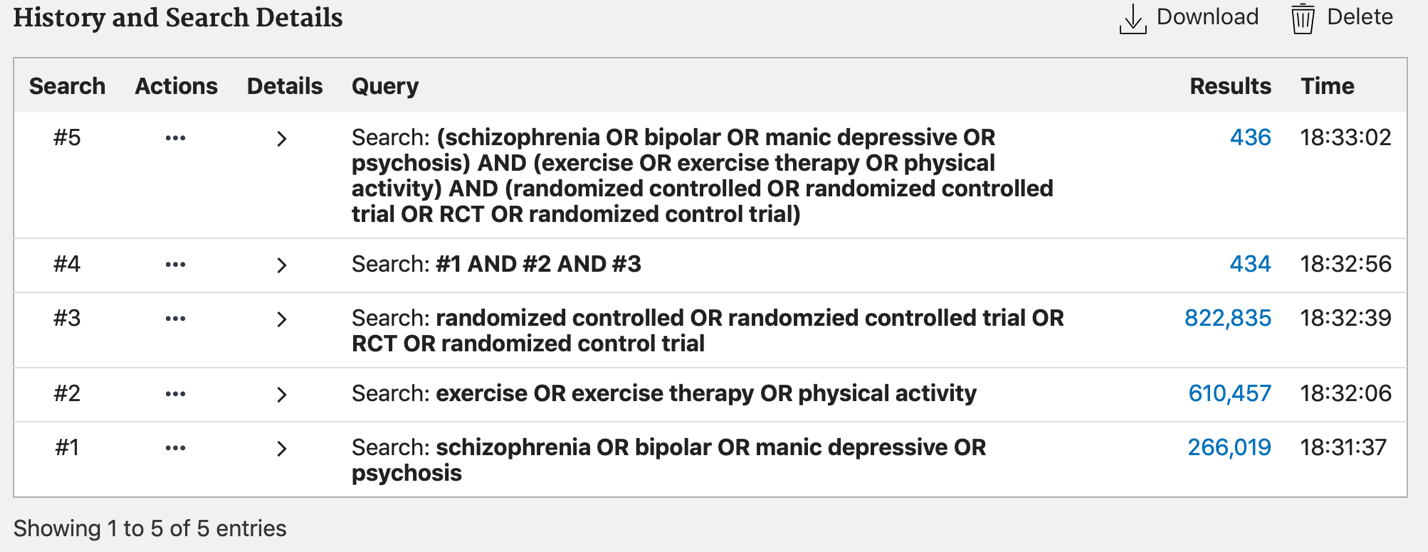
**

## FIGURE S2. Scopus search strategy

##


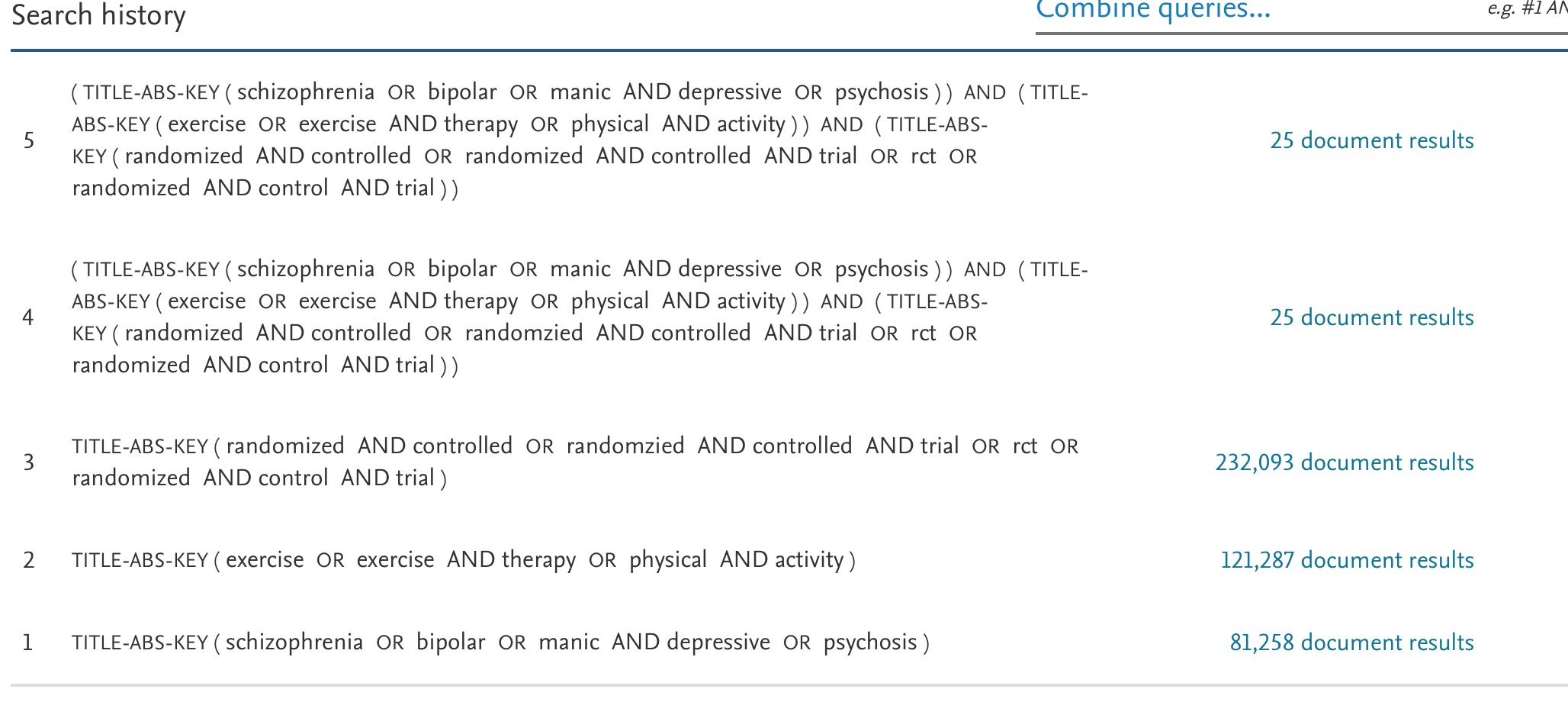


## FIGURE S3. PsychInfo (Ovid) search strategy

**
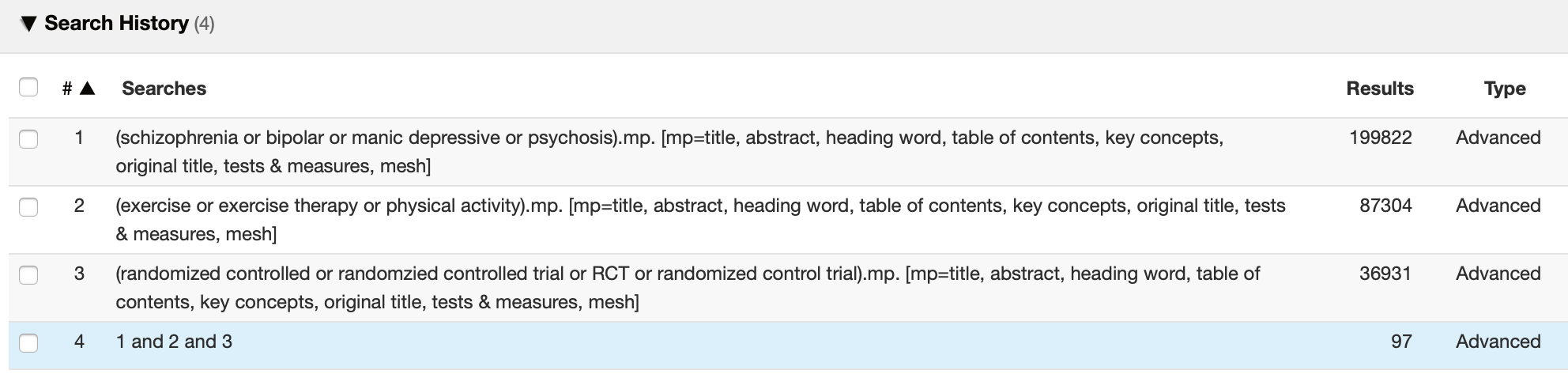
**

## FIGURE S4: Funnel plot showing publication bias
